# Synthesis of the I–K Fused Polyether Array of CTX3C and Related Ciguatoxins by Use of a Gold-Catalyzed Cyclization Reaction

**DOI:** 10.1021/acs.orglett.3c03782

**Published:** 2024-01-18

**Authors:** Venkaiah Chintalapudi, Claire Wilson, J. Stephen Clark

**Affiliations:** School of Chemistry, Joseph Black Building, University of Glasgow, University Avenue, Glasgow, G12 8QQ, U.K.

## Abstract

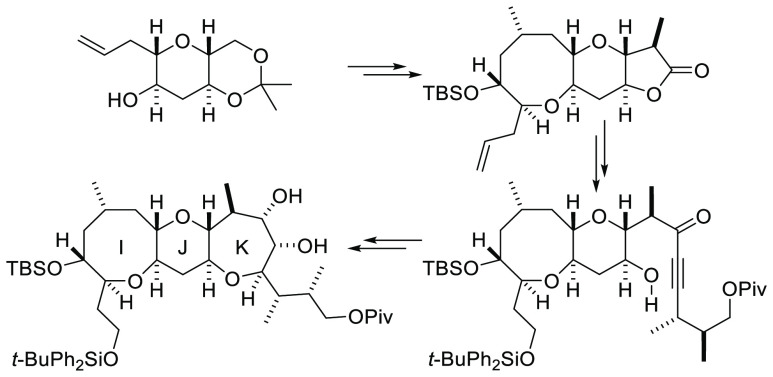

The I–K fragment (C31–C49) of the ciguatoxin CTX3C has been synthesized from a simple chiral pool derived tetrahydropyranyl alcohol. An efficient gold-catalyzed cyclization reaction of a γ′-hydroxy ynone has been used to accomplish efficient closure of ring K under mild conditions. The resulting vinylogous ester has been elaborated to give a complete tricyclic fragment bearing the dimethyl-substituted side chain required for assembly of the LM spirocyclic acetal portion of the target.

In 1993, Yasumoto and co-workers reported the characterization of CTX3C, a new and structurally complex fused polycyclic ether natural product isolated from cloned cells that had been prepared from a sample of the dinoflagellate *Gambierdiscus toxicus* originally collected in French Polynesia (Figure 1).^1^ CTX3C is one of more than 30 Pacific ciguatoxins isolated since 1990.^2^ Closely related but structurally distinct families of ciguatoxins have been isolated from organisms collected at locations in the Indian Ocean and Caribbean Sea, and it is likely that new ciguatoxins will be isolated from these and other locations in the future.^3^

**Figure 1:**
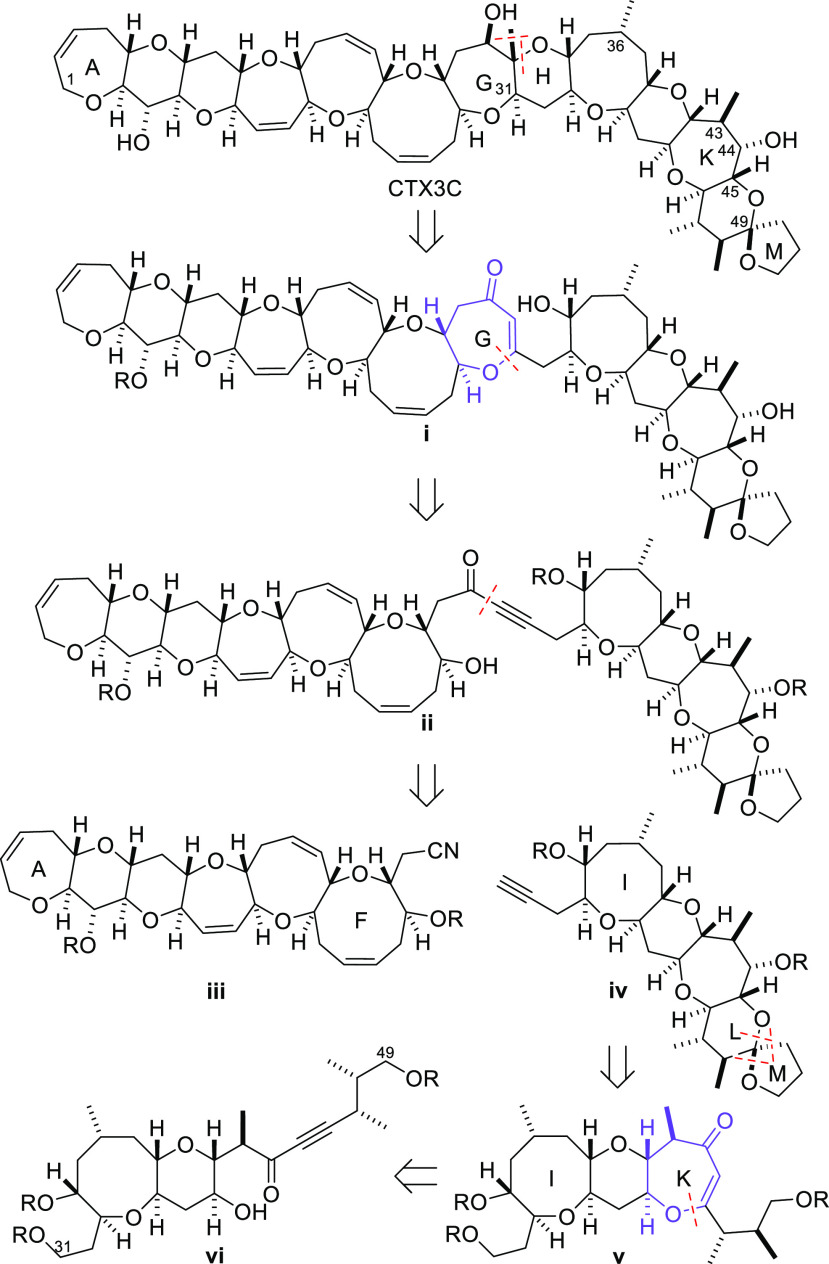
Retrosynthetic analysis of CTX3C.

CTX3C and other ciguatoxins are extremely potent neurotoxins that disrupt nerve signal transmission by interfering with voltage-gated sodium ion channels and their associated receptors.^4^ Along with related polyether toxins produced by *Gambierdiscus* dinoflagellates (e.g., maitotoxin), the ciguatoxins are responsible for ciguatera fish poisoning in humans. The consumption of fish and seafood contaminated with ciguatoxins is responsible for thousands of cases of food poisoning each year many of which result in severe illness or even death.^5^

Fused polycyclic ether natural products have been popular synthetic targets since the early 1980s, and the ciguatoxins have attracted particular attention.^6^ The size, structural complexity, bioactivity, and putative biosynthetic origins of the marine polyethers make them alluring targets for total synthesis, but only two research groups have completed total syntheses of members of the Pacific ciguatoxin family.^7,8^ The landmark synthesis of CTX3C was published by Hirama and co-workers in 2001, and syntheses of 51-hydroxy-CTX3C, CTX1B, and 54-deoxy-CTX1B were completed subsequently.^7^ Hamajima and Isobe completed an elegant total synthesis of CTX1B in 2009.^8^ Despite these ground-breaking syntheses, the total synthesis of any of the ciguatoxin natural products on the milligram scale remains a huge challenge that will require the development of new strategies and reactions that enable greater levels of convergence and efficiency.

A major focus of our research program is the development of new strategies for the efficient construction of fused polycyclic ether arrays of the type found as subunits in the ciguatoxins and related marine natural products. In previous work,^9,10^ we have used iterative and bidirectional strategies to assemble substantial portions of several marine polyethers; this work has culminated in the synthesis of the A–E and I–L fragments of CTX3C from chiral pool derived precursors.^10^ Very recently, we synthesized the hexacyclic A–F array of CTX3C with the functionality required for subsequent fragment coupling (*vide infra*).^11^ We now report the stereoselective synthesis of the tricyclic I–K fragment of CTX3C that possesses 13 stereogenic centers and has the side-chain functionality required for construction of the LM spiroacetal and attachment of the A–F array.

Our anticipated synthetic route to CTX3C is derived from the retrosynthetic analysis shown in Figure 1. Disconnection of CTX3C commences with scission of the C–O bond in ring H connected to ring G and removal of the methyl group at the GH ring junction. This key disconnection at a central position reveals alcohol **i**, which bears a vinylogous ester group in ring G (shown in purple). Opening of ring G by disconnection of the C–O bond of the enol ether leads to the γ′-hydroxy ynone **ii**, which can be disconnected to give the hexacyclic nitrile-bearing A–F array **iii** and the pentacyclic alkyne-bearing I–M array **iv**. Complete disconnection of rings L and M at the spiroacetal and hydration of ring K then reveals the tricyclic vinylogous ester **v**. Ring opening of ring K by disconnection of the C–O bond of the enol ether then leads to the bicyclic γ′-hydroxy ynone **vi**. Synthesis of the hexacyclic A–F fragment corresponding to nitrile **iii** has been accomplished by us very recently,^11^ so synthesis of the I–M array corresponding to **iv** would allow the skeleton of CTX3C to be completed. It should be noted that the I–M ring system found in CTX3C is largely conserved across the various Pacific ciguatoxins, so construction of this pentacyclic array is relevant to this entire family of natural product targets.

The synthetic route suggested by the retrosynthetic analysis shown in Scheme 1 would involve the synthesis of both seven-membered rings G and K by a formal nucleophilic 7-*endo*-*dig* cyclization of a hydroxyl group onto the alkyne of an ynone to give a vinylogous ester. Although base and acid promoted reactions of this type have been used to prepare medium-sized cyclic ethers with varying degrees of success,^12,13^ we wished to explore a mild metal-catalyzed alternative that would be compatible with highly functionalized intermediates that possess acid- or base-sensitive functionality, or labile protecting groups. Particularly appealing in this regard was a gold-catalyzed reaction that has been used by Uchiyama and co-workers to prepare related cyclic ethers (Scheme 1).^14^ These workers reported a single example of the use of the reaction for the cyclization of a simple γ′-hydroxy ynone. In this case, the reaction of ynone substrate **1** with a gold(I) complex resulted in formation of the bicyclic ether **2** in a reasonable yield (Scheme 1). We intended to use this reaction during our synthesis of CTX3C to effect closure of fragments **vii** and **ix** to construct rings K and G and deliver polyether arrays **viii** and **x** (Scheme 1). In the case of ring K, the reaction was expected to be significantly more challenging than in the simple model reaction reported by Uchiyama and co-workers because the hydroxyl group would be required to undergo nucleophilic attack at a sterically congested position due to chain branching α and β to the alkyne, which could result in preferential attack on the alkyne adjacent to the ketone instead of at the remote position.

**Scheme 1:**
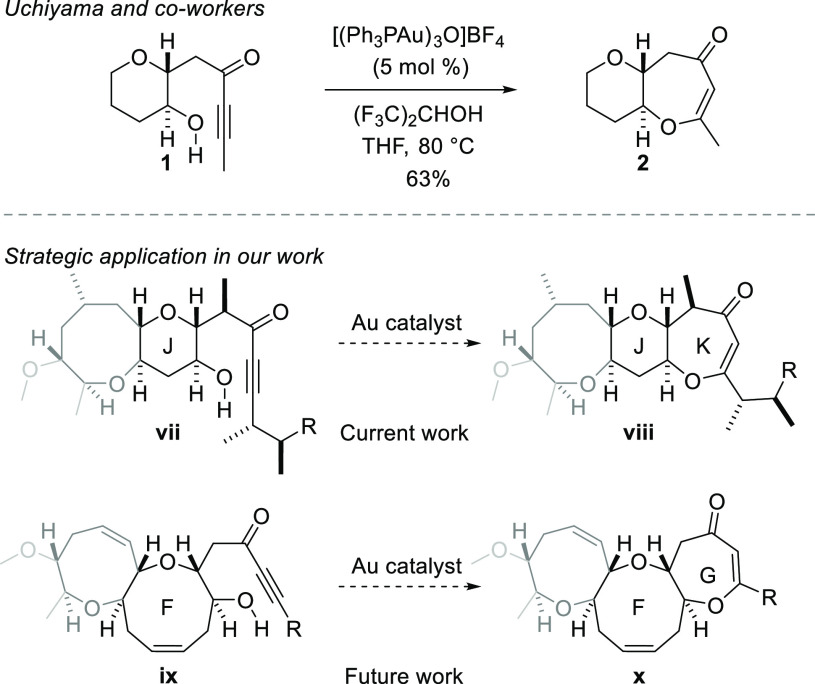
Gold-Catalyzed Formation of a Fused Seven-Membered Cyclic Ether

Synthesis of the I–K array commenced with alcohol **3** (Scheme 2), which was prepared from d-glucal in five steps as described by us previously.^10b,15^ Alkylation of the alcohol with 1-chloro-3-(triphenylphosphoranylidene)-2-propanone and reaction of the resulting phosphonium ylide with buffered aqueous formaldehyde produced the enone **4** in 68% over two steps.^16^ Direct formation of ring I by ring-closing metathesis (RCM) delivered the tricyclic enone **6** in low yield, so the enone **4** was subjected to Luche reduction to give a diastereomeric mixture of the allylic alcohols **5** prior to metathesis. RCM of diene **5**, mediated by the Hoveyda–Grubbs catalyst, followed by oxidation, delivered tricyclic enone **6** in 50% yield over two steps. The I-ring side chain was then installed by use of a stereoselective Tsuji–Trost allylation reaction.^17^ Enone **6** was first deprotonated by treatment with sodium bis(trimethysilyl)amide and *O*-acylated with allyl chloroformate to give enol carbonate **7**. Stereoselective allylation was then accomplished by treatment of the enol carbonate with the complex generated from palladium tetrakis(triphenylphosphine) and the (*S*)-*t*-butyl-PHOX ligand (**8**). The reaction delivered the allylated product in excellent yield and with the excellent level of diastereocontrol (dr 14:1) expected on the basis on our previous results.^17^ Installation of the I-ring methyl substituent (at C36) was then performed by conjugate addition of dimethylcopper lithium to the enone at low temperature.^18^ The ketone **9** was obtained as a single diastereomer, and subsequent reduction with diisobutylaluminum hydride was also high-yielding and diastereoselective. The resulting alcohol (**10**) was a crystalline solid, and its structure was established fully by X-ray crystallography. The secondary hydroxyl group was silylated with *t*-butyldimethylsilyl trifluoromethanesulfonate, and the acetonide was cleaved to afford the 1,3-diol **11** (Scheme 2).

**Scheme 2:**
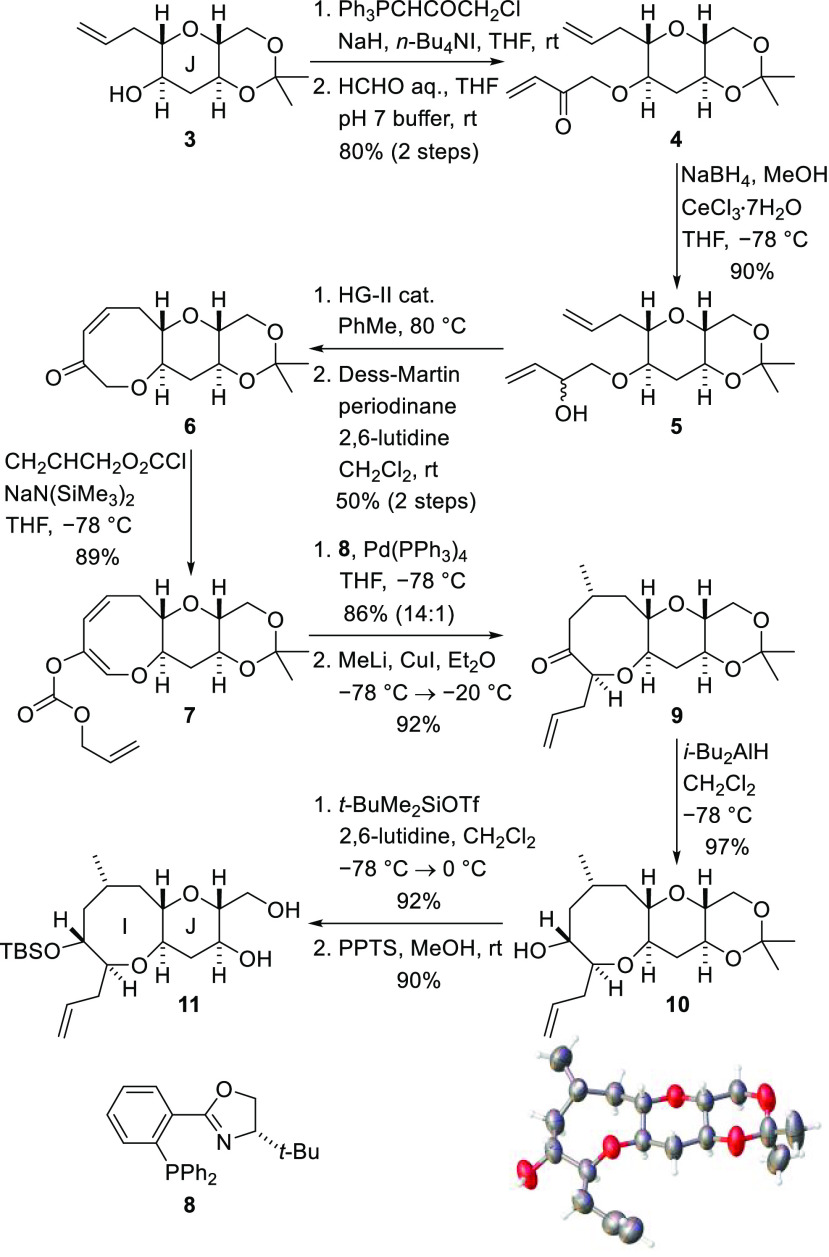
Synthesis of the IJ Fragment

The diol **11** was functionalized to enable construction of ring K as shown in Scheme 3. The primary hydroxyl group was converted into a triflate, and treatment of this compound with tetra-*n*-butylammonium cyanide produced nitrile **12**. Nitrile reduction with diisobutylaluminum hydride afforded the aldehyde **13**, and subsequent Pinnick oxidation delivered the corresponding carboxylic acid. Yamaguchi lactonization of the γ-hydroxy acid then afforded the fused lactone **14** in good yield.^20^

**Scheme 3:**
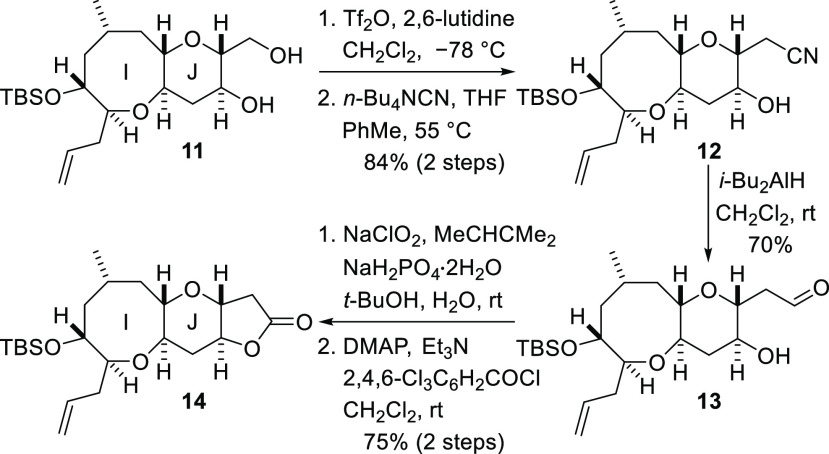
Chain Extension for Ring K

Functionalization of both the lactone and the I-ring side chain was now required. Methylation of lactone **14** was accomplished by deprotonation with lithium bis(trimethylsilyl)amide at low temperature and alkylation of the resulting enolate with methyl iodide (Scheme 4). Although the methylation reaction was high-yielding, it did not deliver the required diastereomer as the major product. This stereochemical issue was addressed by sequential deprotonation of the methylated lactone with LDA and aqueous quench of the enolate to give the diastereomeric lactone **15**.^21^ The allyl side chain of ring I was then subjected to one-pot dihydroxylation and periodate cleavage to generate aldehyde **16**. Reduction of the aldehyde with sodium borohydride and protection of the resulting primary alcohol as a *t*-butydiphenylsilyl ether delivered tricyclic lactone **17**. Reductive opening of the lactone and double protection of the resulting diol by treatment with triethylsilyl chloride produced differentially protected bicyclic fragment **18**. Direct Swern oxidation of this intermediate resulted in selective cleavage of the primary triethylsilylether with concomitant oxidation and delivered the aldehyde **19** ready for addition of the chain required to complete ring L (Scheme 4).

**Scheme 4:**
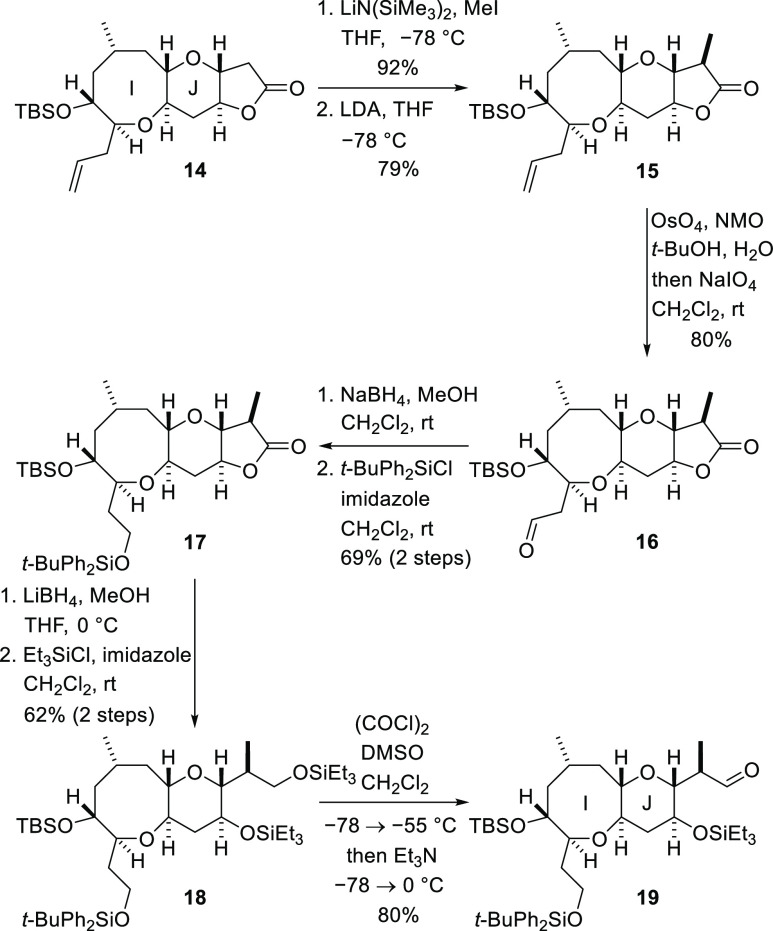
Introduction of a K-Ring Methyl Substituent and Side Chain Functionalization

The side-chain fragment required for construction of rings K and L was prepared from enantiomerically pure (2*S*,3*S*)-2,3-dimethyl-1,4-butanediol (**20**).^22^ This known diol was prepared by oxidative homocoupling of the enolate of (4*R*)-isopropyl-3-propionyl-2-oxazolidinone, according to the procedure used by Lu and Zakarian to prepare the antipode, followed by reduction with lithium borohydride (Scheme 5).^23^ The C_2_-symmetric diol **20** was first monoprotected as a *t*-butyldiphenylsilyl ether to give the alcohol **21**, and then the remaining free hydroxyl group was subjected to Dess–Martin oxidation to produce the aldehyde **22**. The aldehyde was converted into the terminal alkyne **23** by use of the Ohira–Bestmann variant of the Seyferth–Gilbert homologation reaction.^24^ Cleavage of the silyl ether and reprotection of the hydroxyl group as a pivaloyl ester afforded the ester **24**, and treatment of this terminal alkyne with iodine in the presence of morpholine delivered the alkynyl iodide **25** in excellent yield.^25^

**Scheme 5:**
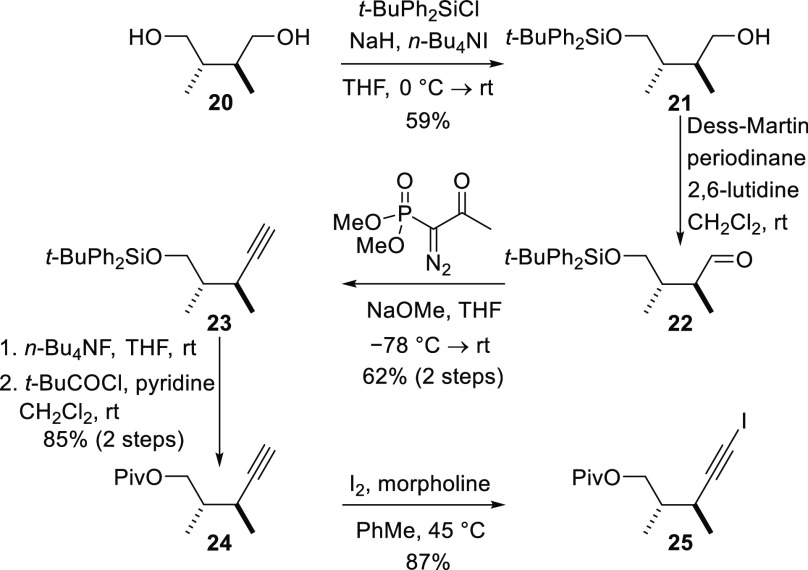
Synthesis of the L-Ring Fragment

The synthesis of the complete I–K fragment was accomplished, as shown in Scheme 6. The aldehyde **19** and the iodide **25** were coupled in good yield by use of a Nozaki–Hiyama–Kishi reaction,^26^ and the resulting diastereomeric mixture of propargylic alcohols was subjected to immediate Dess–Martin oxidation to produce the ketone **26** (Scheme 6). Cleavage of the TES ether under acidic conditions then set the stage for the key gold-catalyzed reaction to form ring K. Treatment of the alcohol with tris[(triphenylphosphine)gold]oxonium tetrafluoroborate (10 mol %) in the presence of hexafluoroisopropanol, according to the procedure described by Uchiyama and co-workers for the cyclization of alkyne **1** to give the enol ether **2** (Scheme 1),^14^ resulted in the anticipated 7-*endo*-*dig* cyclization reaction to deliver the vinylogous ester **27** in 73% yield. Hydrogenation of the alkene and regioselective conversion of the resulting saturated ketone into an enol triflate delivered the tricyclic ether **28**.^21^ The enol triflate was reduced to the corresponding alkene by reaction with the palladium hydride reagent generated by treatment of dichlorobis(triphenylphosphine)palladium(II) with formic acid.^27^ The alkene was then subjected to stereoselective Sharpless dihydroxylation mediated by AD-mix-ß.^28^ Thus, the eight-step sequence shown in Scheme 6 delivered the complete IJK fragment **29**.^21^

**Scheme 6:**
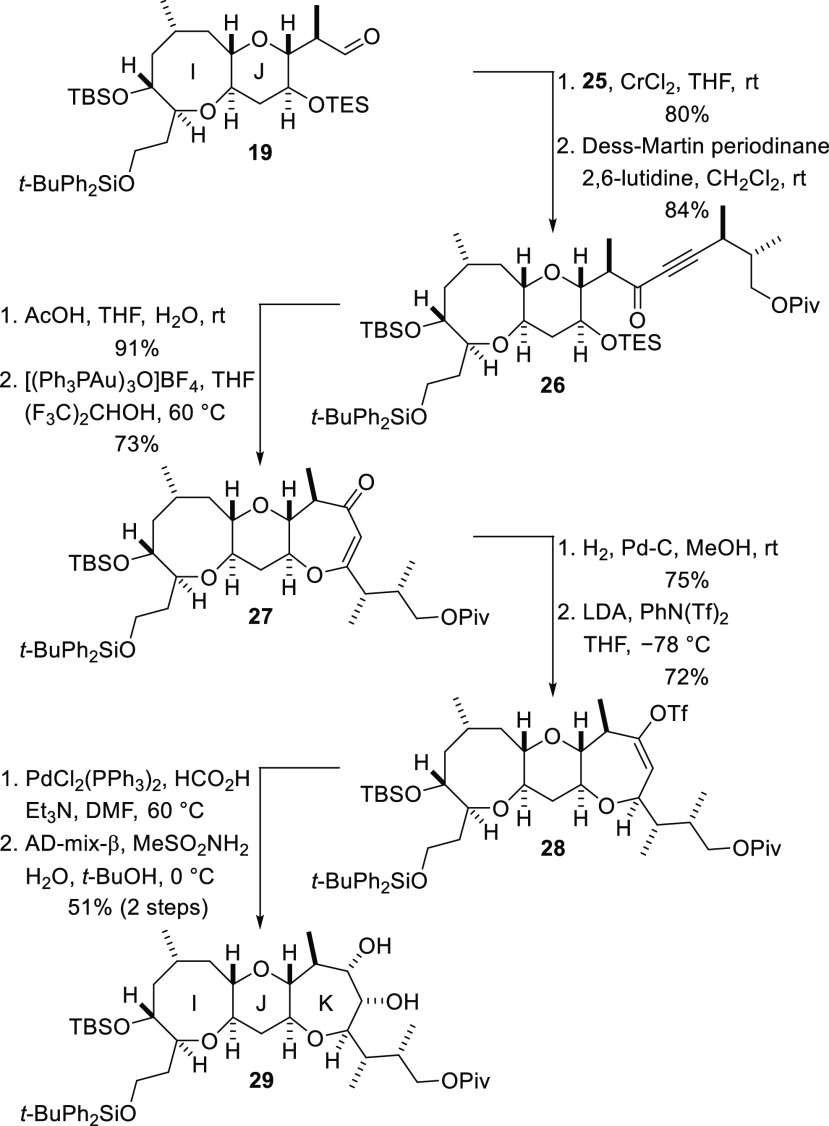
Completion of the Fully Functionalized I–K Polyether Array of CTX3C

In summary, tricyclic diol **29** that corresponds to the I–K fragment of CTX3C (C31–C49) and related ciguatoxins has been synthesized from secondary alcohol **3**, which is readily available from the chiral pool. The tricyclic fragment contains the dimethyl substituted side chain required for formation of ring L. Functionalization of ring I was achieved in a highly stereoselective manner by use of a Tsuji–Trost allylation reaction, a transformation that we have previously employed for the efficient elaboration of other medium-sized cyclic ethers. Formation of the seven-membered ring K was accomplished by gold-catalyzed intramolecular nucleophilic addition of a hydroxyl group onto a propargylic ketone. This reaction not only serves to construct ring K but also establishes the viability of our anticipated approach for formation of ring G after union of the A–F and I–M arrays in our planned synthesis of CTX3C.

## Data Availability

The data underlying this study are available in the published article and its Supporting Information.

## Supporting Information Available

Experimental procedures for the preparation of all new compounds along with characterization data and copies of ^1^H and ^13^C NMR spectra (PDF)
