# Supplementary material for: Synthesis of the I–K Fused Polyether Array of CTX3C and Related Ciguatoxins by Use of a Gold-Catalyzed Cyclization Reaction
Source: Org Lett. 2024 Jan 18;26(4):775–80. doi: 10.1021/acs.orglett.3c03782 (PMC10845158; doi:10.1021/acs.orglett.3c03782)
Supplement: Supplementary file 1 — ol3c03782_si_001.pdf [file ol3c03782_si_001.pdf]

Synthesis of the I–K Fused Polyether Array of CTX3C and Related Ciguatoxins by Use of a Gold-Catalyzed Cyclization Reaction

Venkaiah Chintalapudi, Claire Wilson and J. Stephen Clark*

School of Chemistry, Joseph Black Building, University of Glasgow, University Avenue, Glasgow G12 8QQ, United Kingdom

stephen.clark@glasgow.ac.uk

Supporting Information

Table of Contents

General Information	SI-1
Experimental Procedures for the Preparation of New Compounds	SI-2
References	SI-23
^{1}H and ^{13}C NMR Spectra of New Compounds	SI-24
{^{1}H–^{1}H} gNOESY NMR Spectra for Compounds **S3**, **15**, **S6**, **28** and **29**	SI-55
X-Ray Crystal Data, Data Collection and Refinement Information for **10**	SI-60

General Information

Air- and moisture-sensitive reactions were performed under an atmosphere of argon in flame dried apparatus. Tetrahydrofuran (THF), toluene, dichloromethane and diethyl ether were dried and purified using a Pure-SolvTM 500 Solvent Purification System. Other organic solvents and starting materials were obtained from commercial sources and used as received unless otherwise specified. Petroleum ether used for column chromatography was the 40−60 °C fraction.

Reactions were monitored by thin layer chromatography (TLC) using Merck silica gel 60 F_{254} aluminium plates. TLC plates were visualised under UV light and stained using either potassium permanganate solution or acidic ethanolic anisaldehyde solution or phosphomolybdic acid solution.

Flash column chromatography was performed on silica gel (Fluorochem LC60A 35–70 μm, or Geduran Si 60 35−70 μm).

IR spectra were recorded using a Shimadzu FT IR-8400S ATR instrument. The IR spectrum of each compound (solid or liquid) was acquired directly on a thin layer at ambient temperature.

^{1}H NMR spectra were recorded on Bruker AVIII 400 MHz and 500 MHz spectrometers at ambient temperature. ^{13}C NMR spectra were recorded on a Bruker AVIII 400 MHz and 500 MHz spectrometers at 101 MHz and 126 MHz at ambient temperature. Structural and stereochemical assignments were made with additional information from gNOESY experiments.

High resolution mass spectra (HRMS) were recorded using positive chemical ionization (CI+), positive ion impact (EI+) ionisation or fast atom bombardment (FAB) on a Jeol MStation JMS-700 instrument or by using positive or negative ion electrospray (ESI+/ESI–) techniques on a Bruker 2 micrOTOF-Q instrument.

Optical rotations were recorded with using an Autopol IV or Autopol V automatic polarimeter.

Melting points were recorded with an Electrothermal IA 9100 apparatus.

Enone 4.

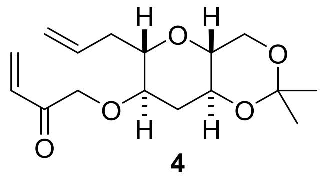

4

Sodium hydride (60% by weight, 4.0 g, 0.10 mol) was washed with pentane (3 × 50 mL) and suspended in THF (400 mL). A solution of the alcohol **3** (9.12 g, 39.9 mmol) in THF (400 mL) was added and the resultant suspension was stirred for 5 min.[1] 1-Chloro-3-(triphenylphosphoranylidene)-2-propanone (20.7 g, 58.7 mmol) was added followed by tetra-*n*-butylammonium iodide (1.48 g, 4.0 mmol) and the mixture was heated to reflux (oil bath) and stirred for 6 h. The reaction was quenched by the cautious addition of water (500 mL). The mixture was extracted with ethyl acetate (3 × 200 mL) and the combined organic extracts were dried ($MgSO_4$) and concentrated under reduced pressure to afford the crude ylide as a brown foam. The crude residue was used directly in the next reaction without purification.

To a solution of the crude ylide in a mixture of THF (700 mL) was added pH 7 buffer solution (700 mL) and formaldehyde (65 mL of a 37% w/w solution in water, 0.80 mmol). The mixture was stirred vigorously at room temperature for 2 h and then diluted with ethyl acetate (500 mL). The phases were separated and the organic phase was washed with water (3 × 200 mL) and then dried (magnesium sulfate). The crude products and adsorbed onto Celite and then dry loaded onto a column of silica gel. Purification by flash column chromatography on silica gel (diethyl ether-pet. ether, 3:17 → 3:7) afforded the enone **4** (9.5 g, 80% over two steps) as a colourless oil. R_f = 0.55 (diethyl ether-pet. ether, 3:2). $[\alpha]_D^{25}$ −41 (*c* = 0.5, $CHCl_3$). ν_{max} (film) 2996, 2954, 2890, 1712, 1687, 1643, 1615, 958, 904, 849, 753 cm^{-1}. ^1H NMR (400 MHz, $CDCl_3$) δ 6.55 (1H, dd, *J* = 17.6, 10.6 Hz), 6.34 (1H, dd, *J* = 17.6, 1.4 Hz), 5.88 (1H, dddd, *J* = 17.3, 10.2, 7.5, 6.3 Hz), 5.84 (1H, dd, *J* = 10.7, 1.4 Hz), 5.13–5.04 (2H, m), 4.33 (1H, d, *J* = 16.5 Hz), 4.22 (1H, d, *J* = 16.5 Hz), 3.89 (1H, dd, *J* = 10.8, 5.2 Hz), 3.66 (1H, dd, *J* = 10.8, 10.4 Hz), 3.50 (1H, ddd, *J* = 11.5, 9.2, 4.1 Hz), 3.39 (1H, ddd, *J* = 9.1, 7.3, 3.2 Hz), 3.28 (1H, ddd, *J* = 10.8, 9.1, 4.5 Hz), 3.18 (1H, ddd, *J* = 10.4, 9.2, 5.2 Hz), 2.65 (1H, dddt, *J* = 14.8, 6.3, 3.2, 1.4 Hz), 2.41 (1H, ddd, *J* = 11.3, 4.5, 4.1 Hz), 2.28 (1H, dddt, *J* = 14.8, 7.5, 7.3, 1.4), 1.50 (1H, ddd, *J* = 11.5, 11.3, 10.8 Hz), 1.47 (3H, s), 1.40 (3H, s). ^{13}C NMR (101 MHz, $CDCl_3$) δ 196.6, 134.5, 132.3, 129.4, 117.0, 99.1, 80.1, 77.3, 74.1, 72.9, 68.8, 62.7, 36.1, 34.9, 29.2, 19.1. HRMS (ESI+) *m*/*z*: $[M + Na]^+$ Calcd for $C_{16}H_{24}NaO_5$ 319.1516; Found 319.1517.

Allylic Alcohol 5.

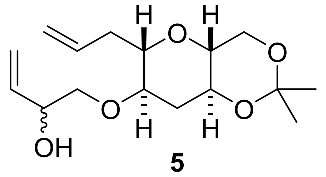

5

To a solution of the enone **4** (8.0 g, 27 mmol) in and mixture of methanol and dichloromethane (1:1, 1000 mL) was added cerium(II) chloride heptahydrate (20.0 g, 53.7 mmol). The resultant solution was cooled to −78 °C and sodium borohydride (2.0 g, 53 mmol) was added. The reaction mixture was stirred for 2 h and then diluted with ethyl acetate (1.0 L). The mixture was washed with a saturated aqueous solution of ammonium chloride (3 × 500 mL), dried (magnesium sulfate) and concentrated under reduced pressure to afford a diastereomeric mixture of the allylic alcohols **5** (7.25 g, 90%, *dr*:1:1) as a colourless oil. The crude product was used directly in the next reaction. R_f = 0.38 (diethyl ether-pet. ether, 4:1). ν_{max} (film) 3463, 2992, 2941, 2877, 1642, 920, 854, 753 cm^{-1}. ^1H NMR (400 MHz, $CDCl_3$) δ 5.92–5.80 (1H, m), 5.82 (1H, ddd, *J* = 17.3, 10.6, 5.6 Hz) 5.34 (1H, app. dq, *J* = 17.3, 1.4 Hz), 5.20 (1H, app. dt, *J* = 10.6, 1.5 Hz), 5.13–5.03 (2H, m), 4.30–4.22 (1H, m), 3.88 (1H, dd, *J* = 10.8, 5.2 Hz), 3.66 (1H, dd, *J* = 10.8 Hz, 10.4 Hz), 3.55–3.37 (2H, m), 3.36–3.29 (1H, m), 3.27–3.19 (2H, m), 3.19–3.12 (1H, m), 2.60–2.51 (1H, m), 2.45–2.38 (1H, m), 2.35–2.21 (2H, m), 1.47 (3H, s), 1.44 (1H, dd, *J* = 10.9, 3.3 Hz), 1.39 (3H, s). ^{13}C NMR (101 MHz, $CDCl_3$) δ 136.6, 136.4, 134.7, 134.6, 116.9 (2C), 116.6 (2C), 99.1 (2C), 80.0 (2C), 77.4, 76.7 (2C), 74.2, 72.8, 72.6, 71.8, 71.5, 68.9, 68.9, 62.7, 36.4, 36.3, 35.2, 35.0, 29.2, 19.1. HRMS (ESI+) *m*/*z*: $[M + Na]^+$ Calcd for $C_{16}H_{26}NaO_5$ 321.1672; Found 321.1672.

Enone 6.

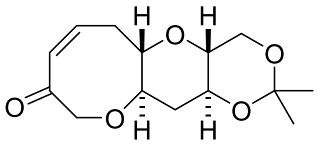

6

To a solution of the alcohols **5** (2.0 g, 6.71 mmol) in toluene (2.0 L) was added the Hoveyda-Grubbs II catalyst (419 mg, 0.67 mmol). The resultant solution was heated to 60 °C (oil bath) and stirred at that temperature for 16 h, then concentrated under reduced pressure. The residue was dissolved in dichloromethane (330 mL) and 2,6-lutidine (1.94 mL, 16.7 mmol) and Dess-Martin periodinane (4.26 g, 10.0 mmol) were added. The reaction mixture was stirred for 16 h, diluted with a mixture of saturated aqueous sodium thiosulfate and saturated aqueous sodium bicarbonate (1:1, 500 mL) and then stirred vigorously for 1 h. The mixture was diluted with diethyl ether (1.0 L) and the phases were separated. The organic phase was washed with a saturated aqueous solution of sodium bicarbonate (2 × 300 mL), dried (magnesium sulfate) and concentrated under reduced pressure. The crude residue was purified by flash column chromatography on silica gel (diethyl ether-pet. ether, 1:4 → 3:2) to afford the bicyclic enone **6** (900 mg, 50%) as a colourless solid. R_f = 0.78 (diethyl ether-pet. ether, 4:1). Mp 120–121 °C. $[\alpha]_D^{25}$ −117 (*c* = 0.20, $CHCl_3$). ν_{max} (film) 2997, 2954, 2883, 1678, 959, 853, 759 cm^{-1}. ^1H NMR (400 MHz, $CDCl_3$) δ 6.45 (1H, ddd, *J* = 12.3, 8.8, 7.6 Hz), 5.87 (1H, br d, *J* = 12.3 Hz), 4.52 (1H, dd, *J* = 17.8, 0.8 Hz), 4.21 (1H, d, *J* = 17.8 Hz), 3.88 (1H, dd, *J* = 10.8, 5.2 Hz), 3.67 (1H, dd, *J* = 10.8, 10.4 Hz), 3.61 (1H, ddd, *J* = 11.4, 9.4, 4.2

Hz), 3.49 (1H, ddd, J = 11.4, 9.2, 4.2 Hz), 3.36 (1H, app. td, J = 9.4, 1.4 Hz), 3.18 (1H, ddd, J = 10.4, 9.2, 5.2 Hz), 2.67 (1H, dddd, J = 15.2, 9.4, 7.6, 1.7 Hz), 2.54 (1H, ddd, J = 15.2, 8.8, 1.4 Hz), 2.27 (1H, dt, J = 11.6, 4.2 Hz), 1.72 (1H, ddd, J = 11.6, 11.4, 11.4 Hz), 1.49 (3H, s), 1.41 (3H, s). ^{13}C NMR (101 MHz, $CDCl_3$) δ 203.5, 137.7, 129.1, 99.4, 85.3, 78.7, 77.8, 74.9, 69.4, 62.8, 36.5, 35.0, 29.3, 19.2. HRMS (ESI+) *m*/*z*: $[M + Na]^+$ Calcd for $C_{14}H_{20}NaO_5$ 291.1203; Found 291.1202.

Enol Carbonate 7.

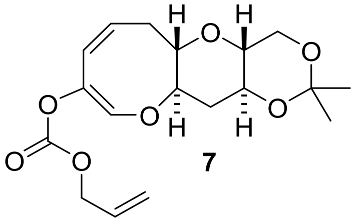

7

To a solution of the enone **6** (1.70 g, 6.34 mmol) and allyl chloroformate (3.36 mL, 31.6 mmol) in THF (62 mL), was added sodium bis(trimethylsilyl)amide (12.7 mL of a 1.0 M solution in THF, 12.7 mmol) at −78 °C. The resultant suspension was stirred at −78 °C for 2 h and the reaction was quenched by the addition of a saturated aqueous solution of ammonium chloride (50 mL). The mixture was diluted with diethyl ether (50 mL), washed with brine, dried (magnesium sulfate) and concentrated under reduced pressure. The residue was purified by flash column chromatography on silica gel (pet. ether-diethyl ether, 5:1) to afford the enol carbonate **7** (2.0 g, 89%) as a colourless oil. R_f = 0.38 (ethyl acetate-pet. ether, 3:17). $[\alpha]_D^{25}$ +58 (*c* = 0.25, $CHCl_3$). ν_{max} (film) 2952, 2879, 1756, 1678, 1656, 978, 855, 784, 751 cm^{-1}. ^1H NMR (400 MHz, $CDCl_3$) δ 6.58 (1H, d, J = 0.7 Hz), 5.94 (1H, ddt, J = 17.2, 10.4, 5.8 Hz), 5.96–5.92 (1H, m), 5.74 (1H, ddd, J = 10.8, 8.6, 7.2 Hz), 5.38 (1H, app. dq, J = 17.2, 1.4 Hz), 5.29 (1H, app. dq, J = 10.4, 1.2 Hz), 4.64 (2H, dt, J = 5.8, 1.3 Hz), 4.47 (1H, ddd, J = 11.5, 9.1, 4.4 Hz), 3.88 (1H, dd, J = 10.7, 5.1 Hz), 3.66 (1H, dd, J = 10.7, 10.4 Hz), 3.53 (1H, ddd, J = 11.4, 9.3, 4.1 Hz), 3.37 (1H, ddd, J = 9.1, 3.9, 2.9 Hz), 3.20 (1H, ddd, J = 10.4, 9.3, 5.1 Hz), 2.84 (1H, dddd, J = 14.1, 8.6, 3.9, 1.5 Hz), 2.55 (1H, ddd, J = 14.1, 7.2, 2.9 Hz), 2.26 (1H, ddd, J = 11.2, 4.4, 4.1 Hz), 1.67 (1H, ddd, J = 11.5, 11.4, 11.2 Hz), 1.47 (3H, s), 1.40 (3H, s). ^{13}C NMR (101 MHz, $CDCl_3$) δ 154.7, 141.7, 131.3, 130.0, 128.0, 126.5, 119.5, 99.4, 75.1, 74.2, 73.1, 69.3, 69.1, 62.8, 36.2, 31.4, 29.3, 19.2. HRMS (ESI+) *m*/*z*: $[M + H]^+$ Calcd for $C_{18}H_{25}O_7$ 353.1595; Found 353.1592.

Enone S1.

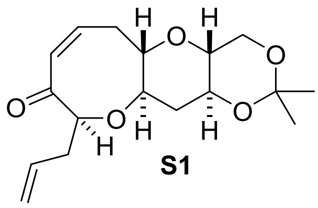

S1

To a mixture of tetrakis(triphenylphosphine)palladium(0) (590 mg, 0.511 mmol) and the ligand **8** (297 mg, 0.767 mmol) was added THF (225 mL) and the resulting solution was stirred at room temperature for 30 min. A solution of enol carbonate **7** (1.80 g, 5.11 mmol) in THF (5 mL) was added dropwise over 5 min. The reaction mixture was stirred at room temperature at rt for 14 h and then concentrated. The crude material was dry loaded onto a column of silica gel and purified by flash column chromatography on silica gel (pet. ether-diethyl ether, 5:1) to deliver the enone **S1** (1.35 g, 86%, 14:1 *dr*) as a colourless oil. R_f = 0.40 (ethyl acetate-pet. ether, 3:17). $[\alpha]_D^{25}$ −100 (*c* = 0.7, $CHCl_3$). ν_{max} (film) 2996, 2939, 2876, 1664, 911, 862, 777, 756 cm^{-1}. ^1H NMR (500 MHz, $CDCl_3$) δ 6.44 (1H, dt, *J* = 12.2, 8.8 Hz), 5.89 (1H, d, *J* = 12.2 Hz), 5.83 (1H, ddt, *J* = 17.3, 9.5, 7.4 Hz), 5.20–5.10 (2H, m), 4.19 (1H, dd, *J* = 9.4, 3.2 Hz), 3.88 (1H, dd, *J* = 10.7, 5.1 Hz), 3.66 (1H, app. t, *J* = 10.6 Hz), 3.58 (1H, ddd, *J* = 10.6, 10.2, 4.1 Hz), 3.43 (1H, td, *J* = 10.7, 9.6, 4.2 Hz), 3.28 (1H, app. t, *J* = 9.6 Hz), 3.18 (1H, td, *J* = 9.9, 5.1 Hz), 2.74–2.61 (2H, m), 2.50 (1H, dd, *J* = 13.8, 9.9 Hz), 2.36–2.24 (2H, m), 1.65 (1H, app. q, *J* = 11.4 Hz), 1.49 (3H, s), 1.41 (3H, s). ^{13}C NMR (126 MHz, $CDCl_3$) δ 202.5, 136.6, 134.0, 130.6, 118.7, 99.4, 88.6, 86.2, 78.1, 75.0, 69.4, 62.7, 37.3, 37.2, 34.1, 29.3, 19.2. HRMS (ESI+) *m/z*: $[M + H]^+$ Calcd for $C_{17}H_{25}O_5$ 309.1697; Found 309.1696.

Ketone 9.

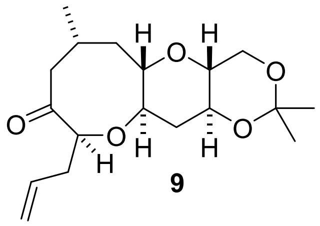

9

To a stirred solution of activated copper(I) iodide (heated under vacuum at 130 °C for 30 min.) (13.2 g, 69.3 mmol) in diethyl ether (200 mL) at −78 °C was added methyllithium (72 mL of a 1.6 M solution in diethyl ether, 0.12 mol) dropwise. This was stirred at −78 °C for 20 min and then warmed to 0 °C and stirred for 30 min. The resulting grey homogenous solution was re-cooled to −78 °C and a solution of the enone **S1** (4.73 g, 15.4 mmol) in diethyl ether (60 mL) was added dropwise over 15 min. The reaction mixture was stirred at −78 °C, then allowed to warm slowly to −20 °C and stirred at this temperature for 5 h. The reaction was quenched by the addition of an aqueous solution of ammonium chloride and ammonium hydroxide (10:1) and brine. The organic phase was dried (magnesium sulfate) and the crude product was purified by flash column chromatography on silica gel (pet. ether-diethyl ether, 5:1) to yield the desired ketone **9** (4.60 g, 92%) as a colourless oil. R_f = 0.65 (ethyl acetate-pet. ether, 13:7). $[\alpha]_D^{25}$ −133 (*c* = 0.5, $CHCl_3$). ν_{max} (film) 2939, 2888, 1709, 991, 942, 923, 857, 757 cm^{-1}. ^1H NMR (400 MHz, $CDCl_3$) δ 5.75 (1H, ddt, *J* = 17.1, 10.2, 7.0 Hz), 5.10–5.02 (2H, m), 3.84 (1H, dd, *J* = 10.7, 5.2 Hz), 3.62 (1H, app. t, *J* = 10.5 Hz), 3.61 (1H, dd, *J* = 9.3, 4.1 Hz), 3.51 (1H, ddd, *J* = 11.5, 9.3, 4.1 Hz), 3.41 (1H, dd, *J* = 11.2, 6.7 Hz), 3.33 (1H, t, *J* = 8.9 Hz), 3.15–3.02 (2H, m), 2.43–2.34 (1H, m), 2.32–2.17 (3H, m),

1.89–1.79 (2H, m), 1.62 (1H, app. q, *J* = 11.5 Hz), 1.45 (3H, s), 1.38 (3H, s), 1.40–1.28 (1H, m), 1.00 (3H, d, *J* = 6.9 Hz). ^{13}C NMR (101 MHz, $CDCl_3$) δ 215.8, 133.6, 118.1, 99.2, 89.1, 83.1, 81.4, 74.3, 69.1, 62.8, 42.1, 41.6, 38.0, 37.3, 30.5, 29.3, 22.3, 19.2. HRMS (ESI+) *m*/*z*: $[M + Na]^+$ Calcd for $C_{18}H_{28}NaO_5$ 347.1829; Found 347.1831.

Alcohol 10.

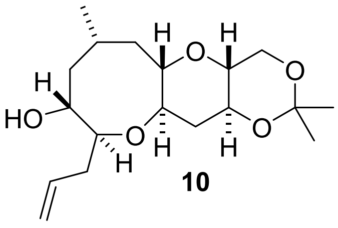

10

To the solution of ketone **9** (4.60 g, 14.2 mmol) in dichloromethane (300 mL) was cooled to −78 °C and treated with diisobutylaluminium hydride (28.3 mL of a 1.0 M solution in hexane, 28.3 mmol). The mixture was stirred at −78 °C for 2 h and then the reaction was quenched by the addition of a saturated aqueous solution of potassium sodium tartrate (300 mL). The cooling bath was removed, and the mixture was stirred vigorously at room temperature for 2 h. The resulting mixture was diluted with diethyl ether (300 mL) and washed with brine. The organic extracts were dried (magnesium sulfate), filtered and concentrated. The residue was purified by flash chromatography on silica gel (pet. ether-diethyl ether, 2:1) to afford the alcohol **10** as a white solid (4.50 g, 97% yield). R_f = 0.40 (ethyl acetate-pet. ether, 1:4). Crystals suitable for X-ray analysis were obtained by dissolving **10** in a mixture of dichloromethane and diethyl ether and slow evaporation of the solvent. Mp 81–82 °C. $[\alpha]_D^{25}$ −17 (*c* = 0.35, $CHCl_3$). ν_{max} (film) 3402, 2954, 2891, 938, 904, 855, 756 cm^{-1}. ^{1}H NMR (400 MHz, $CDCl_3$) δ 5.89 (1H, ddt, *J* = 17.2, 10.1, 7.2 Hz), 5.16–5.07 (2H, m), 3.86 (1H, dd, *J* = 10.7, 5.3 Hz), 3.63 (1H, app. t, *J* = 10.6 Hz), 3.57–3.45 (2H, m), 3.37–3.17 (3H, m), 3.11 (1H, ddd, *J* = 10.1, 9.5, 5.3 Hz), 2.61–2.52 (1H, m), 2.33 (1H, app. dt, *J* = 11.8, 4.4 Hz), 2.15 (1H, app. dt, *J* = 14.1, 8.1 Hz), 2.01–1.89 (1H, m), 1.89–1.78 (2H, m), 1.69 (1H, ddd, *J* = 14.4, 10.2, 6.7 Hz), 1.60–1.47 (2H, m), 1.48 (3H, s), 1.41 (3H, s), 1.05 (3H, d, *J* = 7.1 Hz). ^{13}C NMR (101 MHz, $CDCl_3$) δ 135.5, 117.7, 99.2, 88.4, 82.6, 81.0, 75.0, 73.8, 69.1, 62.9, 47.4, 45.2, 39.2, 37.9, 29.4, 28.0, 27.8, 19.2. HRMS (ESI+) *m*/*z*: $[M + Na]^+$ Calcd for $C_{18}H_{30}NaO_5$ 349.1985; Found 349.1990.

Silyl Ether S2.

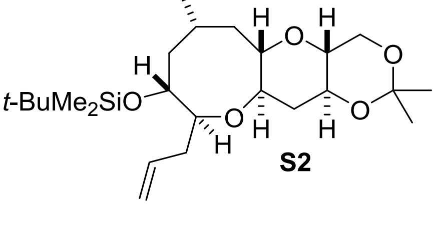

S2

To a stirred solution of alcohol **8** (4.60 g, 14.1 mmol) and 2,6-lutidine (4.9 mL, 42.2 mmol) in dry dichloromethane (200 mL) at −78 °C under an argon atmosphere, was added *t*-butyldimethylsilyl trifluoromethanesulfonate (4.85 mL, 21.1 mmol) dropwise over 10 min. The reaction mixture was stirred at −78 °C for 30 min and was then allowed warm to 0 °C and stirred for a further 1 h. The reaction was quenched by the addition of a saturated aqueous solution of sodium bicarbonate (200 mL) and the mixture was extracted with diethyl ether (3 × 200 mL). The combined organic extracts were dried over (magnesium sulfate) and the residue was purified by flash column chromatography on silica gel (pet. ether-ethyl acetate, 20:1 → 7:3) to give the silyl ether **S2** as a colourless oil (5.70 g, 92%). R_f = 0.50 (pet. ether-diethyl ether, 17:3). $[\alpha]_D^{25}$ −29 (*c* = 0.6, $CHCl_3$). ν_{max} (film) 2953, 2929, 2858, 938, 834, 772 cm^{-1}. ^1H NMR (400 MHz, $CDCl_3$) δ 5.91–5.79 (1H, m), 5.13–5.03 (2H, m), 3.86 (1H, dd, *J* = 10.7, 5.3 Hz), 3.63 (1H, app. t, *J* = 10.6 Hz), 3.51–3.42 (2H, m), 3.37 (1H, ddd, *J* = 10.4, 9.6, 5.0 Hz), 3.32–3.21 (2H, m), 3.11 (1H, ddd, *J* = 10.1, 9.6, 5.2 Hz), 2.57–2.48 (1H, m), 2.32 (1H, app. dt, *J* = 11.7, 4.4 Hz), 2.04–1.87 (2H, m), 1.87–1.80 (1H, m), 1.77 (1H, app. dt, *J* = 14.8, 2.8 Hz), 1.72–1.51 (3H, m), 1.48 (3H, s), 1.41 (3H, s), 1.02 (3H, d, *J* = 7.2 Hz), 0.88 (9H, s), 0.06 (3H, s), 0.04 (3H, s). ^{13}C NMR (101 MHz, $CDCl_3$) δ 136.1, 117.4, 99.2, 88.9, 82.0, 81.0, 75.8, 73.8, 69.2, 62.9, 46.7, 44.9, 39.1, 37.9, 29.4, 28.0, 26.0, 19.2, 18.1, −3.9, −4.6. HRMS (ESI+) *m*/*z*: $[M + Na]^+$ Calcd for $C_{24}H_{44}NaO_5Si$ 463.2850; Found 463.2869.

Diol 11.

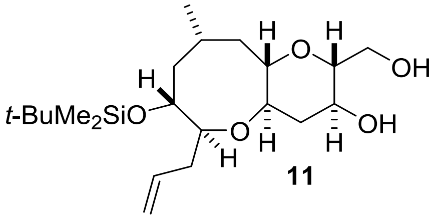

11

To a solution of the acetonide **S2** (5.45 g, 12.4 mmol) in methanol (290 mL) was added pyridinium *p*-toluenesulfonate (78.0 mg, 0.310 mmol). The reaction mixture was stirred at room temperature for 24 h. The reaction was quenched with by the addition of a saturated aqueous solution of sodium bicarbonate (300 mL), and methanol was removed under reduced pressure. The remaining aqueous mixture was extracted with ethyl acetate (3 × 200 mL). The combined organic extracts were dried (magnesium sulfate) and the solvent was removed under reduced pressure. The residue was purified by flash column chromatography on silica gel (pet. ether-ethyl acetate, 1:1) to give the diol **11** (4.40 g, 90%) as a colourless oil. R_f = 0.33 (pet. ether-ethyl acetate, 3:2). $[\alpha]_D^{25}$ −49 (*c* = 0.5, $CHCl_3$). ν_{max} (film) 3367, 2952, 2928, 2857, 911, 865, 833, 773 cm^{-1}. ^1H NMR (400 MHz, $CDCl_3$) δ 5.85 (1H, ddt, *J* = 16.6, 10.5, 7.2 Hz), 5.15–5.01 (2H, m), 3.87–3.78 (1H, m), 3.77–3.68 (1H, m), 3.66–3.51 (1H, m), 3.48 (1H, td, *J* = 8.7, 3.0 Hz), 3.37–3.23 (2H, m), 3.22–3.10 (2H, m), 2.57–2.48 (1H, m), 2.44 (1H, app. dt, *J* = 12.0, 4.6 Hz), 2.07 (2H, brs), 2.04–1.88 (1H, m), 1.87–1.75 (2H, m), 1.69–1.55 (2H, m), 1.40 (1H, app. q, *J* = 11.5 Hz), 1.03 (3H, d, *J* = 7.1 Hz), 0.88 (9H, s), 0.06 (3H, s), 0.04 (3H, s). ^{13}C NMR (101 MHz, $CDCl_3$) δ 136.1, 117.3, 88.4, 81.3, 80.7, 80.6,

75.7, 67.2, 63.4, 46.1, 44.4, 41.0, 39.1, 27.9, 27.5, 26.0, 18.1, −3.9, −4.6. HRMS (ESI+) *m*/*z*: $[M + Na]^+$ Calcd for $C_{21}H_{40}NaO_5Si$ 423.2537; Found 423.2544.

Nitrile 12.

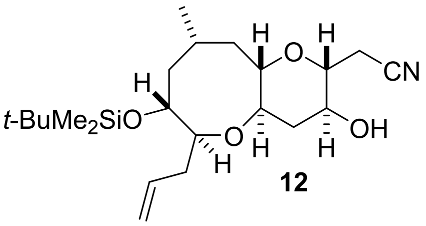

12

To a solution of the diol **11** (4.30 g, 10.7 mmol) and 2,6-lutidine (6.20 mL, 53.5 mmol) in dichloromethane (80 mL) cooled to −78 °C was added dropwise trifluoromethanesulfonic anhydride (1.90 ml, 11.3 mmol). The resulting mixture was stirred at −78 °C for 1 h and then diluted with diethyl ether (100 mL). The mixture was washed successively with a saturated aqueous solution of sodium bicarbonate (100 mL), a saturated aqueous solution of copper(II) sulfate and brine. The organic phase was dried (magnesium sulfate) and concentrated to give an oil. The residue was filtered through short pad of silica gel to afford the monotriflate as a brown oil, which was used in next step without purification.

To the solution of above triflate (approximately 10.8 mmol) in a mixture of THF (440 mL) and toluene (44 mL) was added tetra-*n*-butylammonium cyanide (14.5 g, 54.0 mmol) and the resulting mixture was heated to 55 °C (oil bath) and strirred at that temperature for 16 h. The mixture was cooled to room temperature and then poured into water (500 mL). The mixture was extracted with diethyl ether (3 × 500 mL) and the combined organic extracts were washed with brine, dried (magnesium sulfate) and concentrated. The residue was purified by flash chromatography on silica gel (pet. ether-ethyl acetate, 5:1) to afford the nitrile **12** as a pale yellow oil (3.70 g, 84% over two steps). R_f = 0.77 (pet. ether-ethyl acetate, 7:3). $[\alpha]_D^{25}$ −15 (*c* = 0.25, $CHCl_3$). ν_{max} (film) 3417, 2953, 2928, 2858, 2362, 913, 891, 853, 774 cm^{-1}. ^1H NMR (400 MHz, $CDCl_3$) δ 5.93–5.77 (1H, m), 5.13–5.03 (2H, m), 3.52–3.38 (2H, m), 3.38–3.29 (2H, m), 3.28–3.14 (2H, m), 2.78 (1H, dd, *J* = 16.8, 3.5 Hz), 2.59 (1H, dd, *J* = 16.8, 6.6 Hz), 2.57–2.49 (1H, m), 2.46 (1H, dt, *J* = 12.1, 4.6 Hz), 2.03–1.84 (3H, m), 1.80 (1H, app. dt, *J* = 14.8, 2.9 Hz), 1.68–1.57 (2H, m), 1.39 (1H, app. q, *J* = 11.5 Hz), 1.04 (3H, d, *J* = 7.1 Hz), 0.88 (9H, s), 0.07 (3H, s), 0.04 (3H, s). ^{13}C NMR (101 MHz, $CDCl_3$) δ 136.1, 117.6, 117.3, 88.4, 81.1, 80.8, 76.6, 75.7, 68.9, 46.0, 44.0, 41.3, 39.1, 27.9, 27.5, 26.0, 21.2, 18.1, −3.9, −4.6. HRMS (ESI+) *m*/*z*: $[M + Na]^+$ Calcd for $C_{22}H_{39}NNaO_4Si$ 432.2541; Found 432.2538.

Aldehyde 13.

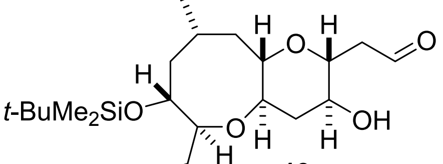

13

To a solution of nitrile **12** (1.45 g, 3.55 mmol) in dichloromethane (60 mL) was added diisobutylaluminium hydride (16 mL of a 1.0 M solution in hexane, 16 mmol) at −78 °C. The mixture was stirred at this temperature for 30 min and then the reaction was quenched by the addition of a saturated aqueous solution of potassium sodium tartrate (60 mL). The cooling bath was removed and the mixture was stirred vigorously at room temperature for 2 h. The mixture was diluted with ethyl acetate (100 mL) and then washed with brine, dried ($MgSO_4$), and concentrated. The residue was purified by flash chromatography on silica gel (pet. ether-ethyl acetate, 5:1) to afford the aldehyde as a colourless oil **13** (1.02 g, 70%). R_f = 0.75 (pet. ether-ethyl acetate, 7:3). $[\alpha]_D^{25}$ −8.6 (*c* = 0.25, $CHCl_3$). ν_{max} (film) 3387, 2953, 2929, 2858, 1726, 1641, 911, 834, 773, 734 cm^{-1}. ^1H NMR (400 MHz, $CDCl_3$) δ 9.77(1H, t, *J* = 2.2 Hz), 5.92–5.77 (1H, m), 5.12–5.03 (2H, m), 3.54 (1H, ddd, *J* = 9.0, 7.9, 4.3 Hz), 3.51–3.42 (1H, m), 3.36–3.22 (3H, m), 3.17 (1H, ddd, *J* = 10.3, 9.5, 3.4 Hz), 2.80 (1H, ddd, *J* = 16.3, 4.2, 1.8 Hz), 2.58–2.48 (2H, m), 2.45 (1H, app. dt, *J* = 12.1, 4.6 Hz), 2.03–1.85 (2H, m), 1.85–1.71 (2H, m), 1.68–1.49 (2H, m), 1.39 (1H, app. q, *J* = 11.4 Hz), 1.02 (3H, d, *J* = 7.1 Hz), 0.87 (9H, s), 0.05 (3H, s), 0.03 (3H, s). ^{13}C NMR (101 MHz, $CDCl_3$) δ 201.6, 136.1, 117.3, 88.4, 81.3, 80.9, 77.7, 75.7, 69.7, 46.5, 46.1, 44.3, 41.5, 39.1, 27.9, 27.5, 26.0, 18.0, −3.9, −4.7. HRMS (ESI+) *m/z*: $[M + Na]^+$ Calcd for $C_{22}H_{40}NaO_5Si$ 435.2537; Found 435.2532.

Lactone 14.

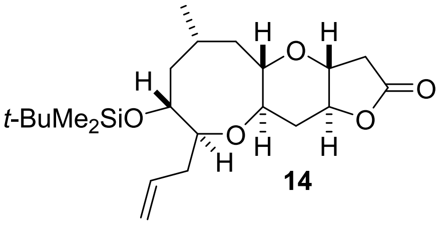

14

The aldehyde **13** (1.02 g, 2.47 mmol) was dissolved in a mixture of *t*-butanol (10 mL) and 2-methyl-2-butene (4.0 mL, 38 mmol) and the mixture was stirred at 0 °C. A solution of sodium chlorite (904 mg, 10.0 mmol) was added dropwise followed by a solution of sodium dihydrogen phosphate dihydrate (1.60 g, 10.3 mmol) in water (7.5 mL). The reaction mixture was stirred at 0 °C for 5 min and then warmed to room temperature. The resulting green biphasic solution was stirred for 8 h (the reaction progressively turned into a cloudy colourless solution) after which the volatile organics were removed in vacuo to leave a watery residue. The residue was diluted with ethyl acetate (10 mL) and brine (10 mL) and the aqueous phase was then extracted with ethyl acetate (3 × 10 mL). The organic extracts were dried (magnesium sulfate) and the solvent was removed in vacuo to

give an oil that was filtered through short pad of silica gel to deliver the carboxylic acid, which was used in next step without further purification.

To a solution of crude carboxylic acid (1.0 g, 2.3 mmol) in anhydrous toluene (100 mL) was added freshly distilled triethylamine (1.14 mL, 8.19 mmol) at room temperature. Freshly distilled 2,4,6-trichlorobenzoyl chloride (0.73 mL, 4.67 mmol) was added dropwise, and the resulting clear, colourless solution was stirred at room temperature for 1 h. Dimethylaminopyridine (1.0 g, 8.2 mmol) was added resulting in the formation of a white suspension. The mixture was stirred for a further 16 h at room temperature and the reaction mixture was loaded directly to a silica gel column. Flash column chromatography on silica gel (pet. ether-diethyl ether, 4:1) delivered the lactone **14** as a colourless oil (765 mg, 75% over two steps). R_f = 0.50 (pet. ether-diethyl ether, 4:1). $[\alpha]_D^{25}$ −35 (*c* = 0.2, $CHCl_3$). ν_{max} (film) 2954, 2928, 2857, 1798, 910, 832, 774, 731 cm^{-1}. ^1H NMR (400 MHz, $CDCl_3$) δ 5.88–5.77 (1H, m), 5.14–5.04 (2H, m), 3.80–3.38 (5H, m), 3.32 (1H, ddd, *J* = 9.3, 8.1, 2.8 Hz), 2.78–2.67 (2H, m), 2.62–2.48 (1H, m), 2.56 (1H, dd, *J* = 15.9, 11.9 Hz), 2.06–1.91 (2H, m), 1.90–1.82 (1H, m), 1.78 (1H, app. dt, *J* = 15.0, 2.9 Hz), 1.74–1.60 (3H, m), 1.03 (3H, d, *J* = 7.2 Hz), 0.88 (9H, s), 0.07 (3H, s), 0.05 (3H, s). ^{13}C NMR (101 MHz, $CDCl_3$) δ 173.2, 135.8, 117.6, 88.8, 82.4, 81.0, 78.6, 78.2, 75.6, 46.2, 44.4, 38.9, 36.8, 35.8, 28.0, 27.7, 26.0, 18.0, −4.0, −4.6. HRMS (ESI+) *m*/*z*: $[M + Na]^+$ Calcd for $C_{22}H_{38}NaO_5Si$ 433.2381; Found 433.2378.

Lactone S3.

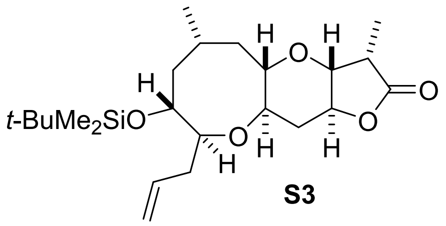

S3

To a stirred solution of lactone **14** (750 mg, 1.83 mmol) in THF (10 mL) at −78 °C was added lithium bis(trimethylsilyl)amide (2.20 mL of a 1.0 M solution in THF, 2.2 mmol) dropwise over 5 min. The mixture was stirred at −78 °C for 1 h, then methyl iodide (0.45 mL, 7.2 mmol) was added dropwise. The mixture was stirred at −78 °C for a further 1 h and then the reaction was quenched by addition of a saturated aqueous solution of ammonium chloride (10 mL). The mixture was warmed to room temperature and extracted with diethyl ether (3 × 20 mL). The combined organic extracts were washed with brine and dried (magnesium sulfate) and concentrated. The residue was purified by flash chromatography on silica gel (pet. ether-diethyl ether, 4:1) to afford the methylated lactone **S3** (710 mg, 92%) as colourless oil. R_f = 0.50 (pet. ether-ethyl acetate, 9:1). $[\alpha]_D^{25}$ −62 (*c* = 0.3, $CHCl_3$). ν_{max} (film) 2953, 2929, 2857, 1791, 868, 834, 774, 732 cm^{-1}. ^1H NMR (500 MHz, $CDCl_3$) δ 5.86–5.73 (1H, m), 5.09–4.98 (2H, m), 3.81 (1H, ddd, *J* = 12.2, 9.4, 3.8 Hz), 3.54 (1H, dd, *J* = 9.4, 7.7 Hz), 3.50–3.34 (2H, m), 3.38 (1H, ddd, *J* = 10.4, 9.2, 3.7 Hz), 3.27 (1H, ddd, *J* = 9.5, 8.0, 2.9 Hz), 2.77 (1H, dq, *J* = 7.7, 7.6 Hz), 2.67 (1H, ddd, *J* = 11.1, 4.9, 3.8 Hz), 2.50–2.43 (1H, m), 2.00–1.86 (2H, m), 1.84–1.78 (1H, m), 1.74 (1H, app. dt, *J* = 14.8, 2.9 Hz),

1.71–1.48 (3H, m), 1.15 (3H, d, *J* = 7.6 Hz), 0.99 (3H, d, *J* = 7.2 Hz), 0.83 (9H, s), 0.02 (3H, s), 0.00 (3H, s). ^{13}C NMR (126 MHz, $CDCl_3$) δ 177.2, 135.8, 117.6, 88.7, 82.4, 81.1, 80.0, 75.6, 75.0, 46.0, 44.4, 39.0, 38.9, 37.1, 28.1, 27.7, 26.0, 18.0, 8.4, −4.0, −4.6. HRMS (ESI+) *m/z*: $[M+Na]^+$ Calcd for $C_{23}H_{40}NaO_5Si$ 447.2537; Found 447.2530.

Lactone 15.

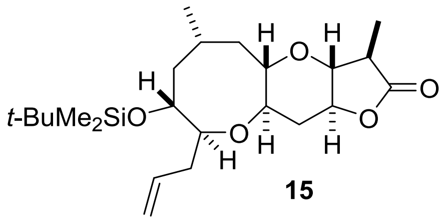

15

To a stirred solution of methyl lactone **S3** (710 mg, 1.67 mmol) in THF (8.5 mL) at −78 °C was added lithium diisopropylamide (2.5 mL of a 1.0 M solution in THF, 2.5 mmol) dropwise over 10 min. The mixture was stirred at −78 °C for 1 h and then the reaction was quenched by the addition of a saturated aqueous solution of ammonium chloride (10 mL). The mixture was warmed to room temperature and extracted with diethyl ether (3 × 20 mL). The combined organic extracts were washed with brine, dried (magnesium sulfate) and concentrated. The residue was purified by flash column chromatography on silica gel (pet. ether-diethyl ether, 4:1) to afford the lactone **15** (560 mg, 79%) as colourless oil. R_f = 0.50 (pet. ether-ethyl acetate, 9:1). $[\alpha]_D^{25}$ −44 (*c* = 0.15, $CHCl_3$). ν_{max} (film) 2953, 2929, 2857, 1793, 878, 834, 774 cm^{-1}. ^{1}H NMR (400 MHz, $CDCl_3$) δ 5.82 (1H, dddd, *J* = 17.3, 9.6, 7.6, 6.7 Hz), 5.13–5.05 (2H, m), 3.60 (1H, ddd, *J* = 12.3, 9.0, 3.8 Hz), 3.57–3.45 (2H, m), 3.40 (1H, ddd, *J* = 10.3, 9.5, 3.6 Hz), 3.32 (1H, ddd, *J* = 9.4, 8.2, 2.8 Hz), 3.17 (1H, dd, *J* = 11.6, 9.0 Hz), 2.69 (1H, ddd, *J* = 11.2, 4.8, 4.1 Hz), 2.63–2.53 (1H, m), 2.55–2.47 (1H, m), 2.05–1.86 (3H, m), 1.78 (1H, app. dt, *J* = 14.8, 2.8 Hz), 1.73–1.55 (3H, m), 1.28 (3H, d, *J* = 6.9 Hz), 1.04 (3H, d, *J* = 7.1 Hz), 0.88 (9H, s), 0.06 (3H, s), 0.04 (3H, s). ^{13}C NMR (101 MHz, $CDCl_3$) δ 176.3, 135.8, 117.6, 88.7, 84.4, 82.3, 81.2, 76.4, 75.6, 46.1, 44.4, 41.7, 38.9, 36.7, 28.0, 27.6, 26.0, 18.0, 12.2, −4.0, −4.6. HRMS (ESI+) *m/z*: $[M-H]^+$ Calcd for $C_{23}H_{39}O_5Si$ 423.2572; Found 423.2566.

Aldehyde 16.

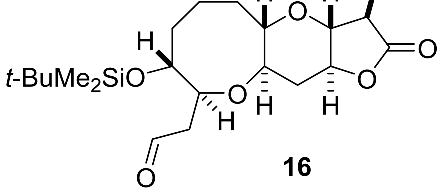

16

To a stirred solution of alkene **15** (200 mg, 0.471 mmol) in THF (10 mL), *t*-butanol (10 mL) and water (2.0 mL) was added sequentially osmium tetroxide (0.25 mL of a 4% wt solution in water, 0.04 mmol) and *N*-methylmorpholine-*N*-oxide monohydrate (127 mg, 0.940 mmol). The mixture

was stirred at room temperature for 16 h and the reaction was quenched by addition of a saturated aqueous solution of sodium thiosulfate (20 mL). The mixture was stirred for 2 h and the phases were separated. The aqueous phase extracted with ethyl acetate (3 × 20 mL) and the combined organic extracts were dried (magnesium sulfate) and concentrated. The crude diol was used in next step without purification.

To a stirred solution of above diol in dichloromethane (20 mL) was added sodium periodate on adsorbed silica (10% by weight, 7.50 g). The suspension was stirred for 1 h before the mixture was passed through a short plug of silica. The solution was concentrated and the residue was purified by flash column chromatography on silica gel (pet. ether-diethyl ether, 2:1) to give the aldehyde **16** (160 mg, 80% over two steps) as a colourless oil. R_f = 0.25 (pet. ether-diethyl ether, 7:3). $[\alpha]_D^{25}$ −24 (*c* = 0.25, $CHCl_3$). ν_{max} (film) 2930, 2857, 1788, 1721, 834, 774, 732 cm^{-1}. ^1H NMR (400 MHz, $CDCl_3$) δ 9.81 (1H, dd, *J* = 2.1, 0.9 Hz), 3.92 (1H, td, *J* = 8.9, 2.9 Hz), 3.70–3.60 (2H, m), 3.48 (1H, ddd, *J* = 9.3, 8.5, 3.1 Hz), 3.34 (1H, ddd, *J* = 10.6, 9.3, 3.3 Hz), 3.14 (1H, dd, *J* = 11.6, 9.0 Hz), 2.83 (1H, ddd, *J* = 17.2, 2.8, 0.9 Hz), 2.64–2.52 (3H, m), 1.96–1.84 (2H, m), 1.81 (1H, app. dt, *J* = 14.8, 2.8 Hz), 1.77–1.59 (2H, m), 1.58–1.47 (1H, m), 1.28 (3H, d, *J* = 7.0 Hz), 1.05 (3H, d, *J* = 7.1 Hz), 0.86 (9H, s), 0.07 (3H, s), 0.04 (3H, s). ^{13}C NMR (101 MHz, $CDCl_3$) δ 201.0, 176.2, 84.4, 83.1, 82.5, 82.0, 76.3, 75.0, 49.1, 46.5, 44.5, 41.6, 36.4, 28.2, 27.7, 25.9, 18.0, 12.2, −4.0, −4.6. HRMS (ESI+) *m/z*: $[M + H]^+$ Calcd for $C_{22}H_{39}O_6Si$ 427.2510; Found 427.2509.

Lactone 17.

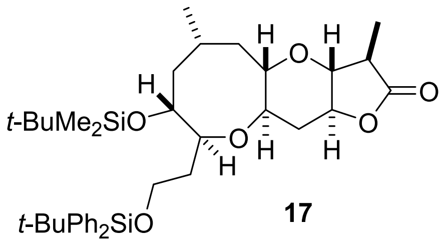

17

To a solution of aldehyde **15** (175 mg, 0.410 mmol) in a mixture of dichloromethane (4.0 mL) and methanol (4.0 mL) at −20 °C, was added sodium borohydride (16 mg, 0.42 mmol). The mixture was warmed to 0 °C and stirred for 30 min and then the reaction was quenched by the addition of a saturated aqueous solution of ammonium chloride (5 mL). The cooling bath was removed and the mixture was stirred vigorously at room temperature for 15 min The mixture was diluted with diethyl ether (10 mL) and washed with brine, dried (magnesium sulfate) and concentrated. The crude reaction mixture was filtered through short pad of silica gel to afford the alcohol, which was carried on next step.

To the solution of the alcohol (0.41 mmol) in DMF (3.5 mL) at room temperature were added imidazole (112 mg, 1.65 mmol) and *t*-butyl(chloro)diphenylsilane (0.21 mL, 0.81 mmol). The mixture was stirred at room temperature for 16 h and the reaction was quenching by the addition of methanol (0.2 mL). The reaction mixture was stirred for a further 2 h and then extracted with diethyl

ether (3 × 5 mL). The combined organic extracts were washed with brine, dried (magnesium sulfate) and concentrated. The residue was purified by flash column chromatography on silica gel (pet. ether-diethyl ether, 4:1) to afford product **17** (190 mg, 69% over two steps) as a colourless oil. R_f = 0.35 (pet. ether-diethyl ether, 4:1). $[\alpha]_D^{25}$ −12 (*c* = 0.25, $CHCl_3$). ν_{max} (film) 2956, 2929, 2856, 1797, 937, 881, 834, 772, 701 cm^{-1}. ^1H NMR (400 MHz, $CDCl_3$) δ 7.68–7.66 (1H, m), 7.64–7.59 (3H, m), 7.39–7.32 (6H, m, Ph), 3.77–3.66 (2H, m), 3.66–3.59 (1H, m), 3.58 (1H, app. td, *J* = 9.9, 4.8 Hz), 3.52–3.41 (3H, m), 3.33 (1H, app. td, *J* = 9.9, 3.6 Hz), 3.10 (1H, dd, *J* = 11.6, 9.0 Hz), 2.61–2.50 (2H, m), 2.08–1.99 (1H, m), 1.98–1.89 (1H, m), 1.89–1.82 (1H, m), 1.81–1.60 (3H, m), 1.50 (1H, app. dt, *J* = 11.4, 11.1 Hz), 1.25 (3H, d, *J* = 6.8 Hz), 1.02 (3H, d, *J* = 7.0 Hz), 1.02 (9H, s), 0.86 (9H, s), 0.03 (3H, s), 0.00 (3H, s). ^{13}C NMR (126 MHz, $CDCl_3$) δ 176.5, 134.9, 133.9, 133.8, 129.9, 129.8, 127.9, 127.8, 84.4, 84.1, 82.5, 80.7, 76.5, 76.1, 66.0, 60.1, 44.1, 41.7, 37.0, 36.1, 28.1, 27.0, 26.0, 19.4, 18.0, 15.4, 12.2, −4.0, −4.7. HRMS (ESI+) *m*/*z*: $[M + H]^+$ Calcd for $C_{38}H_{59}O_6Si_2$ 667.3845; Found 667.3843.

Protected Bicyclic Ether 18.

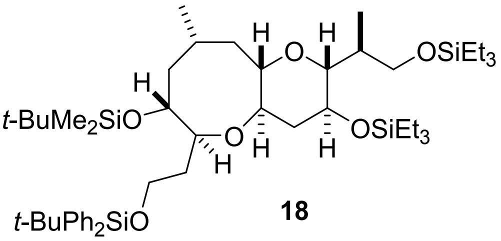

18

To a solution of lactone **17** (240 mg, 0.360 mmol) in THF (1.5 mL) at 0 °C, were added lithium borohydride (0.45 mL of a 2.0 M solution in THF, 0.90 mmol) and methanol (15 μL). The mixture was stirred at 0 °C for 1 h and the warmed to room temperature and stirred for 1 h. The reaction was quenched by the addition of a saturated aqueous solution of ammonium chloride (2.0 mL) and the resulting mixture was stirred vigorously at room temperature for 15 min. The mixture was diluted with ethyl acetate (5 mL) and then washed with brine, dried (magnesium sulfate) and concentrated. Residual material was filtered through short pad of silica gel to afford the diol (160 mg) as a product which was taken forward to the next step without purification.

To the solution of diol (160 mg, 0.238 mmol) in dichloromethane (3.0 mL) was added imidazole (130 mg, 1.91 mmol) followed by chlorotriethylsilane (0.16 mL, 0.95 mmol). The mixture was stirred at room temperature for 2 h and then diluted with water (2 mL). The phases were separated and the aqueous layer was extracted with diethyl ether (2 × 3 mL). The combined organic extracts were dried (magnesium sulfate) and the solvent was removed under reduced pressure. The product was purified by flash column chromatography on silica gel (pet. ether-diethyl ether, 10:1) to give the bicyclic ether **18** (200 mg, 62% over two steps) as a colourless oil. R_f = 0.78 (pet. ether-diethyl ether, 17:3). $[\alpha]_D^{25}$ −46 (*c* = 0.15, $CHCl_3$). ν_{max} (film) 2952, 2929, 2923, 2875, 2860, 834, 774, 735, 701 cm^{-1}. ^1H NMR (500 MHz, $CDCl_3$) δ 7.70–7.65 (4H, m, Ph), 7.44–7.34 (6H, m), 3.87 (1H, td, *J* = 10.0, 4.8 Hz), 3.81 (1H, ddd, *J* = 10.0, 6.4, 3.2 Hz), 3.74–3.68 (1H, m), 3.60 (1H, dd, *J* = 9.4, 9.1

Hz), 3.55–3.36 (4H, m), 3.17 (1H, dd, *J* = 9.2, 1.4 Hz), 3.05 (1H, ddd, *J* = 10.0, 9.4, 2.9 Hz), 2.29 (1H, app. dt, *J* = 11.8, 4.6 Hz), 2.13–2.01 (2H, m), 1.98–1.90 (1H, m), 1.86 (1H, app. dt, *J* = 14.7, 3.1 Hz), 1.80 (1H, app. dt, *J* = 14.2, 3.1 Hz), 1.74–1.58 (2H, m), 1.50–1.40 (1H, m), 1.38–1.30 (1H, m), 1.07 (9H, s), 1.06 (3H, d, *J* = 7.4 Hz), 0.96 (9H, t, *J* = 7.9 Hz), 0.90 (9H, s), 0.90 (9H, t, *J* = 7.9 Hz), 0.76 (3H, d, *J* = 7.0 Hz), 0.60 (6H, q, *J* = 7.9 Hz), 0.54 (6H, q, *J* = 7.9 Hz), 0.07 (3H, s), 0.02 (3H, s). ^{13}C NMR (101 MHz, $CDCl_3$) δ 135.8, 135.7, 135.6, 134.4, 134.2, 129.7, 129.7, 127.8, 82.6, 80.8, 80.8, 80.0, 76.3, 67.0, 65.5, 60.8, 43.6, 42.1, 37.5, 35.1, 28.4, 27.0, 26.9, 26.0, 19.5, 18.0, 15.4, 9.5, 7.0, 7.0, 5.3, 4.6, −3.9, −4.7. HRMS (ESI+) *m*/*z*: $[M + Na]^+$ Calcd for $C_{50}H_{90}NaO_6Si_4$ 921.5707; Found 921.5700.

Aldehyde 19.

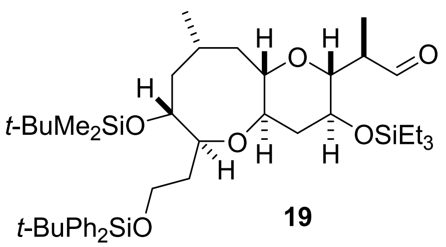

19

To a stirred solution of oxalyl chloride (0.22 mL of a 2.0 M solution in dichloromethane, 0.44 mmol) in dichloromethane (1.7 mL) at −78 °C was added DMSO (65 µL, 0.92 mmol) dropwise. The mixture was stirred for 30 min at −78 °C and then a solution of the bicyclic ether **18** (80 mg, 0.089 mmol) in dichloromethane (0.3 mL) was added dropwise over 10 min. The temperature was raised to −55 °C and the mixture stirred at for 1 h. The mixture was cooled to −78 °C and triethylamine (0.22 mL, 1.60 mmol) was added dropwise. The mixture was warmed to 0 °C over a 30 min period and stirred at 0 °C for 30 min. The reaction was quenched by the addition of a saturated aqueous solution of ammonium chloride (2.0 mL) and the phases were separated. The aqueous phase was extracted with diethyl ether (2 × 5 mL) and the combined organic extracts were dried (magnesium sulfate), and concentrated. The residue was purified by flash column chromatography on silica gel (pet. ether-diethyl ether, 10:1) to afford the aldehyde **19** (56 mg, 80%) as a colourless oil. R_f = 0.50 (pet. ether-diethyl ether, 17:3). $[\alpha]_D^{25}$ −8.4 (*c* = 0.125, $CHCl_3$). ν_{max} (film) 2952, 2929, 2875, 2857, 1731, 834, 823, 775 736 cm^{-1}. ^{1}H NMR (400 MHz, $CDCl_3$) δ 9.66 (1H, d, *J* = 1.0 Hz), 7.69–7.64 (4H, m), 7.45–7.33 (6H, m), 3.90–3.76 (2H, m), 3.71 (1H, ddd, *J* = 10.2, 7.9, 2.4 Hz), 3.54 (1H, td, *J* = 9.0, 3.0 Hz), 3.52–3.40 (3H, m), 3.13 (1H, app. td, *J* = 9.5, 3.6 Hz), 2.68–2.60 (1H, m), 2.34 (1H, app. dt, *J* = 11.8, 4.5 Hz), 2.10–1.99 (1H, m), 1.98–1.89 (1H, m), 1.84 (1H, app. dt, *J* = 14.8, 3.1 Hz), 1.78–1.54 (3H, m), 1.53–1.41 (1H, m), 1.39–1.29 (1H, m), 1.07 (9H, s), 1.05 (3H, d, *J* = 7.1 Hz), 1.03 (3H, d, *J* = 7.0 Hz), 0.90 (9H, t, *J* = 7.9 Hz), 0.89 (9H, m), 0.57–0.48 (6H, m), 0.06 (3H, s), 0.01 (3H, s). ^{13}C NMR (101 MHz, $CDCl_3$) δ 204.3, 135.7, 135.6, 134.3, 134.1, 129.7, 129.7, 127.8, 127.8, 82.7, 81.2, 80.1, 80.1, 76.2, 67.3, 60.7, 47.2, 43.2, 41.7, 37.4, 28.3, 28.3, 27.0, 26.8, 26.0, 19.5, 18.0, 7.1, 6.9, 5.2, −3.9, −4.7. HRMS (ESI+) *m*/*z*: $[M + H]^+$ Calcd for $C_{44}H_{75}O_6Si_3$ 783.4866; Found 783.4867.

Alcohol 21.

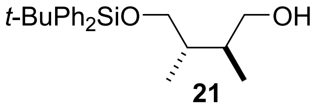

21

To a solution of diol **20** (120 mg, 1.00 mmol) in THF (5.0 mL) at 0 °C was added sodium hydride (40.0 mg of 60% dispersion in mineral oil, 1.2 mmol).[2,3] The resulting mixture was stirred at 0 °C for 30 min and warmed slowly to room temperature and stirred for 2 h. To this solution, *t*-butyl(chloro)diphenylsilane (274 mg, 1.00 mmol) was added and the mixture was stirred at room temperature for 14 h. The reaction was quenched by the addition of a saturated aqueous solution of ammonium chloride (5 mL). The solution was diluted with diethyl ether (10 mL), washed with brine, dried (magnesium sulfate) and concentrated. The residue was purified by flash column chromatography on silica gel (pet. ether-diethyl ether, 2:1) to deliver the alcohol **21** (215 mg, 59%) as a colourless oil. R_f = 0.40 (pet. ether-diethyl ether, 7:3). $[\alpha]_D^{25}$ −4.5 (*c* = 0.75, $CHCl_3$). ν_{max} (film) 3337, 2957, 2928, 2857, 822, 738, 699 cm^{-1}. ^1H NMR (500 MHz, $CDCl_3$) δ 7.71–7.65 (4H, m), 7.47–7.37 (6H, m), 3.63–3.51 (4H, m), 1.79 (2H, br s), 1.08 (9H, s), 0.87 (3H, d, *J* = 6.5 Hz), 0.83 (3H, d, *J* = 6.6 Hz). ^{13}C NMR (126 MHz, $CDCl_3$) δ 135.8, 135.7, 133.7, 133.6, 129.8, 129.8, 127.8, 127.8, 67.5, 66.6, 37.8, 37.4, 27.0, 19.4, 13.4. HRMS (ESI+) *m*/*z*: $[M + Na]^+$ Calcd for $C_{22}H_{32}NaO_2Si$ 379.2064; Found 379.2059.

Alkyne 23.

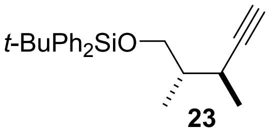

23

To the solution of alcohol **21** (155 mg, 0.435 mmol) in dichloromethane (20 mL) at room temperature was added sodium bicarbonate (184 mg, 2.20 mmol) followed by Dess-Martin periodinane (373 mg, 0.879 mmol). The mixture was stirred for 5 h at room temperature and diluted with a mixture of saturated aqueous sodium thiosulfate solution and saturated aqueous sodium bicarbonate solution (1:1, 12 mL). The mixture was stirred vigorously for 15 min and then extracted with diethyl ether (3 × 20 mL). The phases were separated and the organic phase was washed with brine, dried (magnesium sulfate) and concentrated under reduced pressure. The resulting crude aldehyde **22** was used in next step without purification.

A solution of dimethyl (1-diazo-2-oxopropyl) phosphonate, the Ohira-Bestmann reagent, (253 mg, 1.32 mmol) in dry THF (10 mL) at −78 °C was treated dropwise with sodium methoxide (0.29 mL of 25% solution in methanol, 1.32 mmol) and the mixture was stirred for 20 min at this temperature. A solution of crude aldehyde **20** in dry THF (1.0 mL) was added dropwise at −78 °C and the mixture was allowed to warm to room temperature over a period of 30 min. The reaction was quenched by the addition of a saturated aqueous solution of ammonium chloride (10 mL). The mixture was extracted with diethyl ether (3 × 10 mL) and the combined organic extracts were washed with brine

(5 mL) and dried (magnesium sulfate). The solvent was removed under reduced pressure and the residue was purified by flash column chromatography on silica gel (pet. ether-diethyl ether, 10:1). to give the alkyne **23** (95.0 mg, 62% over two steps) as a colourless oil. R_f = 0.56 (pet. ether-diethyl ether, 17:3). $[\alpha]_D^{25}$ +3.1 (*c* = 0.5, $CHCl_3$). ν_{max} (film) 3309, 3071, 2958, 2930, 2857, 823, 739, 700 cm^{-1}. ^1H NMR (500 MHz, $CDCl_3$) δ 7.71–7.65 (4H, m), 7.45–7.35 (6H, m), 3.65 (2H, d, *J* = 6.1 Hz), 2.68 (1H, qdd, *J* = 7.1, 6.0, 2.5 Hz), 2.00 (1H, d, *J* = 2.5 Hz), 1.83 (1H, qtd, *J* = 6.9, 6.1, 6.0 Hz), 1.11 (3H, d, *J* = 7.1 Hz), 1.06 (9H, s), 0.98 (3H, d, *J* = 6.9 Hz). ^{13}C NMR (126 MHz, $CDCl_3$) δ 135.8, 135.8, 134.0, 134.0, 129.7, 129.7, 127.7, 127.7, 88.5, 68.8, 66.5, 40.3, 27.5, 27.0, 19.4, 16.9, 13.9. HRMS (ESI+) *m*/*z*: $[M + Na]^+$ Calcd for $C_{23}H_{30}NaOSi$ 373.1958; Found 373.1948.

Alkyne 24.

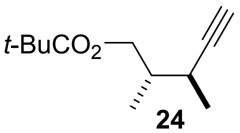

24

To a solution of alkyne **23** (200 mg, 0.570 mmol) in THF (6 mL) was added tetra-*n*-butylammonium fluoride (1.14 mL of a 1.0 M solution in hexane, 1.14 mmol) at room temperature. The mixture was stirred at room temperature for 2 h and then the reaction was quenched by the addition of a saturated aqueous solution of ammonium chloride (5 mL). The mixture was vigorously stirred at room temperature for 15 min and then diluted with diethyl ether (5 mL). The phases were separated and the organic phase was washed with brine, dried (magnesium sulfate) and concentrated under reduced pressure to afford the crude alcohol, which was used in next step without purification.

To the solution of the alcohol in a mixture of dichloromethane and pyridine (1:1, 1.5 mL) at room temperature was added trimethylacetyl chloride (0.14 mL, 1.14 mmol). The mixture was stirred for 5 h at room temperature and then diluted with water (2 mL). The mixture was extracted with diethyl ether (3 × 2 mL) and the combined organic extracts were washed with brine and dried (magnesium sulfate). The solvent removed carefully *in vacuo* and the residue was purified by flash column chromatography on silica gel (pet. ether-diethyl ether, 10:1) to give the alkyne **24** (95 mg, 85% over two steps) as colourless oil. R_f = 0.87 (pet. ether-diethyl ether, 4:1). $[\alpha]_D^{25}$ +47 (*c* = 0.5, $CHCl_3$). ν_{max} (film) 3314, 2973, 2180, 2014, 1729 cm^{-1}. ^1H NMR (400 MHz, $CDCl_3$) δ 4.14 (1H, dd, *J* = 11.0, 5.7 Hz), 4.01 (1H, dd, *J* = 11.0, 6.7 Hz), 2.51 (1H, qdd, *J* = 6.9, 6.6, 2.5 Hz), 2.06 (1H, d, *J* = 2.5 Hz), 1.91 (1H, dqdd, *J* = 6.7, 6.6, 6.6, 5.7 Hz), 1.20 (9H, s), 1.19 (3H, d, *J* = 6.6 Hz), 1.01 (3H, d, *J* = 6.9 Hz). ^{13}C NMR (101 MHz, $CDCl_3$) δ 178.6, 87.0, 69.7, 67.0, 39.0, 37.3, 28.6, 27.3, 17.7, 14.7. HRMS (ESI+) *m*/*z*: $[M - H]^+$ Calcd for $C_{12}H_{19}O_2$ 195.1391; Found 195.1391.

Iodoalkyne 25.

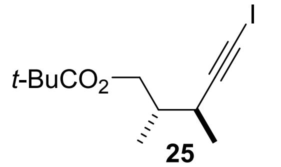

25

To a flame dried flask was added iodine (1.74 g, 6.86 mmol) followed by anhydrous toluene (5.0 mL) The mixture was stirred for 15 min until iodine had dissolved. Morpholine (1.2 mL, 13.8 mmol) was then added dropwise and the resulting mixture was sonicated for 10 min. The mixture was then heated to 45 °C (oil bath) and stirred at this temperature for 30 min. A solution of alkyne **24** (90 mg, 0.46 mmol) in toluene (5.0 mL) was added and the resulting mixture was stirred at 45 °C overnight. The reaction was quenched by the addition of a saturated aqueous solution of sodium thiosulfate (10 mL). The resulting biphasic mixture was stirred vigorously for 30 min and then extracted by diethyl ether (3 × 20 mL). The combined organic extracts were dried (magnesium sulfate) and the solvent was removed carefully *in vacuo*. The residue was purified by flash column chromatography on silica gel (pet. ether-diethyl ether, 20:1) to afford the iodoalkyne **25** (128 mg, 87%) as colourless oil. R_f = 0.87 (pet. ether-diethyl ether, 4:1). $[\alpha]_D^{25}$ +59 (c = 0.5, $CHCl_3$). ν_{max} (film) 2972, 2933, 2876, 2190, 1729 cm^{-1}. ^{1}H NMR (400 MHz, $CDCl_3$) δ 4.11 (1H, dd, J = 11.0, 5.8 Hz), 3.97 (1H, dd, J = 11.0, 6.6 Hz), 2.68 (1H, qd, J = 7.1, 5.9 Hz), 1.91 (1H, qddd, J = 6.9, 6.6, 5.9, 5.8 Hz), 1.20 (9H, s), 1.18 (3H, d, J = 7.1 Hz), 1.00 (3H, d, J = 6.9 Hz). ^{13}C NMR (101 MHz, $CDCl_3$) δ 178.6, 97.3, 66.9, 39.0, 37.5, 31.0, 27.4, 17.6, 14.8.

Propargylic Alcohol S4.

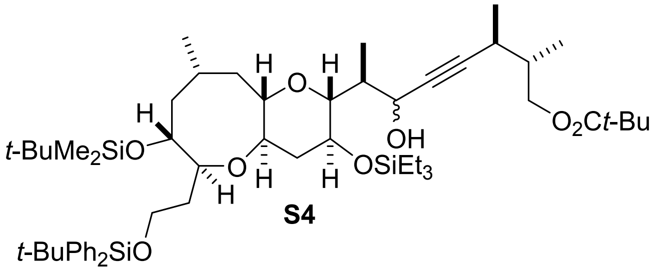

S4

The aldehyde **19** (60.0 mg, 0.0766 mmol) and iodoalkyne **25** (50.0 mg, 0.155 mmol) was azeotroped with toluene (3 × 2 mL) and placed under a nitrogen atmosphere. Anhydrous THF (5 mL) was added and the placed in a glovebox. Chromium(II) chloride (113.0 mg, 0.919 mmol) was added and then the solution was stirred under nitrogen at room temperature for 15 h. The reaction was quenched by the addition of water (0.5 mL) and the aqueous phase was extracted using diethyl ether (4 × 10 mL). The combined organic extracts were dried (magnesium sulfate) and concentrated. The residue was purified by flash column chromatography on silica gel (pet. ether-diethyl ether, 4:1) to yield the alcohol **S4** (mixture of diastereomers, *dr* 1:0.8) as a colourless oil (60 mg, 80%). R_f = 0.20 (pet. ether-diethyl ether, 17:3). ν_{max} (film) 3491, 2956, 2928, 2857, 1732, 835, 774, 736, 702 cm^{-1}. ^{1}H NMR (500 MHz, $CDCl_3$) δ 7.68–7.63 (4H, m), 7.44–7.33 (6H, m), 4.62

(0.5H, br s), 4.37 (0.5H, ddd, J = 8.8, 4.3, 1.7 Hz), 4.14 (1H, ddd, J = 10.8, 8.6, 6.0 Hz), 3.97 (1H, ddd, J = 11.1, 6.8, 5.6 Hz), 3.89–3.67 (3H, m), 3.55–3.44 (3H, m), 3.35–3.26 (1H, m), 3.23–3.13 (1H, m), 2.63–2.54 (1H, m), 2.33–2.25 (1H, m), 2.15–1.88 (3H, m), 1.87–1.67 (3H, m), 1.64–1.54 (2H, m), 1.49–1.38 (1H, m), 1.20 (9H, s), 1.17 (3H, m), 1.06 (9H, s), 1.05–0.98 (9H, m), 0.91 (9H, s), 0.91–0.86 (9H, m), 0.56–0.48 (6H, m), 0.06 (3H, s), 0.01 (3H, s). ^{13}C NMR (101 MHz, $CDCl_3$) δ 178.5, 178.5, 135.6, 135.6, 134.3, 134.3, 134.1, 134.1, 129.7, 129.7, 127.8, 127.8, 88.1, 87.3, 85.5, 82.8, 82.6, 82.0, 81.2, 81.0, 79.6, 79.4, 77.5, 76.2, 76.2, 68.0, 67.5, 67.0, 66.7, 66.0, 60.7, 43.5, 43.3, 41.8, 41.7, 39.0, 38.8, 37.9, 37.4, 37.4, 37.3, 30.5, 29.8, 28.7, 28.2, 28.1, 27.4, 27.0, 26.8, 26.0, 19.4, 18.0, 17.5, 17.5, 15.4, 14.6, 14.5, 10.4, 7.0, 6.9, 6.6, 5.3, 5.2, −4.0, −4.0, −4.7. HRMS (ESI+) m/z: [M + Na]$^+$ Calcd for $C_{56}H_{94}NaO_8Si_3$ 1001.6149; Found 1001.6164.

Propargylic Ketone 26.

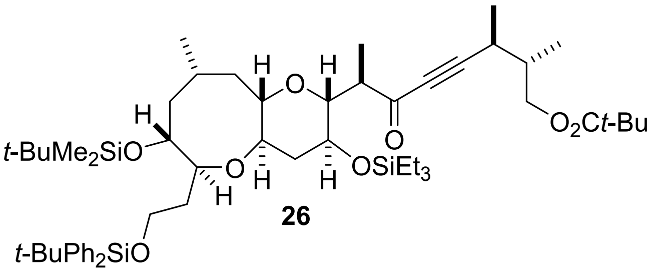

26

To the solution of alcohol **S4** (60.0 mg, 0.0612 mmol) in dichloromethane (6.0 mL) at room temperature was added 2,6-lutidine (36.0 µL, 0.311 mmol) followed by Dess-Martin periodinane (52.0 mg, 0.123 mmol). The mixture was stirred for 5 h at room temperature and then diluted with a mixture of saturated aqueous sodium thiosulfate and saturated aqueous sodium bicarbonate (1:1, 12 mL). The mixture was stirred vigorously for 15 min and then extracted with diethyl ether (3 × 20 mL). The combined organic extracts were washed with brine, dried (magnesium sulfate) and concentrated. The residue was purified by flash column chromatography on silica gel (pet. ether-diethyl ether, 10:1) to deliver the propargylic ketone **26** (50.0 mg, 84%) as a colourless liquid. R_f = 0.50 (pet. ether-diethyl ether, 17:3). $[\alpha]_D^{25}$ −30 (c = 0.2, $CHCl_3$). ν_{max} (film) 2957, 2926, 2855, 1732, 1678, 836, 774, 737, 702 cm^{-1}. ^{1}H NMR (500 MHz, $CDCl_3$) δ 7.68–7.64 (4H, m), 7.44–7.33 (6H, m), 4.12 (1H, dd, J = 11.1, 6.3 Hz), 3.98 (1H, dd, J = 11.1, 6.5 Hz), 3.85 (1H, app. td, J = 9.9, 4.7 Hz), 3.80 (1H, ddd, J = 10.2, 6.5, 3.3 Hz), 3.72 (1H, dd, J = 9.1, 3.0 Hz), 3.71–3.64 (1H, m), 3.53–3.39 (3H, m), 3.10 (1H, app. td, J = 9.8, 3.1 Hz), 2.81 (1H, qd, J = 6.9, 3.0 Hz), 2.75 (1H, qd, J = 7.1, 5.4 Hz), 2.32 (1H, app. dt, J = 11.9, 4.6 Hz), 2.08–1.99 (2H, m), 1.97–1.87 (1H, m), 1.84 (1H, app. dt, J = 14.8, 3.1 Hz), 1.77 (1H, app. dt, J = 14.2, 3.1 Hz), 1.70–1.63 (1H, m), 1.63–1.55 (2H, m), 1.47 (1H, app. q, J = 11.3 Hz), 1.23 (3H, d, J = 7.1 Hz), 1.21 (9H, s), 1.09 (3H, d, J = 6.9 Hz), 1.06 (9H, s), 1.04 (3H, d, J = 6.7 Hz), 1.02 (3H, d, J = 6.8 Hz), 0.89 (9H, t, J = 7.9 Hz), 0.88 (9H, s), 0.53 (6H, q, J = 7.9 Hz), 0.05 (3H, s), 0.00 (3H, s). ^{13}C NMR (101 MHz, $CDCl_3$) δ 190.2, 178.4, 135.7, 135.6, 134.3, 134.1, 129.7, 129.7, 127.8, 96.2, 82.7, 81.4, 81.3, 81.3, 80.3, 76.2, 67.6, 66.5,

66.0, 48.8, 43.3, 41.8, 39.0, 37.5, 37.0, 28.8, 28.3, 27.3, 27.0, 26.0, 19.5, 18.0, 16.3, 14.4, 8.4, 7.0, 5.3, −3.9, −4.7. HRMS (ESI+) *m*/*z*: $[M + Na]^+$ Calcd for $C_{56}H_{92}NaO_8Si_3$ 999.5992; Found 999.5979.

Alcohol S5.

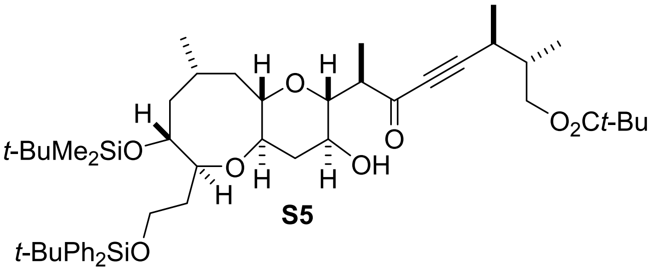

S5

To the solution of propargylic ketone **26** (50.0 mg, 0.0511 mmol) in a mixture of THF (1.50 mL) and water (0.75 mL), was added acetic acid (3.80 mL) and the mixture was stirred at room temperature for 24 h. The solution was diluted with diethyl ether (2.0 mL) and a saturated aqueous solution of sodium bicarbonate (4.0 mL). The aqueous phase was extracted with diethyl ether (4 × 20 mL) and the combined organic extracts were washed with brine, dried (magnesium sulfate) concentrated. The residue was purified by flash column chromatography on silica gel (pet. ether-diethyl ether, 2:1). to give the alcohol **S5** (40.0 mg, 91%) as an oil. R_f = 0.14 (pet. ether-diethyl ether, 4:1). $[\alpha]_D^{25}$ −9.0 (*c* = 0.2, $CHCl_3$). ν_{max} (film) 3489, 2977, 2933, 2859, 1732, 835, 775, 702 cm^{-1}. ^1H NMR (500 MHz, $CDCl_3$) δ 7.70 (2H, d, *J* = 6.5 Hz), 7.65 (2H, d, *J* = 6.5 Hz), 7.46–7.34 (6H, m), 4.22 (1H, dd, *J* = 11.0, 5.1 Hz), 4.06 (1H, dd, *J* = 11.0, 7.0 Hz), 3.82 (1H, ddd, *J* = 10.3, 10.1, 4.1 Hz), 3.78–3.70 (2H, m), 3.65 (1H, ddd, *J* = 10.6, 8.1, 2.3 Hz), 3.51–3.44 (1H, m), 3.36 (1H, ddd, *J* = 11.0, 9.3, 4.6 Hz), 3.34–3.27 (1H, m), 3.07 (1H, ddd, *J* = 10.0, 9.6, 3.5 Hz), 2.92 (1H, qd, *J* = 6.9, 3.3 Hz), 2.61 (1H, app. p, *J* = 7.0 Hz), 2.36 (1H, app. dt, *J* = 11.7, 4.5 Hz), 2.14–2.06 (2H, m), 1.99–1.92 (1H, m), 1.93–1.85 (1H, m), 1.82–1.75 (2H, m), 1.68–1.54 (3H, m), 1.37 (1H, app. q, *J* = 11.4 Hz), 1.26 (3H, d, *J* = 7.1 Hz), 1.20 (9H, s), 1.10 (3H, d, *J* = 6.9 Hz), 1.06 (9H, s), 1.05 (3H, d, *J* = 6.9 Hz), 1.01 (3H, d, *J* = 7.1 Hz), 0.90 (9H, s), 0.06 (3H, s), 0.03 (3H, s). ^{13}C NMR (101 MHz, $CDCl_3$) δ 190.0, 179.0, 135.7, 134.3, 134.0, 129.7, 129.7, 127.8, 127.8, 95.7, 83.6, 81.4, 81.2, 81.1, 81.0, 76.2, 67.1, 67.0, 60.3, 49.4, 45.4, 43.9, 41.4, 39.1, 37.5, 37.1, 30.5, 29.1, 28.0, 27.3, 27.0, 26.0, 19.4, 18.0, 17.3, 15.1, 8.4, −3.9, −4.7. HRMS (ESI+) *m*/*z*: $[M + Na]^+$ Calcd for $C_{50}H_{78}NaO_8Si_2$ 885.5127; Found 885.5125.

Cyclic Enol Ether 27.

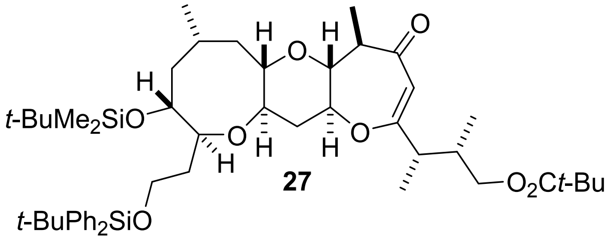

27

The alcohol **S5** (40.0 mg, 0.0463 mmol) was subjected to azeotropic removal of water with toluene (3 × 2 mL) and placed under an argon atmosphere. The alcohol was dissolved in anhydrous THF (1.0 mL) and hexafluoroisopropanol (15 μL, 0.14 mmol) was added to the solution. Tris[(triphenylphosphine)gold]oxonium tetrafluoroborate (10.2 mg, 0.00689 mmol) was added and the mixture was heated to 60 °C (oil bath) and stirred at that temperature for 14 h. After completion of the reaction (determined by TLC), the mixture was filtrated through a short pad of silica gel, eluting with diethyl ether, and the filtrate was concentrated. The residue was purified by flash column chromatography on silica gel (pet. ether-diethyl ether, 5:1) to give the corresponding cyclic enol ether **27** (29.0 mg, 73%) as a colourless oil. R_f = 0.50 (pet. ether-diethyl ether, 4:1). $[\alpha]_D^{25}$ −27 (*c* = 0.2, $CHCl_3$). ν_{max} (film) 2957, 2927, 2856, 1730, 1656, 1621, 936, 880, 835, 774, 736, 701 cm^{-1}. ^1H NMR (500 MHz, $CDCl_3$) δ 7.66 (2H, d, *J* = 7.1 Hz), 7.64 (2H, d, *J* = 7.4 Hz), 7.46–7.33 (6H, m), 5.15 (1H, s), 3.92 (1H, dd, *J* = 11.1, 4.5 Hz), 3.84 (1H, td, *J* = 10.3, 4.1 Hz), 3.81–3.72 (3H, m), 3.66 (1H, app. t, *J* = 9.5 Hz), 3.48 (1H, app. td, *J* = 8.5, 2.9 Hz), 3.37 (1H, app. td, *J* = 10.3, 4.7 Hz), 3.24 (1H, dd, *J* = 9.5, 4.9 Hz), 3.10 (1H, app. td, *J* = 9.8, 3.2 Hz), 2.69–2.59 (1H, m), 2.44 (1H, app. dt, *J* = 12.3, 5.1 Hz), 2.23 (1H, app. p, *J* = 7.2 Hz), 2.14–2.06 (1H, m), 1.98–1.78 (4H, m), 1.74–1.59 (4H, m), 1.28 (3H, d, *J* = 7.5 Hz), 1.18 (9H, s), 1.09 (3H, d, *J* = 7.1 Hz), 1.05 (3H, d, *J* = 6.8 Hz), 1.04 (9H, s), 0.90 (9H, s), 0.89 (3H, d, *J* = 7.0 Hz), 0.07 (3H, s), 0.03 (3H, s). ^{13}C NMR (126 MHz, $CDCl_3$) δ 200.8, 178.3, 173.5, 135.5, 134.1, 133.8, 129.6, 127.7, 127.6, 104.8, 83.3, 81.5, 80.9, 80.0, 79.9, 76.0, 67.1, 65.9, 60.1, 54.6, 43.7, 42.5, 38.9, 38.2, 37.1, 35.7, 27.9, 27.2, 26.9, 25.9, 19.3, 19.2, 17.9, 15.3, 15.0, 13.8, −4.0, −4.8. HRMS (ESI+) *m/z*: $[M + Na]^+$ Calcd for $C_{50}H_{78}NaO_8Si_2$ 885.5127; Found 885.5118.

Ketone S6.

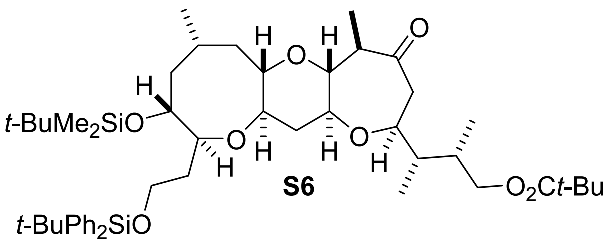

S6

To a solution of enone **27** (20 mg, 0.023 mmol) in degassed MeOH (1.0 mL) was added Pd-C (10 mg, 10 % by weight). The mixture was stirred vigorously under an atmosphere of hydrogen at room temperature for 48 h. The reaction mixture was filtered through a pad of celite and the filtrate was concentrated. The residue was purified by flash column chromatography on silica gel (pet. ether-diethyl ether, 5:1) to give the cyclic ketone **S6** (15.0 mg, 75%) as a colourless oil. R_f = 0.50 (pet. ether-diethyl ether, 4:1). $[\alpha]_D^{25}$ −20.5 (*c* = 0.17, $CHCl_3$). ν_{max} (film) 2954, 2927, 2854, 1729, 1713, 836, 774, 735 cm^{-1}. ^1H NMR (500 MHz, $CDCl_3$) δ 7.66 (4H, m), 7.43–7.33 (6H, m), 3.94–3.80 (4H, m), 3.76–3.61 (3H, m), 3.51–3.40 (2H, m), 3.23 (1H, ddd, *J* = 11.6, 8.8, 4.2 Hz), 3.09 (1H, ddd, *J* = 10.1, 9.9, 3.6 Hz), 2.98 (1H, app. dq, *J* = 10.6, 6.4 Hz), 2.69–2.54 (3H, m), 2.50 (1H, app. dt, *J* =

12.1, 4.6 Hz), 2.12–2.06 (1H, m) 1.96–1.75 (4H, m), 1.73 (1H, dd, J = 9.1, 6.0 Hz), 1.64–1.56 (2H, m), 1.46 (1H, app. q, J = 11.4 Hz), 1.19 (9H, s), 1.12 (3H, d, J = 6.4 Hz), 1.05 (12H, m), 0.89 (9H, s), 0.78 (3H, d, J = 6.9 Hz), 0.77 (3H, d, J = 6.9 Hz), 0.06 (3H, s), 0.01 (3H, s). ^{13}C NMR (126 MHz, $CDCl_3$) δ 210.0, 178.6, 135.7, 135.5, 134.4, 134.0, 129.8, 129.7, 127.8, 127.8, 84.2, 82.1, 81.9, 81.6, 81.1, 79.9, 76.3, 67.5, 60.2, 50.4, 50.0, 44.7, 39.4, 39.0, 38.9, 37.1, 33.3, 29.9, 27.8, 27.6, 27.4, 27.0, 26.0, 19.5, 18.0, 12.4, 11.6, 9.6, −3.9, −4.6. HRMS (ESI+) m/z: [M + Na]$^+$ Calcd for $C_{50}H_{80}NaO_8Si_2$ 887.5284; Found 887.5260.

Enol Triflate 28.

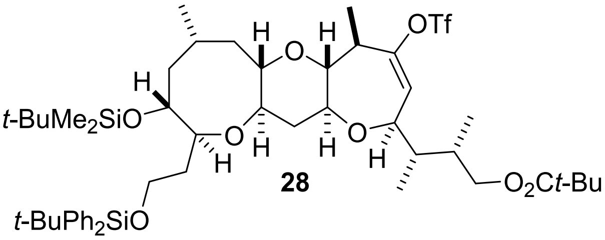

28

Sodium bis(trimethylsilyl)amide (0.2 mL of a 1 M solution in THF, 0.2 mmol) was added to a stirred solution of ketone **S6** (17 mg, 0.02 mmol) and *N*,*N*-bis(trifluoromethylsulfonyl)aniline (25 mg, 0.07 mmol) in THF (2.5 mL) at −78 °C under argon. After 30 min, the reaction was quenched by addition of water and the mixture was diluted with ethyl acetate. The organic phase was washed with brine, dried (magnesium sulfate) and concentrated. The residue was purified by flash column chromatography on silica gel (pet. ether-diethyl ether, 10:1) to give the enol triflate **28** as colourless oil (14 mg, 72%). R_f = 0.50 (pet. ether-diethyl ether, 9:1). $[\alpha]_D^{25}$ −48 (c = 0.2, $CHCl_3$). ν_{max} (film) 3439, 2920, 2851, 1740, 727 cm^{-1}. ^{1}H NMR (400 MHz, C_6D_6) δ 7.82–7.77 (4H, m), 7.35–7.22 (6H, m), 5.65 (1H, dd, J = 3.2, 1.4 Hz), 4.02–3.89 (4H, m), 3.79 (1H, ddd, J = 10.0, 6.2, 2.9 Hz), 3.70 (1H, ddd, J = 10.4, 8.2, 2.2 Hz), 3.45 (1H, app. td, J = 8.5, 2.8 Hz), 3.30 (1H, ddd, J = 11.2, 9.1, 4.7 Hz), 3.07 (1H, ddd, J = 11.2, 9.0, 4.8 Hz), 2.98 (1H, app. td, J = 9.7, 3.3 Hz), 2.86 (1H, dd, J = 9.1, 6.8 Hz), 2.81–2.71 (1H, m), 2.42 (1H, app. dt, J = 12.2, 4.8 Hz), 2.23–2.13 (1H, m), 1.93–1.75 (4H, m), 1.72–1.60 (2H, m), 1.49 (1H, app. q, J = 11.5 Hz), 1.43–1.35 (2H, m), 1.33 (3H, d, J = 7.0 Hz), 1.18 (9H, s), 1.13 (9H, s), 1.00 (3H, d, J = 6.9 Hz), 0.98 (9H, s), 0.81 (3H, d, J = 7.0 Hz), 0.80 (3H, d, J = 7.0 Hz), 0.03 (3H, s), 0.00 (3H, s). ^{13}C NMR (101 MHz, C_6D_6) δ 177.9, 151.5, 135.7, 135.6, 134.3, 129.7, 129.7, 128.3, 128.3, 127.9, 127.8, 124.9, 83.2, 80.9, 80.6, 80.3, 77.3, 76.9, 76.2, 67.3, 60.4, 44.8, 44.0, 42.3, 39.1, 39.1, 38.6, 37.3, 34.8, 27.7, 27.0, 26.9, 26.8, 25.8, 19.2, 17.8, 17.0, 13.8, 10.6, −4.2, −5.0. HRMS (ESI+) m/z: [M + H]$^+$ Calcd for $C_{51}H_{80}F_3O_{10}SSi_2$ 997.4957; Found 997.4968.

Tricyclic I–K Diol 29.

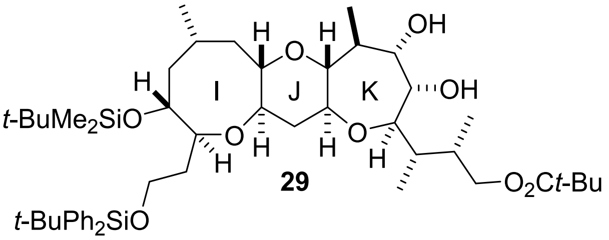

29

To a solution of the enol triflate **28** (10 mg, 0.010 mmol) in DMF (0.63 mL) was added triethylamine (0.042 mL, 0.30 mmol), formic acid (8 μL, 0.20 mmol) and dichlorobis(triphenylphosphine)-palladium(II) (2.8 mg, 0.004 mmol). The yellow solution was heated in a sealed tube to 60 °C (oil bath). The solution gradually turned to black and after 4 h TLC indicated that the reaction was complete. The mixture was cooled to room temperature and poured into water (5 mL). The mixture was extracted with diethyl ether (4 × 2 mL) and the combined organic extracts were washed with brine, dried (magnesium sulfate) and concentrated. The residue filtered through short pad of silica to yield the alkene as colourless oil, which was used in next step without further purification.

To the solution of the alkene in a 50% aqueous *t*-butanol (1.2 mL) at 0 °C was added a mixture of AD-mix-β (150 mg) and methylsulfonamide (1.0 mg, 0.011 mmol) and the mixture was stirred overnight. The reaction was quenched by the addition of sodium sulfite (150 mg) and the resulting mixture was extracted with ethyl acetate (1.5 mL). The organic extracts washed with saturated aqueous sodium bicarbonate solution (2.0 mL) and then concentrated under reduced pressure. The residue was purified by flash column chromatography on short column of silica gel (pet. ether-ethyl acetate, 1:1) to give the I–K diol **29** as colourless oil (4.5 mg, 51% over two steps). R_f = 0.20 (pet. ether-ethyl acetate, 7:3). $[\alpha]_D^{25}$ −17 (*c* = 0.1, $CHCl_3$). ν_{max} (film) 3515, 2956, 2854, 1733, 798 cm^{-1}. ^1H NMR (500 MHz, C_6D_6) δ 7.87–7.72 (4H, m), 7.37–7.26 (6H, m), 4.14–4.02 (3H, m), 3.86 (1H, ddd, *J* = 10.0, 6.5, 3.2 Hz), 3.80 (1H, app. dt, *J* = 5.9, 3.1 Hz), 3.67 (1H, ddd, *J* = 10.6, 8.1, 2.2 Hz), 3.53–3.45 (3H, m), 3.38 (1H, ddd, *J* = 10.9, 9.2, 4.9 Hz), 3.27 (2H, ddd, *J* = 11.5, 9.2, 4.4 Hz), 3.13 (1H, ddd, *J* = 10.0, 9.8, 3.8 Hz), 2.63 (1H, app. t, *J* = 9.2 Hz), 2.55 (1H, app. dt, *J* = 12.0, 4.6 Hz), 2.30–2.18 (2H, m), 2.06–2.00 (2H, m), 1.98–1.91 (2H, m), 1.88–1.67 (4H, m), 1.61–1.52 (1H, m), 1.47–1.38 (1H, m), 1.22 (9H, s), 1.22–1.17 (6H, m), 1.01 (9H, s), 0.96 (3H, d, *J* = 7.0 Hz), 0.94 (3H, d, *J* = 6.9 Hz), 0.30 (9H, s), 0.06 (3H, s), 0.04 (3H, s). ^{13}C NMR (151 MHz, C_6D_6) δ 177.9, 141.5, 136.0, 136.0, 134.7, 134.5, 130.0, 128.1, 127.9, 85.7, 84.5, 83.7, 81.7, 80.9, 80.3, 77.1, 76.7, 73.7, 67.9, 60.9, 45.0, 40.0, 39.0, 38.8, 38.0, 37.8, 35.3, 32.4, 27.5, 27.2, 26.2, 23.1, 19.6, 18.2, 14.9, 14.4, 13.8, 10.6, −3.9, −4.6. HRMS (ESI+) *m*/*z*: $[M + H]^+$ Calcd for $C_{50}H_{83}O_9Si_2$ 883.5570; Found 883.5580.

References

1. Popadynec, M.; Gibbard, H.; Clark, J. S. Bidirectional Synthesis of the IJK Fragment of Ciguatoxin CTX3C by Sequential Double Ring-Closing Metathesis and Tsuji−Trost Allylation. *Org. Lett*. **2020**, *22*, 3734–3738.
2. McCasland, G. E.; Proskow, S. The Conditions for Optical Inactivity. Synthesis of an Image-Superposable Molecule which Contains No Plane or Center of Symmetry. *J. Am. Chem. Soc.* **1956**, *78*, 5646–5652.
3. Lu, C.-D.; Zakarian, A. Synthesis of (2*R*,3*R*)-2,3-Dimethyl-1,4-Butanediol by Oxidative Homocoupling of (4*S*)-Isopropyl-3-Propionyl-2-Oxazolidinone. *Org. Synth.* **2008**, *85*, 158–171.

^{1}H and ^{13}C NMR Spectra for New Compounds

^{1}H NMR spectrum of **4** (400 MHz, $CDCl_3$)	26
^{13}C NMR spectrum of **4** (101 MHz, $CDCl_3$)	26
^{1}H NMR spectrum of **5** (400 MHz, $CDCl_3$)	27
^{13}C NMR spectrum of **5** (101 MHz, $CDCl_3$)	27
^{1}H NMR spectrum of **6** (400 MHz, $CDCl_3$)	28
^{13}C NMR spectrum of **6** (101 MHz, $CDCl_3$)	28
^{1}H NMR spectrum of **7** (400 MHz, $CDCl_3$)	29
^{13}C NMR spectrum of **7** (101 MHz, $CDCl_3$)	29
^{1}H NMR spectrum of **S1** (500 MHz, $CDCl_3$)	30
^{13}C NMR spectrum of **S1** (126 MHz, $CDCl_3$)	30
^{1}H NMR spectrum of **9** (400 MHz, $CDCl_3$)	31
^{13}C NMR spectrum of **9** (101 MHz, $CDCl_3$)	31
^{1}H NMR spectrum of **10** (400 MHz, $CDCl_3$)	32
^{13}C NMR spectrum of **10** (101 MHz, $CDCl_3$)	32
^{1}H NMR spectrum of **S2** (400 MHz, $CDCl_3$)	33
^{13}C NMR spectrum of **S2** (101 MHz, $CDCl_3$)	33
^{1}H NMR spectrum of **11** (400 MHz, $CDCl_3$)	34
^{13}C NMR spectrum of **11** (101 MHz, $CDCl_3$)	34
^{1}H NMR spectrum of **12** (400 MHz, $CDCl_3$)	35
^{13}C NMR spectrum of **12** (101 MHz, $CDCl_3$)	35
^{1}H NMR spectrum of **13** (400 MHz, $CDCl_3$)	36
^{13}C NMR spectrum of **13** (101 MHz, $CDCl_3$)	36
^{1}H NMR spectrum of **14** (400 MHz, $CDCl_3$)	37
^{13}C NMR spectrum of **14** (101 MHz, $CDCl_3$)	37
^{1}H NMR spectrum of **S3** (500 MHz, $CDCl_3$)	38
^{13}C NMR spectrum of **S3** (126 MHz, $CDCl_3$)	38
^{1}H NMR spectrum of **15** (400 MHz, $CDCl_3$)	39
^{13}C NMR spectrum of **15** (101 MHz, $CDCl_3$)	39
^{1}H NMR spectrum of **16** (400 MHz, C_6D_6)	40
^{13}C NMR spectrum of **16** (101 MHz, C_6D_6)	40
^{1}H NMR spectrum of **17** (400 MHz, $CDCl_3$)	41
^{13}C NMR spectrum of **17** (101 MHz, $CDCl_3$)	41
^{1}H NMR spectrum of **18** (500 MHz, $CDCl_3$)	42
^{13}C NMR spectrum of **18** (101 MHz, $CDCl_3$)	42
^{1}H NMR spectrum of **19** (400 MHz, $CDCl_3$)	43
^{13}C NMR spectrum of **19** (101 MHz, $CDCl_3$)	43
^{1}H NMR spectrum of **21** (500 MHz, $CDCl_3$)	44

^{13}C NMR spectrum of **21** (126 MHz, $CDCl_3$)	44
^{1}H NMR spectrum of **23** (500 MHz, $CDCl_3$)	45
^{13}C NMR spectrum of **23** (126 MHz, $CDCl_3$)	45
^{1}H NMR spectrum of **24** (400 MHz, $CDCl_3$)	46
^{13}C NMR spectrum of **24** (101 MHz, $CDCl_3$)	46
^{1}H NMR spectrum of **25** (400 MHz, C_6D_6)	47
^{13}C NMR spectrum of **25** (101 MHz, C_6D_6)	47
^{1}H NMR spectrum of **S4** (500 MHz, $CDCl_3$)	48
^{13}C NMR spectrum of **S4** (101MHz, $CDCl_3$)	48
^{1}H NMR spectrum of **26** (500 MHz, $CDCl_3$)	49
^{13}C NMR spectrum of **26** (101 MHz, $CDCl_3$)	49
^{1}H NMR spectrum of **S5** (500 MHz, $CDCl_3$)	50
^{13}C NMR spectrum of **S5**(101 MHz, $CDCl_3$)	50
^{1}H NMR spectrum of **27** (500 MHz, $CDCl_3$)	51
^{13}C NMR spectrum of **27** (126 MHz, $CDCl_3$)	51
^{1}H NMR spectrum of **S6** (500 MHz, $CDCl_3$)	52
^{13}C NMR spectrum of **S6** (126 MHz, $CDCl_3$)	52
^{1}H NMR spectrum of **28** (400 MHz, C_6D_6)	53
^{13}C NMR spectrum of **28** (101 MHz, C_6D_6)	53
^{1}H NMR spectrum of **29** (500 MHz, C_6D_6)	54
^{13}C NMR spectrum of **29** (151 MHz, C_6D_6)	54
NOESY spectrum of **S3** (500 MHz, $CDCl_3$)	55
NOESY spectrum of **15** (400 MHz, $CDCl_3$)	56
NOESY spectrum of **S6** (500 MHz, $CDCl_3$)	57
NOESY spectrum of **28** (400 MHz, C_6D_6)	58
NOESY spectrum of **29** (500 MHz, C_6D_6)	59

Enone 4.

^{1}H NMR (400 MHz, $CDCl_3$)

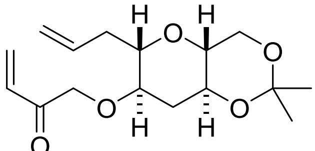

4

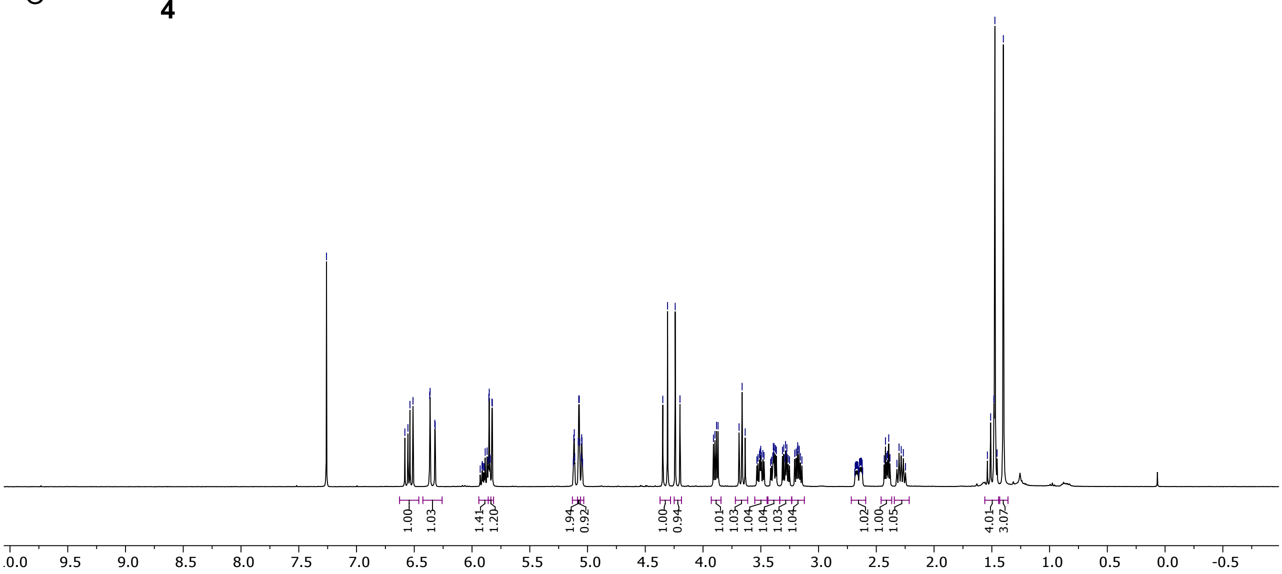

^{13}C NMR {^{1}H} (101 MHz, $CDCl_3$)

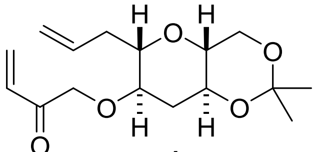

4

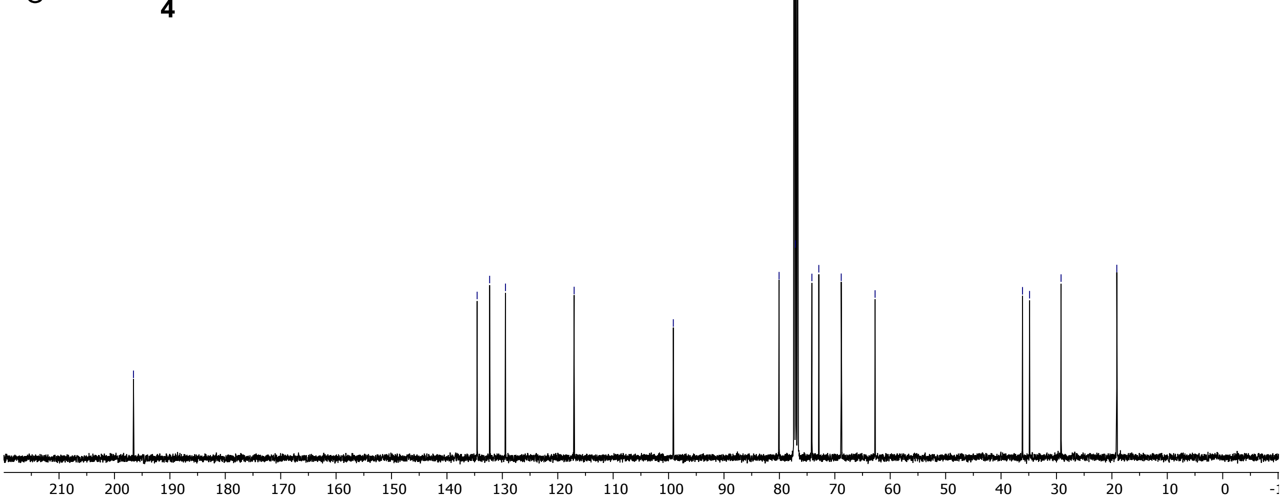

Allylic Alcohol 5.

^{1}H NMR (400 MHz, $CDCl_3$)

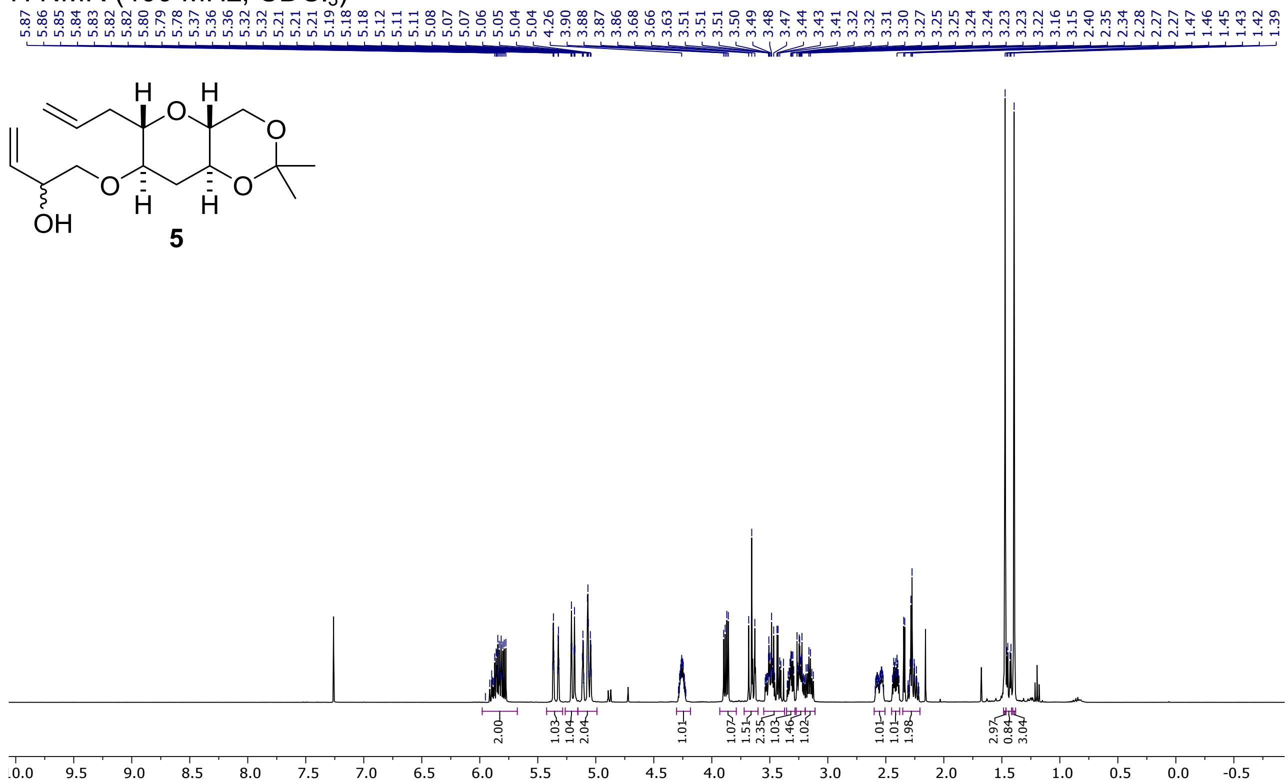

^{13}C NMR {^{1}H} (101 MHz, $CDCl_3$)

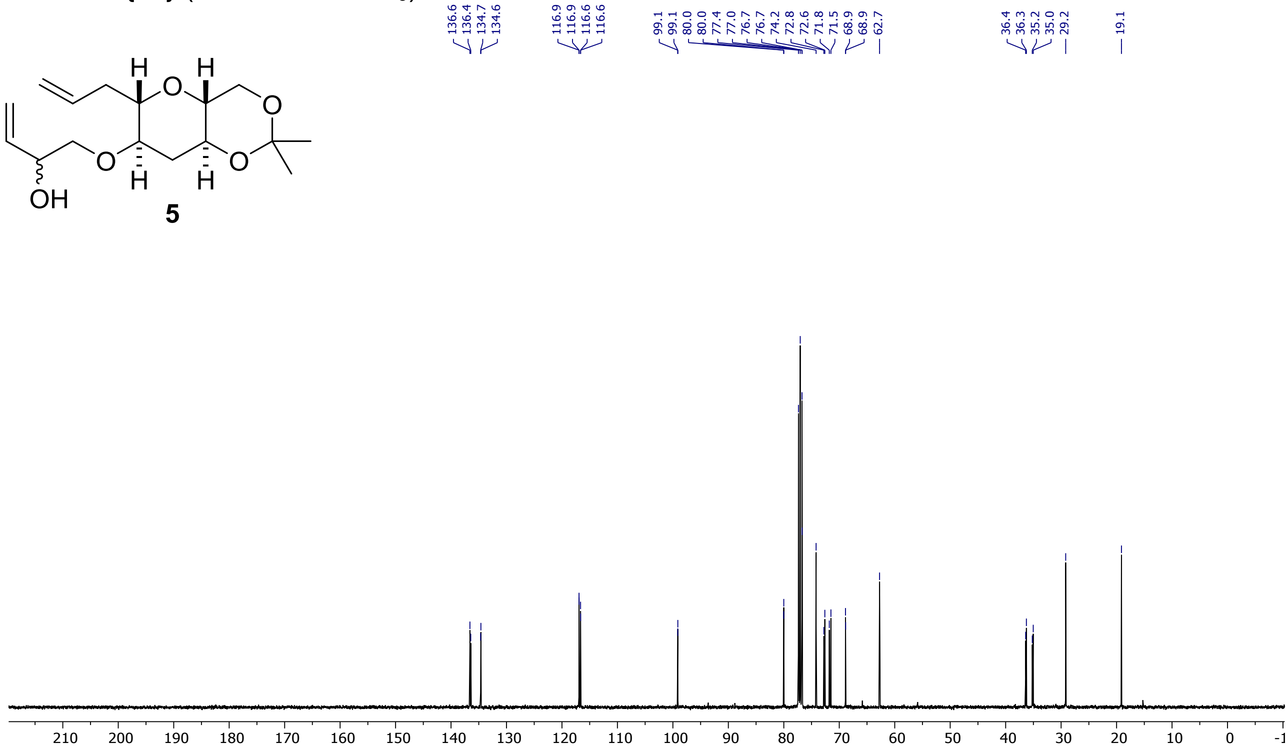

Enone 6.

^{1}H NMR (400 MHz, CDCl$_3$)

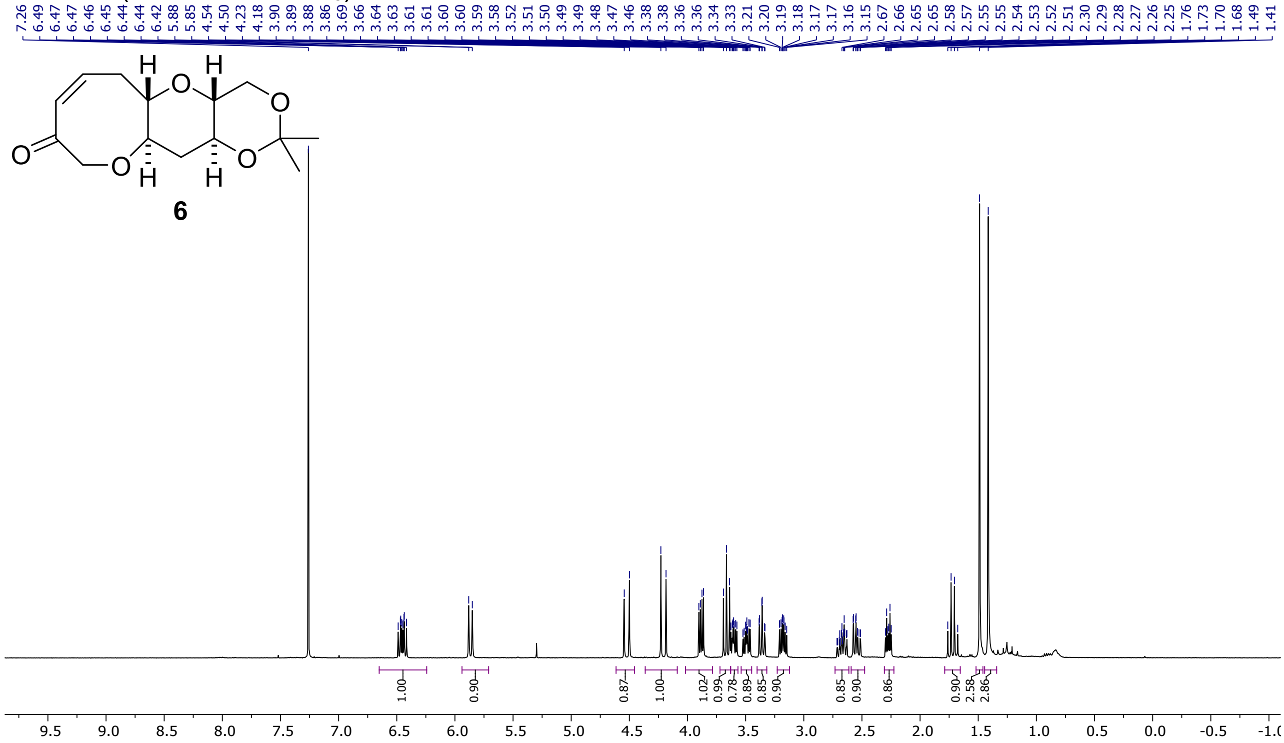

^{13}C NMR {^{1}H} (101 MHz, CDCl$_3$)

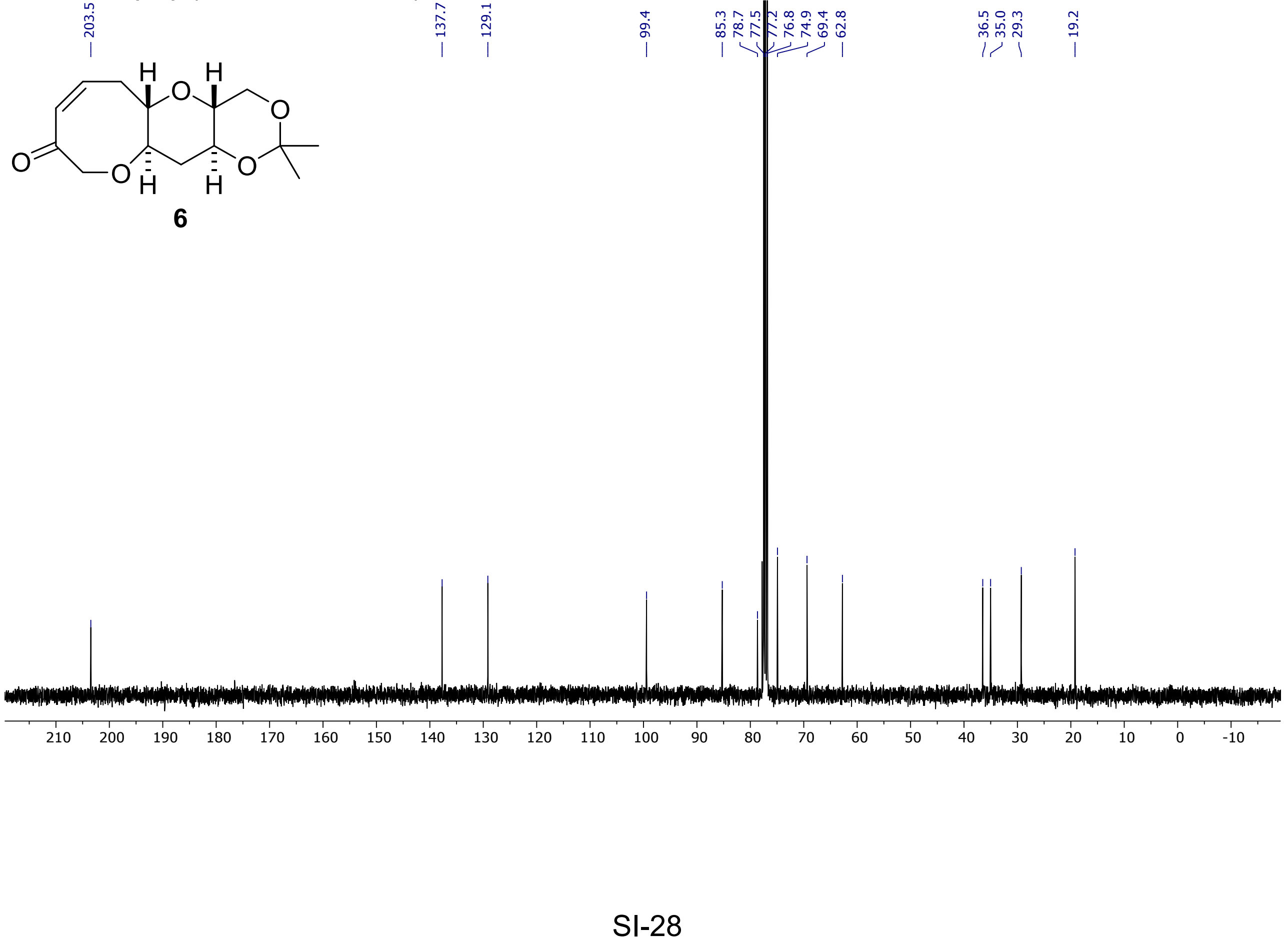

Enol carbonate 7.

^{1}H NMR (400 MHz, $CDCl_3$)

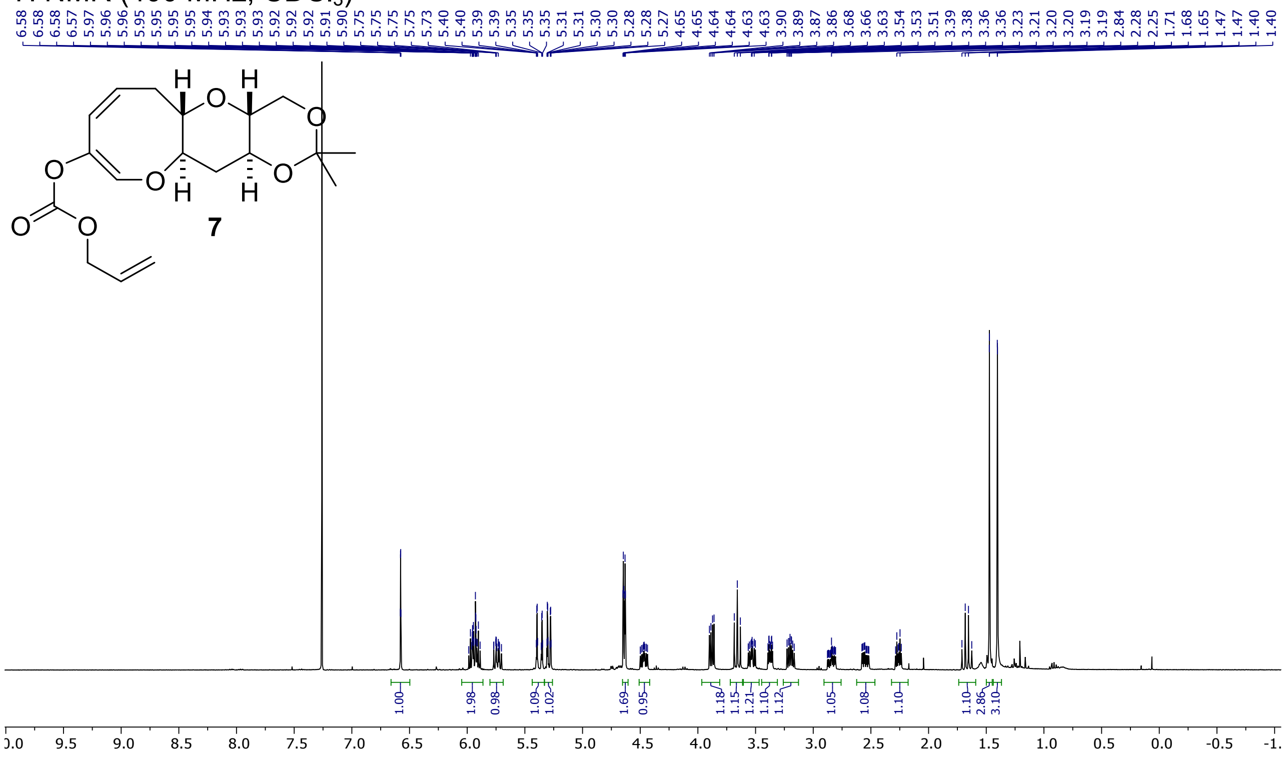

^{13}C NMR {^{1}H} (101 MHz, $CDCl_3$)

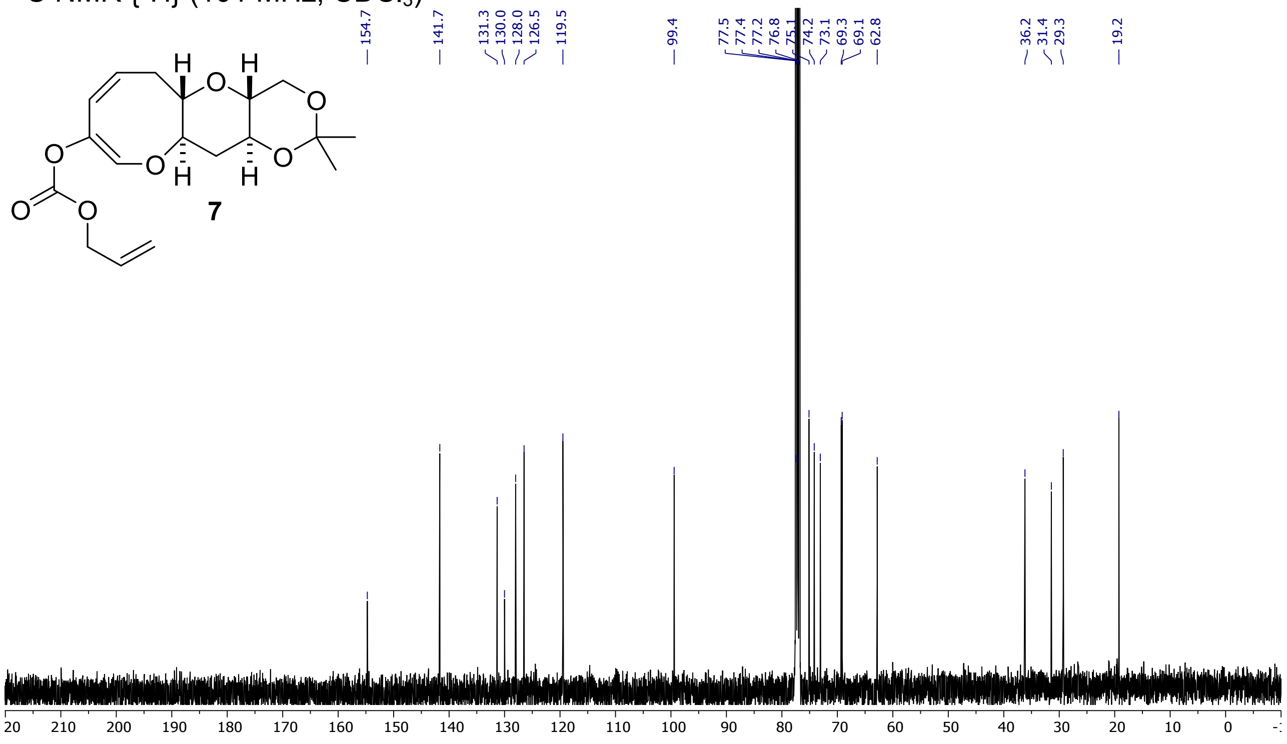

Enone S1.

^{1}H NMR (500 MHz, $CDCl_3$)

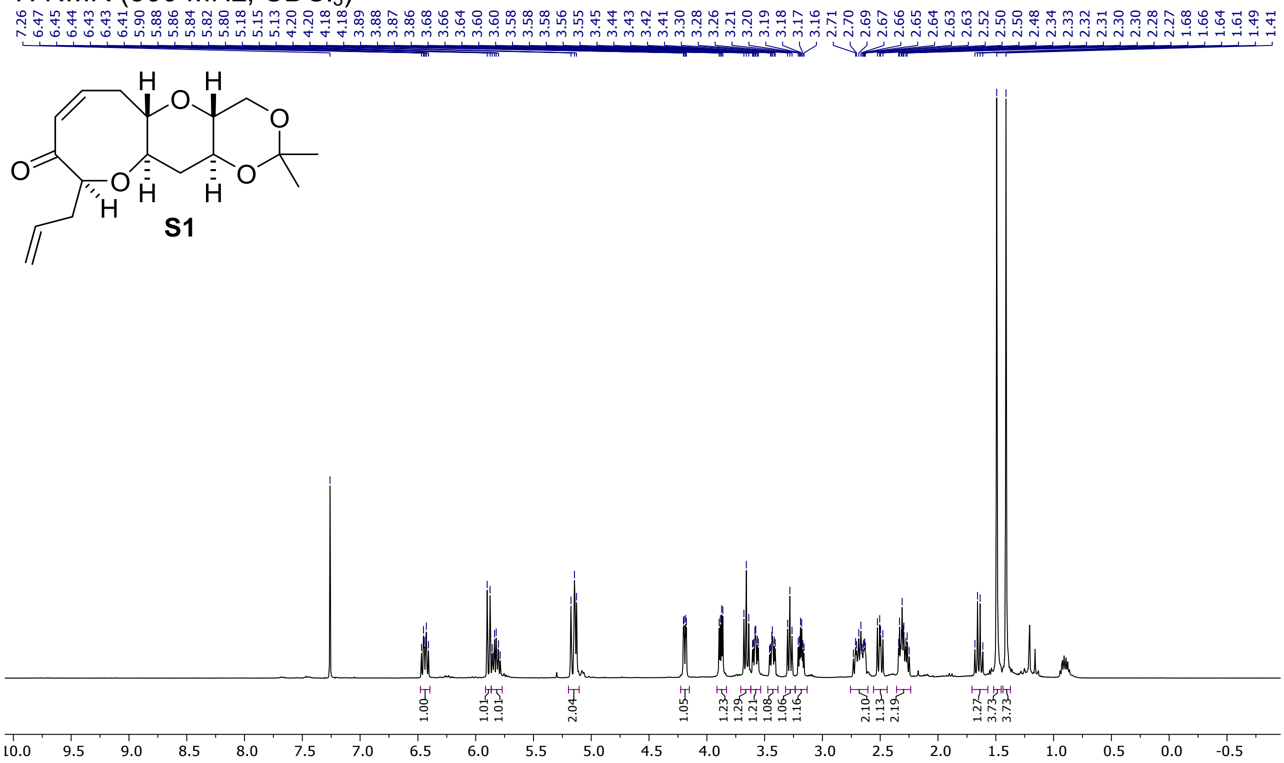

^{13}C NMR {^{1}H} (126 MHz, $CDCl_3$)

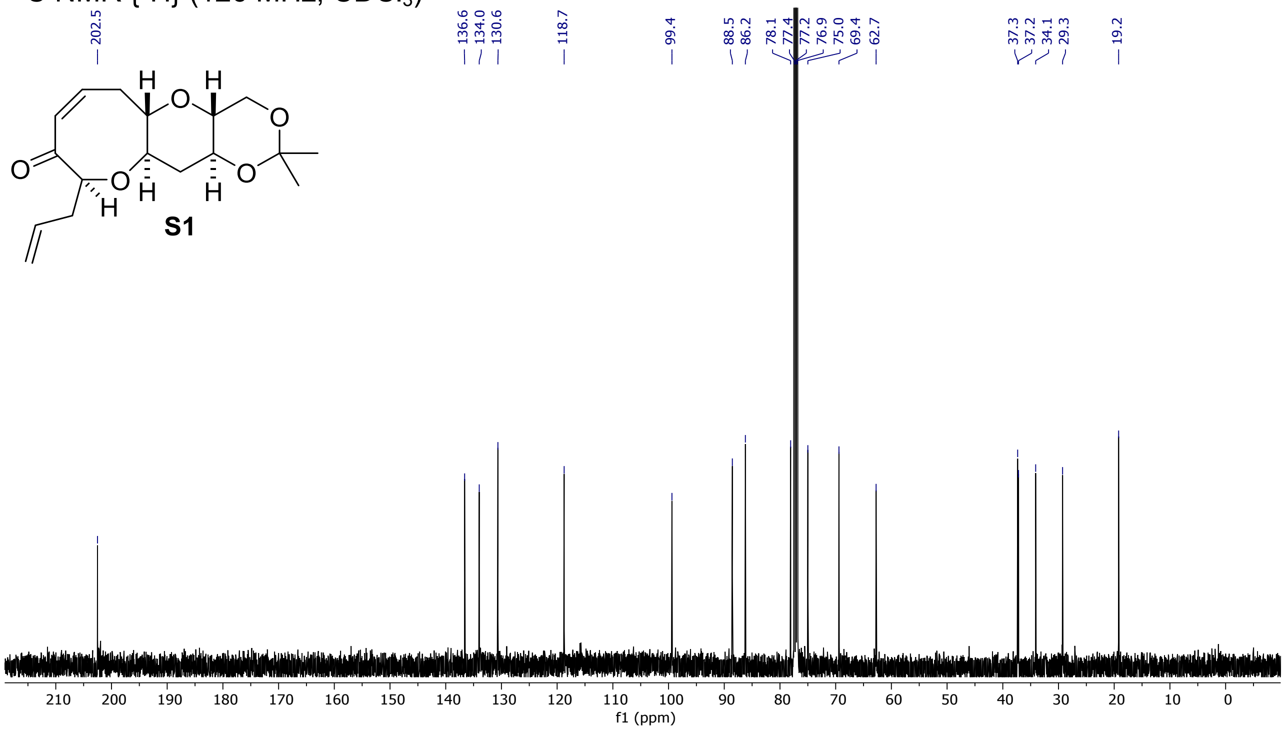

Ketone 9.

^{1}H NMR (400 MHz, $CDCl_3$)

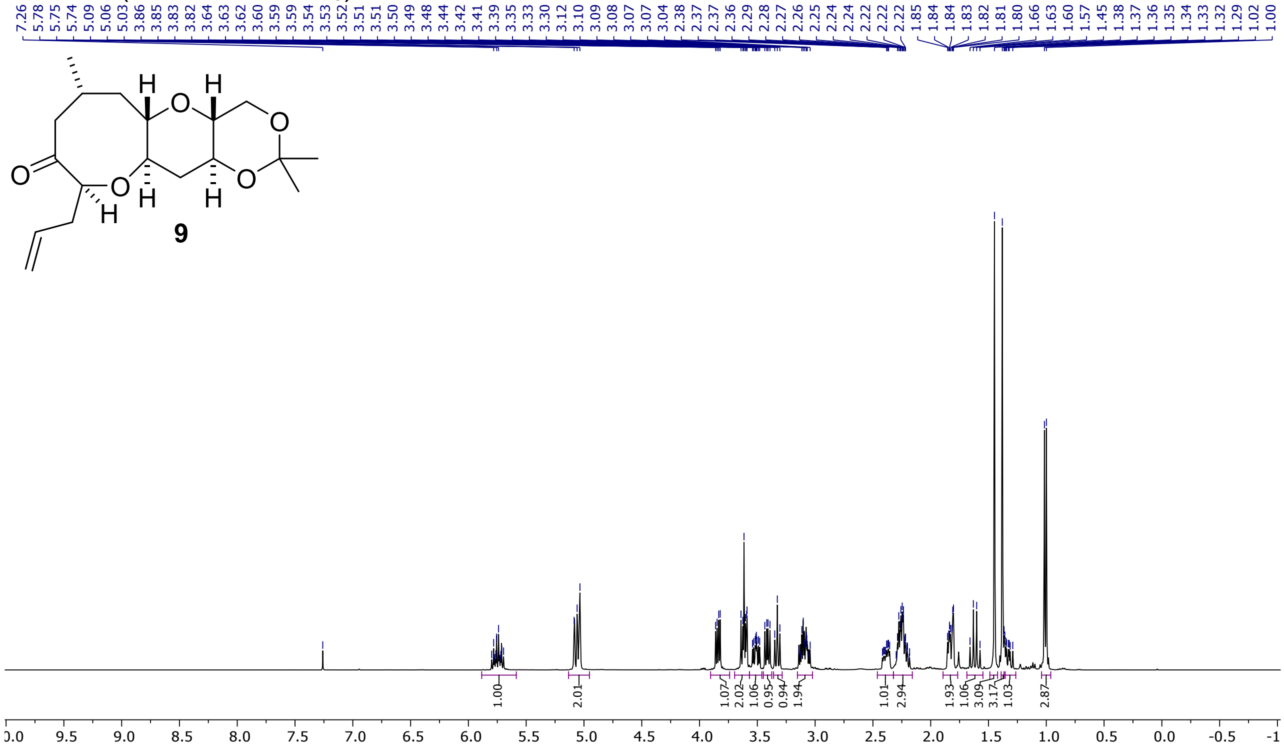

^{13}C NMR {^{1}H} (101 MHz, $CDCl_3$)

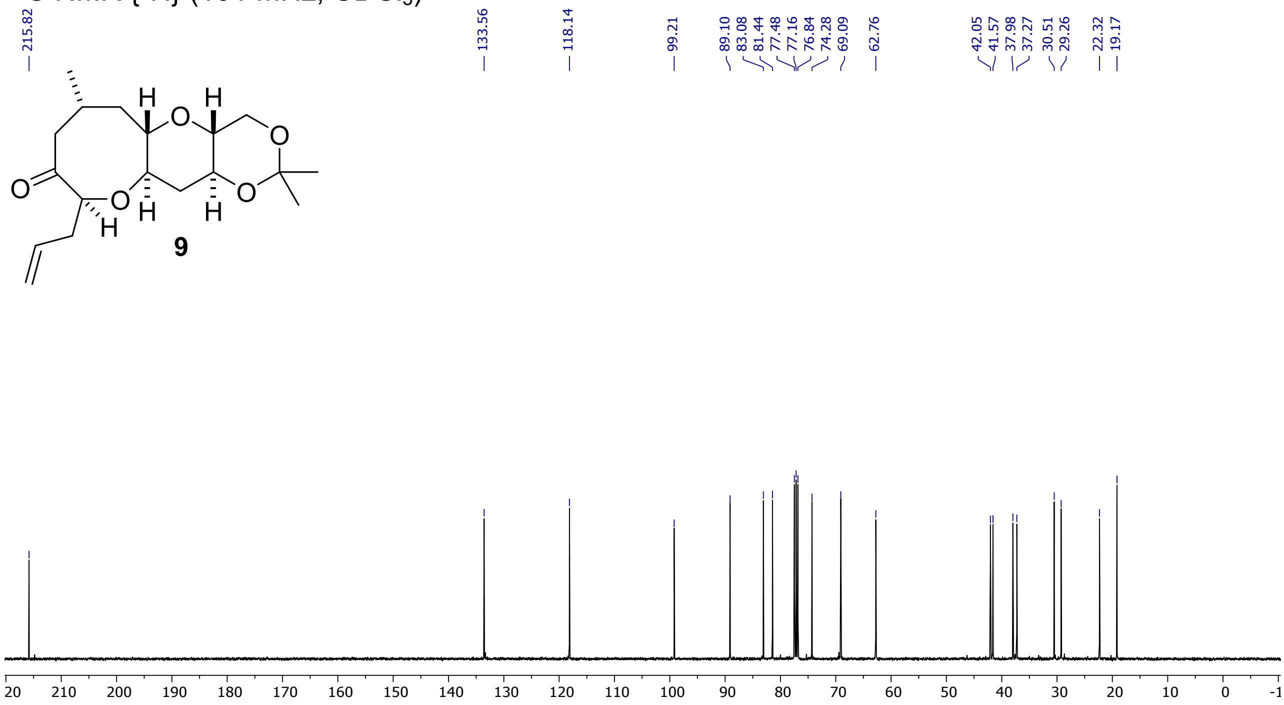

Alcohol 10.

^{1}H NMR (400 MHz, $CDCl_3$)

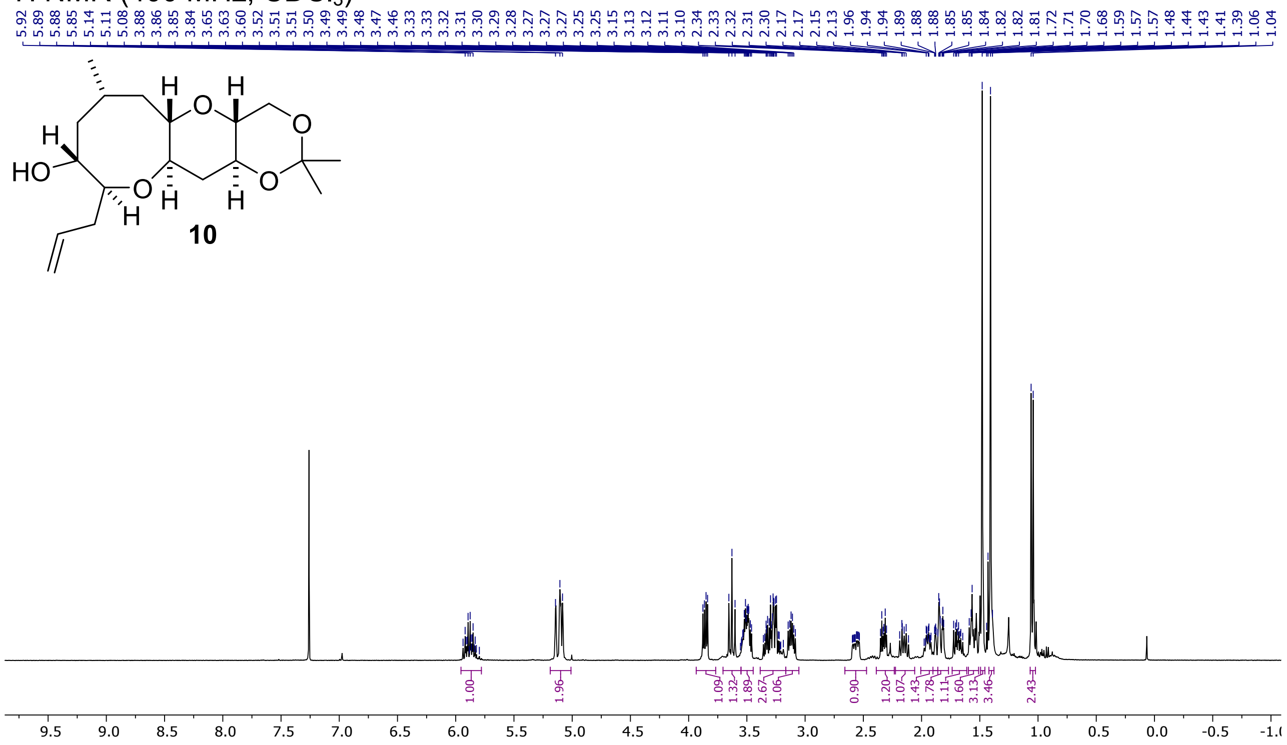

^{13}C NMR {^{1}H} (101 MHz, $CDCl_3$)

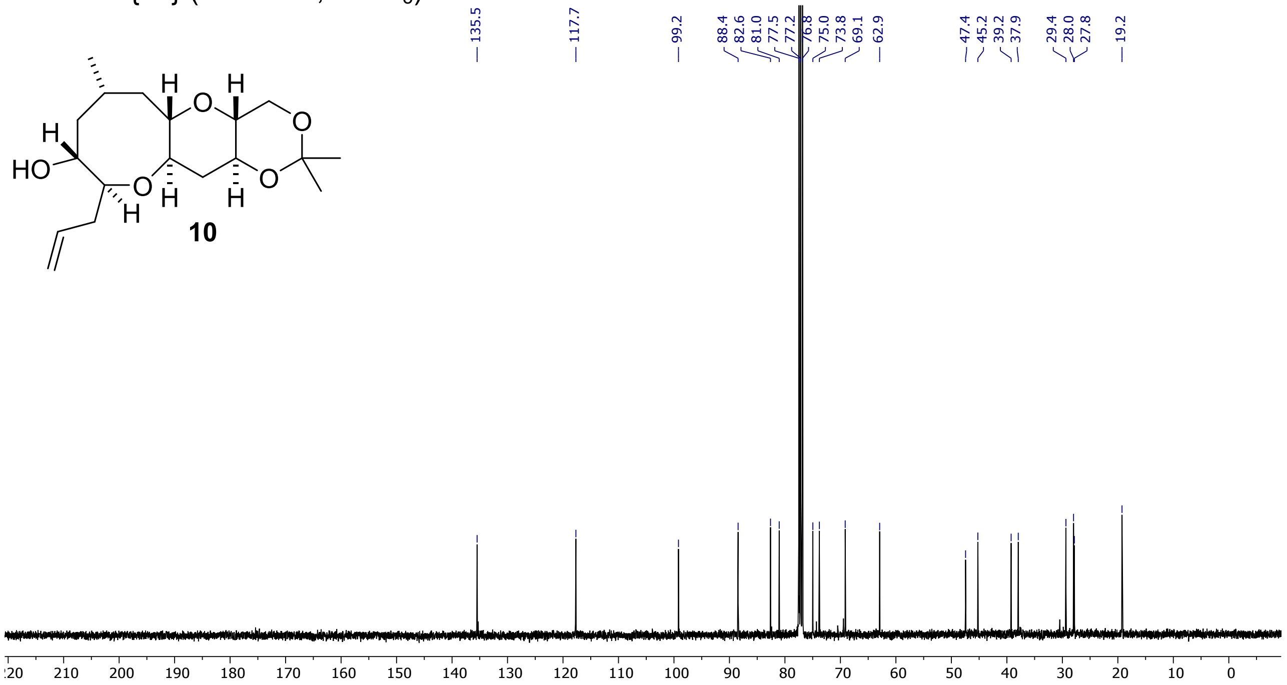

Silyl Ether S2

^{1}H NMR (400 MHz, $CDCl_3$)

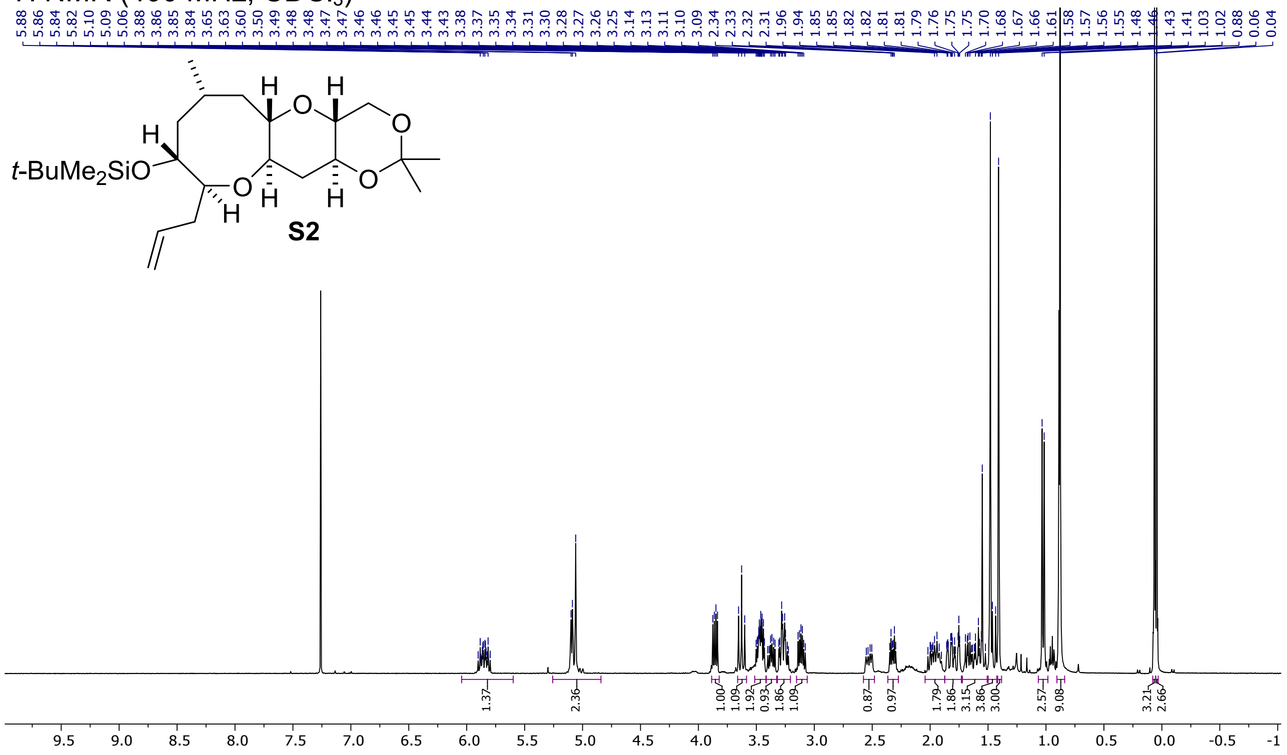

^{13}C NMR {^{1}H} (101 MHz, $CDCl_3$)

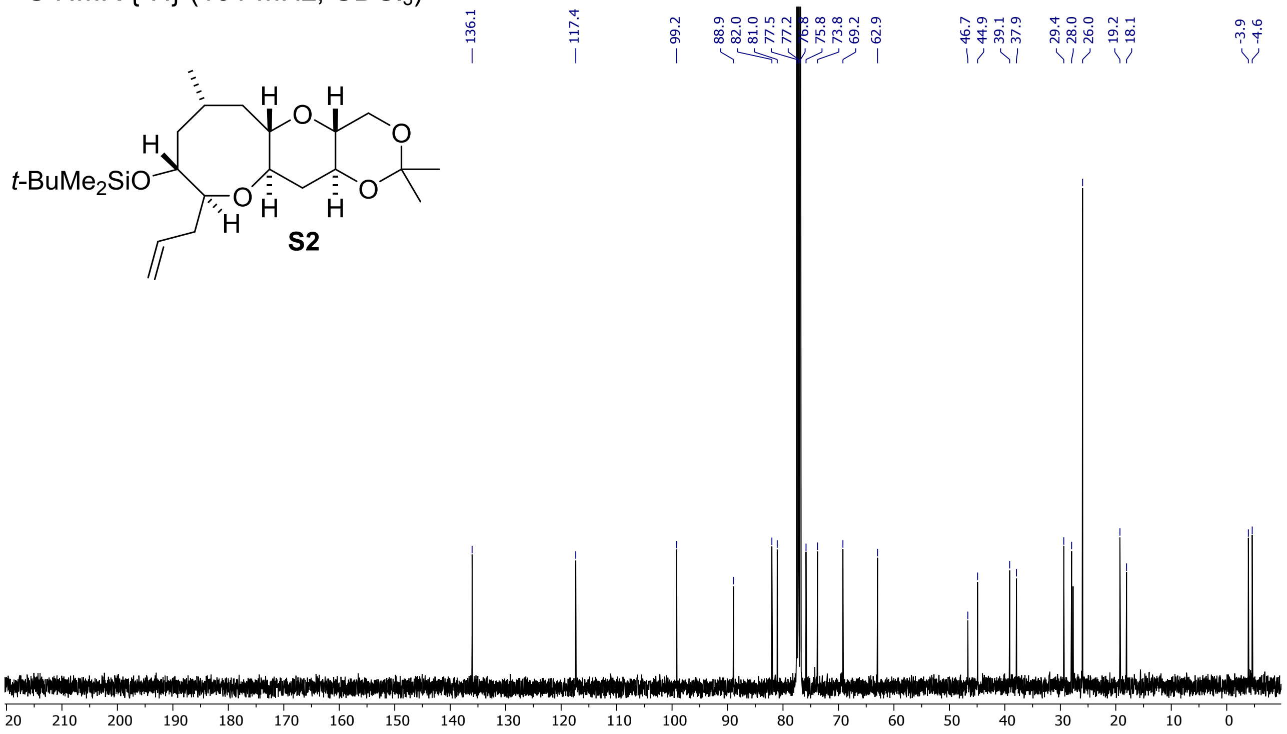

Diol 11.

^{1}H NMR (400 MHz, $CDCl_3$)

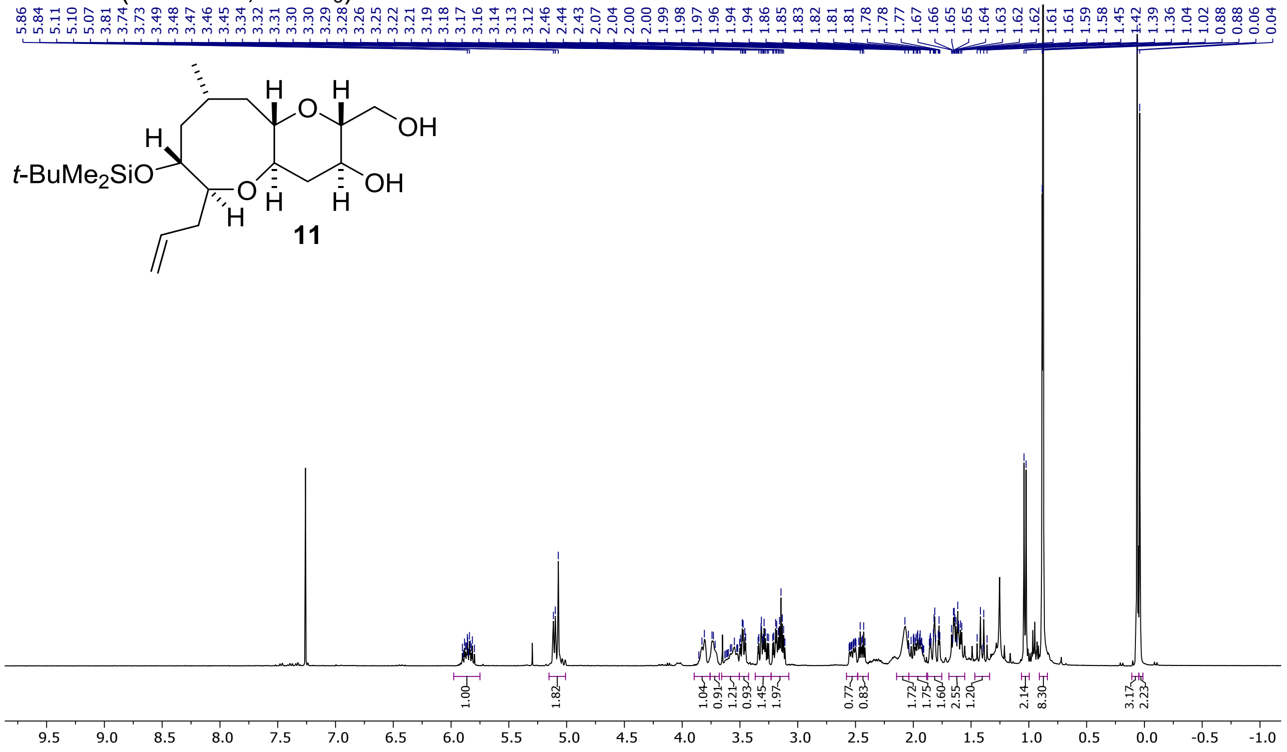

^{13}C NMR {^{1}H} (101 MHz, $CDCl_3$)

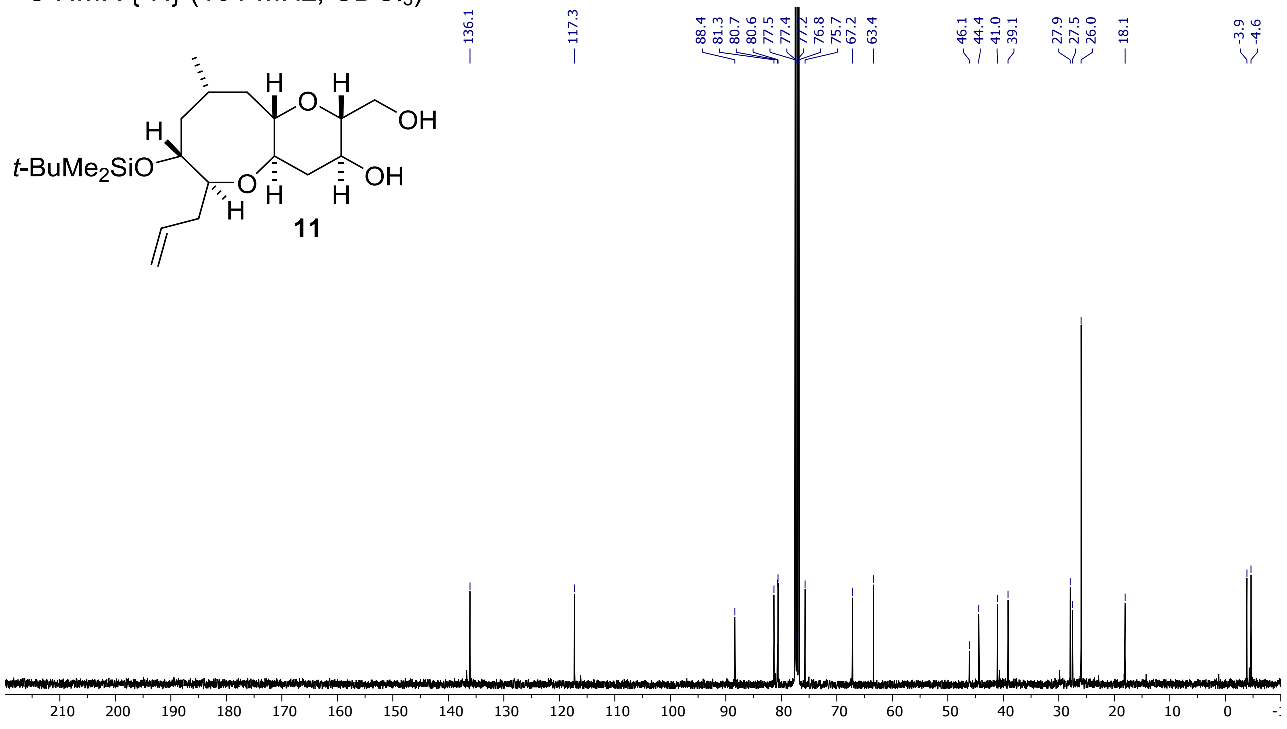

Nitrile 12.

^{1}H NMR (400 MHz, $CDCl_3$)

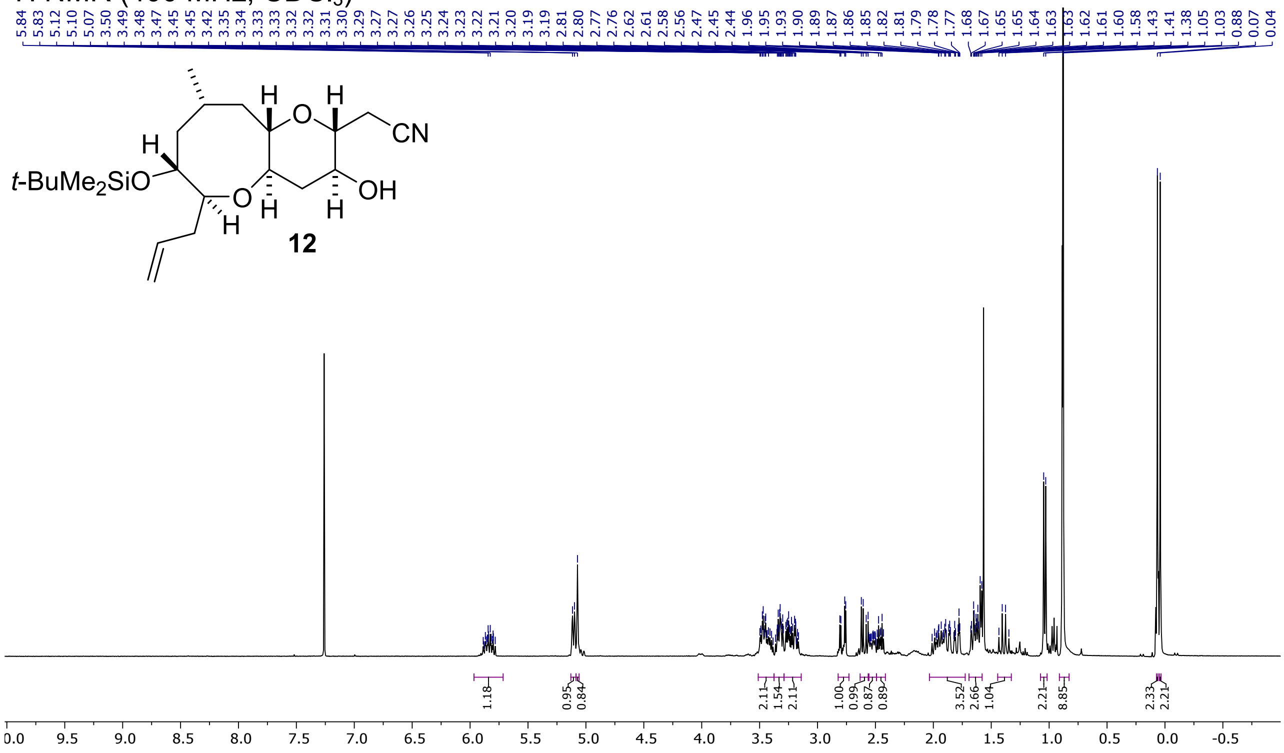

^{13}C NMR {^{1}H} (101 MHz, $CDCl_3$)

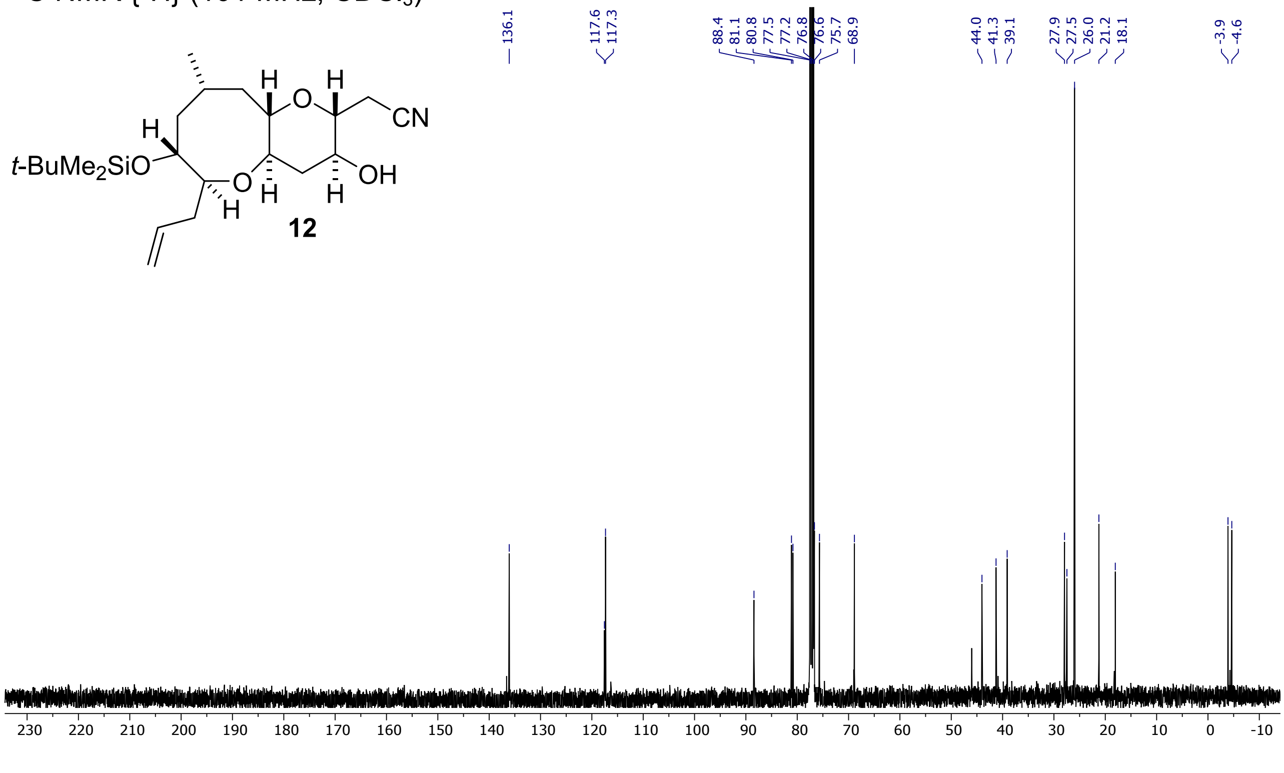

Aldehyde 13.

^{1}H NMR (400 MHz, $CDCl_3$)

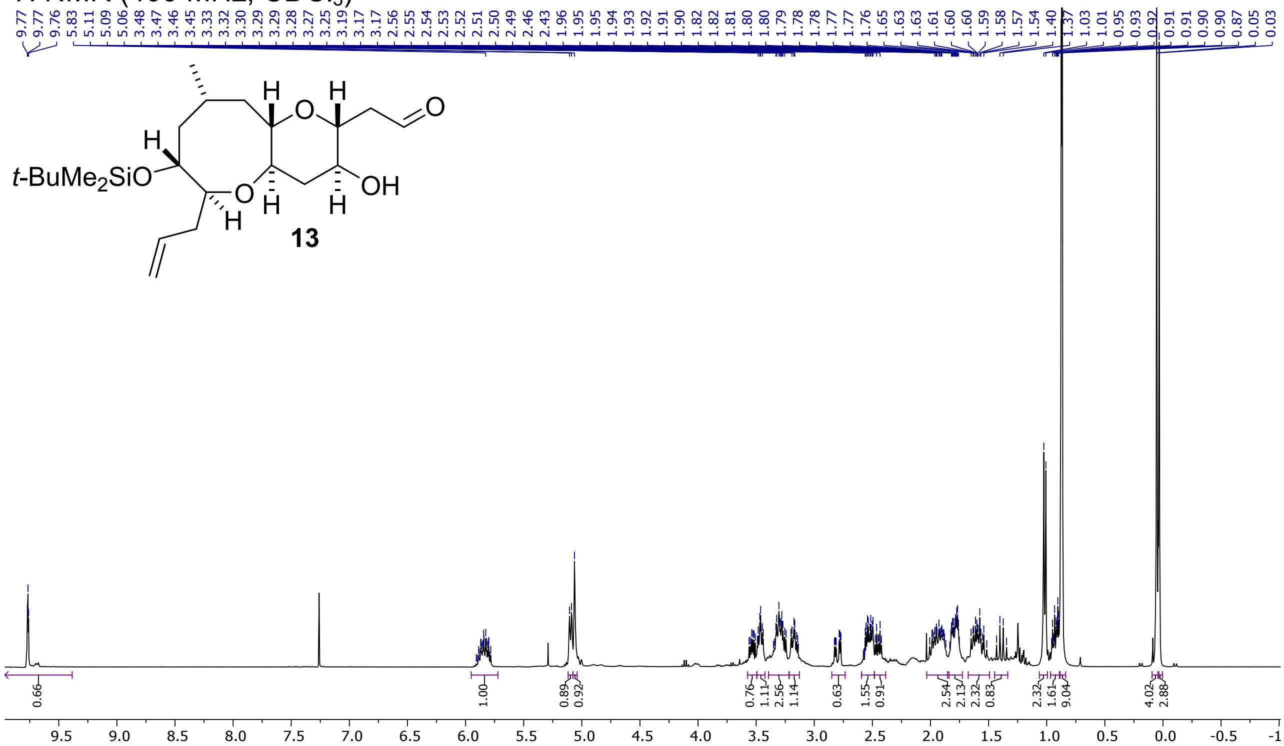

^{13}C NMR {^{1}H} (101 MHz, $CDCl_3$)

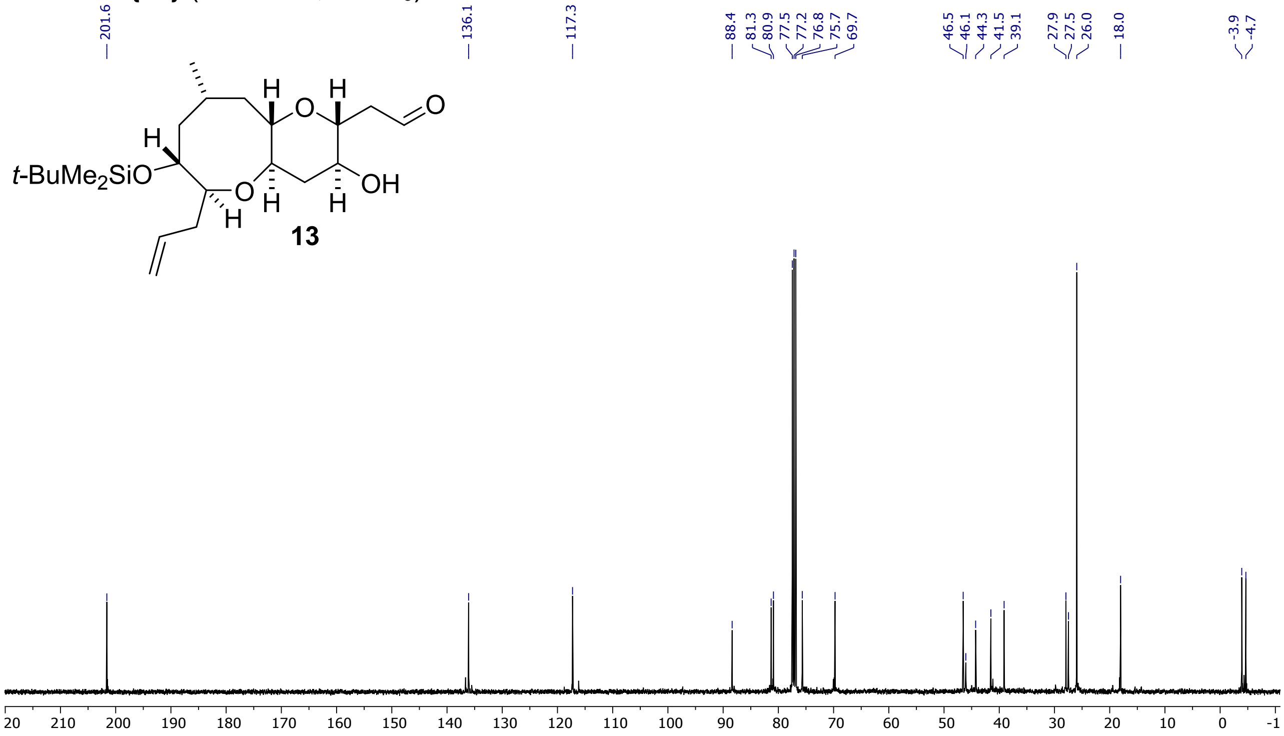

Lactone 14.

^{1}H NMR (400 MHz, $CDCl_3$)

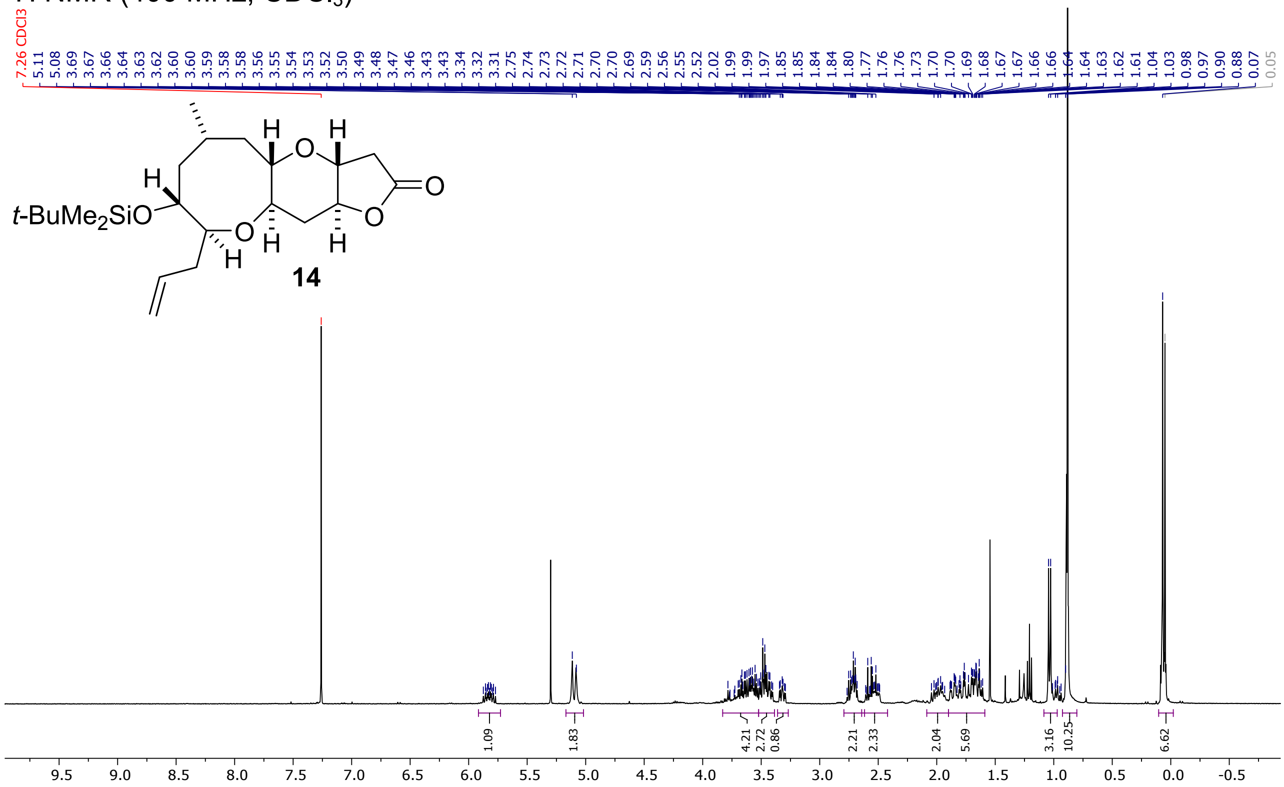

^{13}C NMR {^{1}H} (101 MHz, $CDCl_3$)

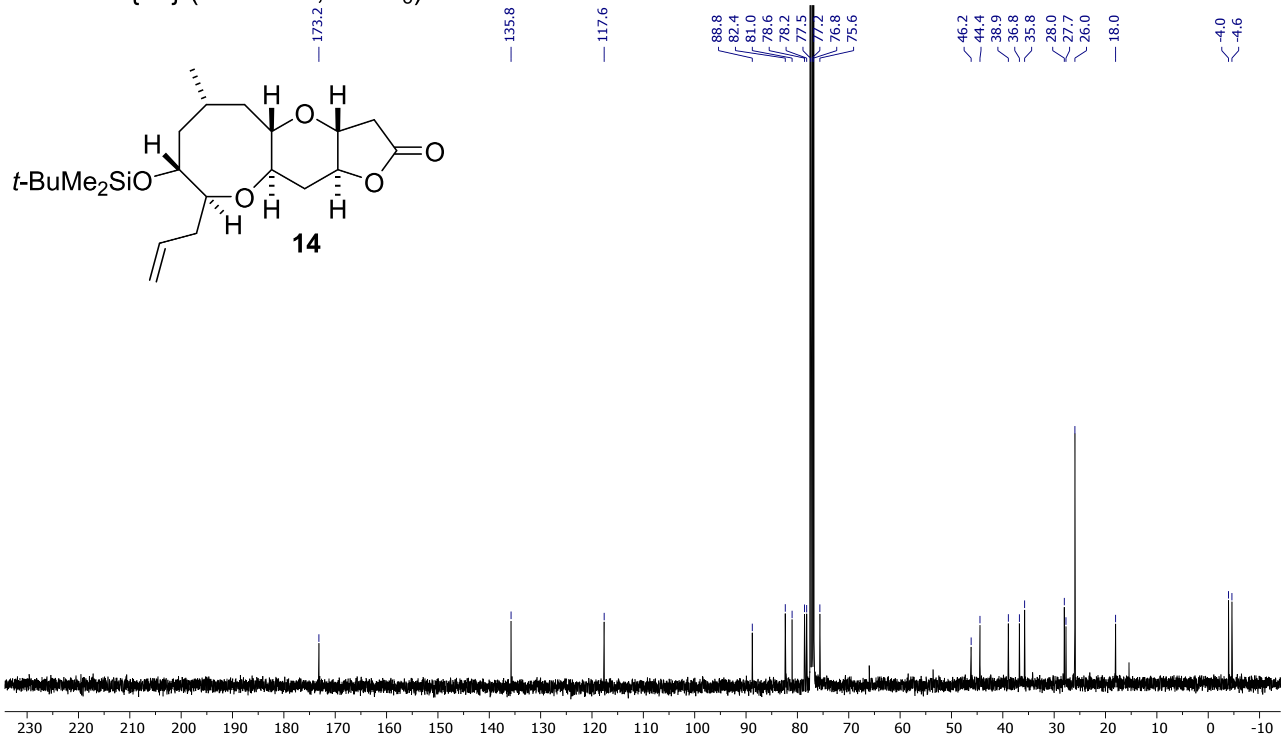

Lactone S3.

^{1}H NMR (500 MHz, $CDCl_3$)

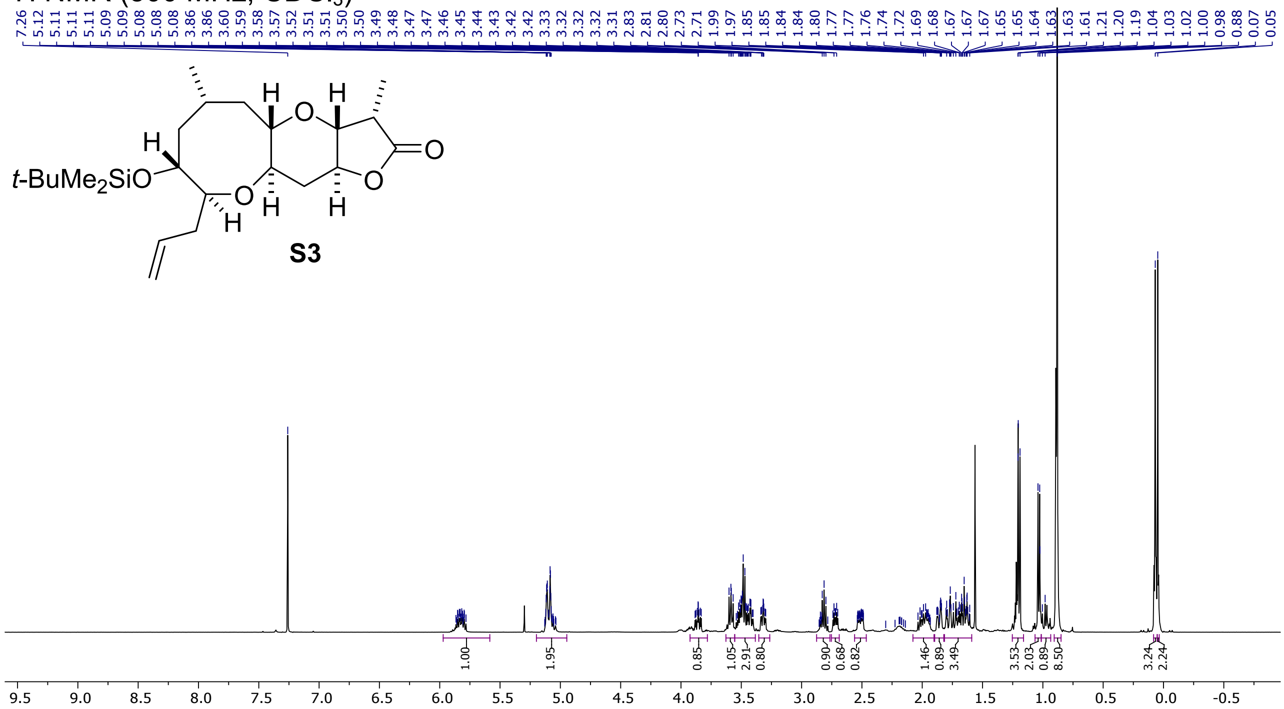

^{13}C NMR {^{1}H} (126 MHz, $CDCl_3$)

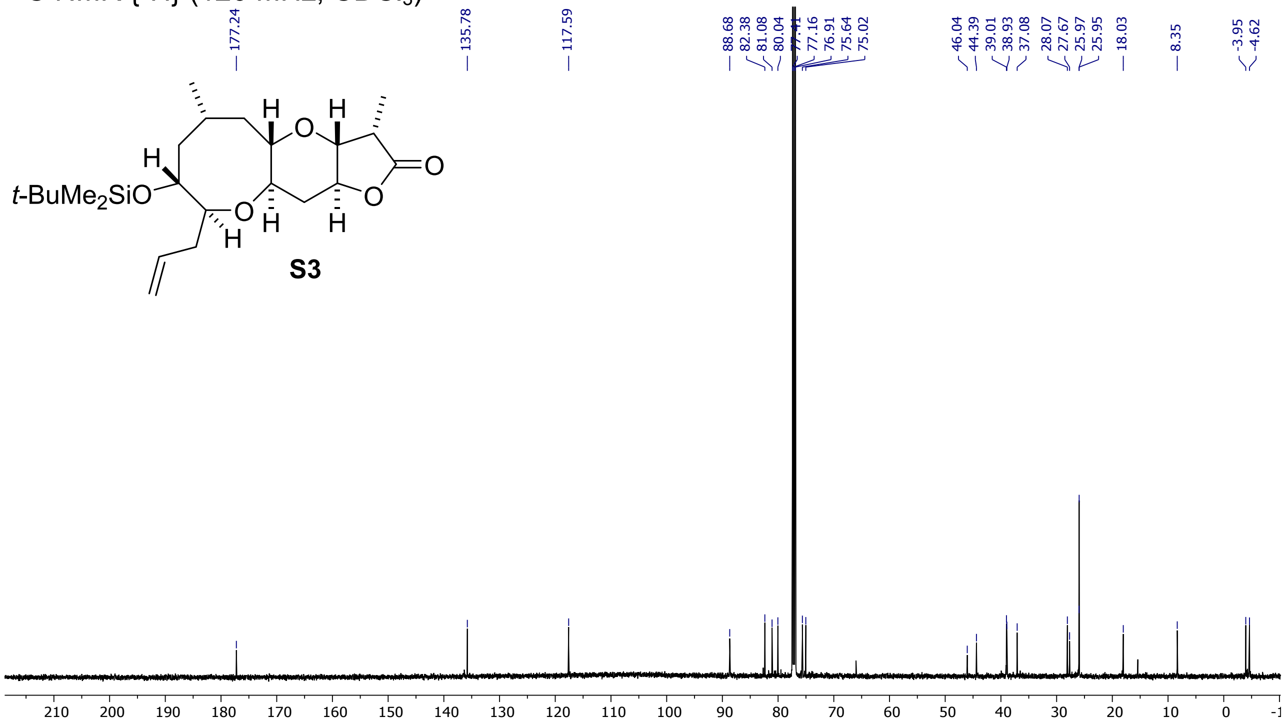

Lactone 15.

^1H NMR (400 MHz, $CDCl_3$)

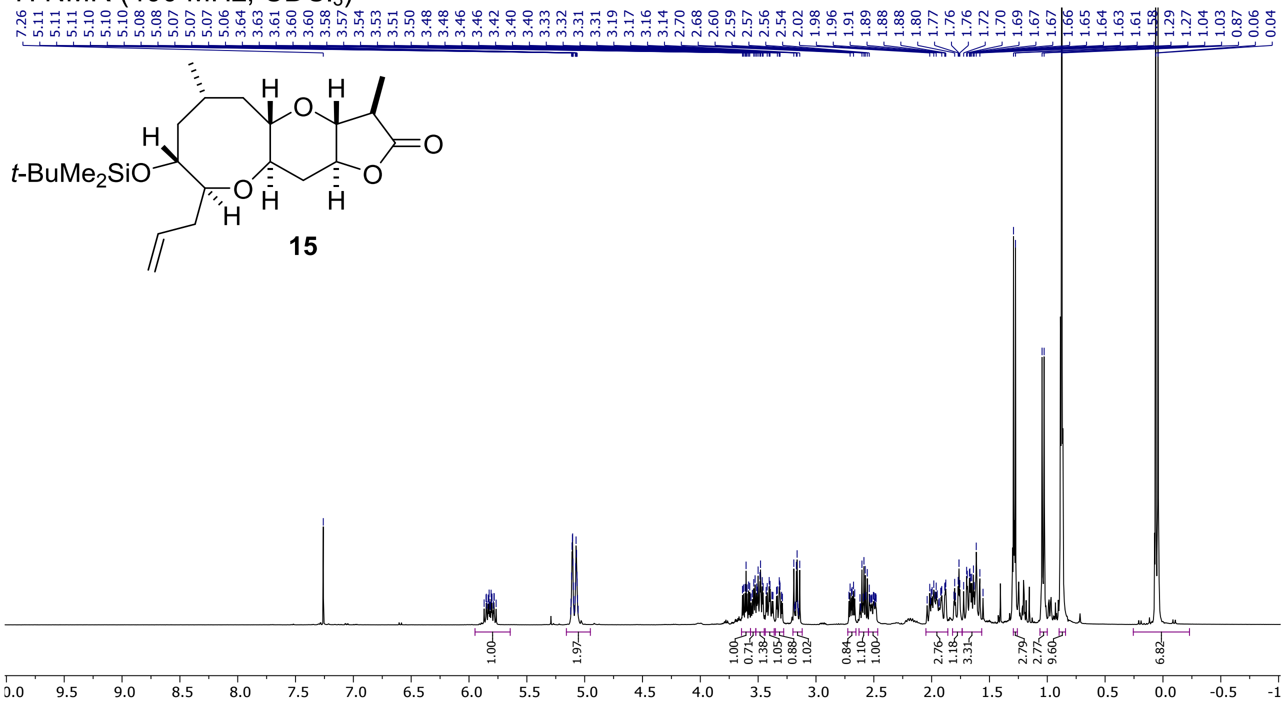

^{13}C NMR {^1H} (101 MHz, $CDCl_3$)

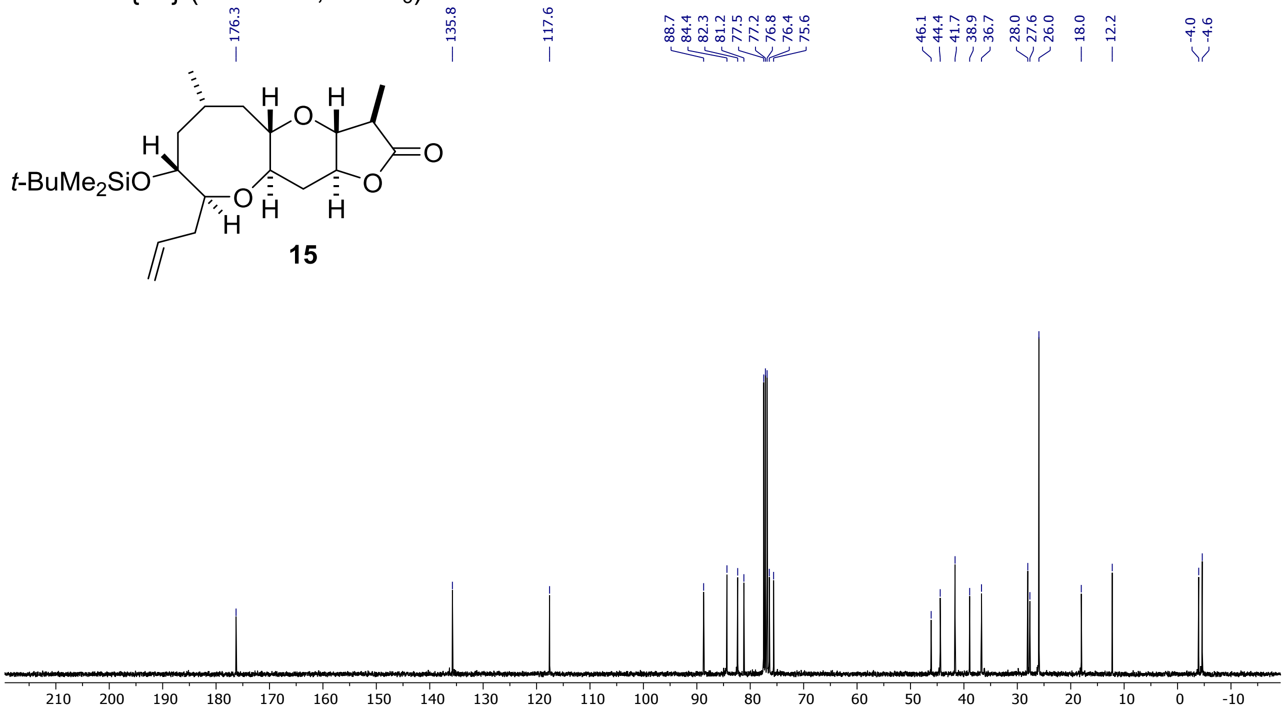

Aldehyde 16.

^{1}H NMR (400 MHz, $CDCl_3$)

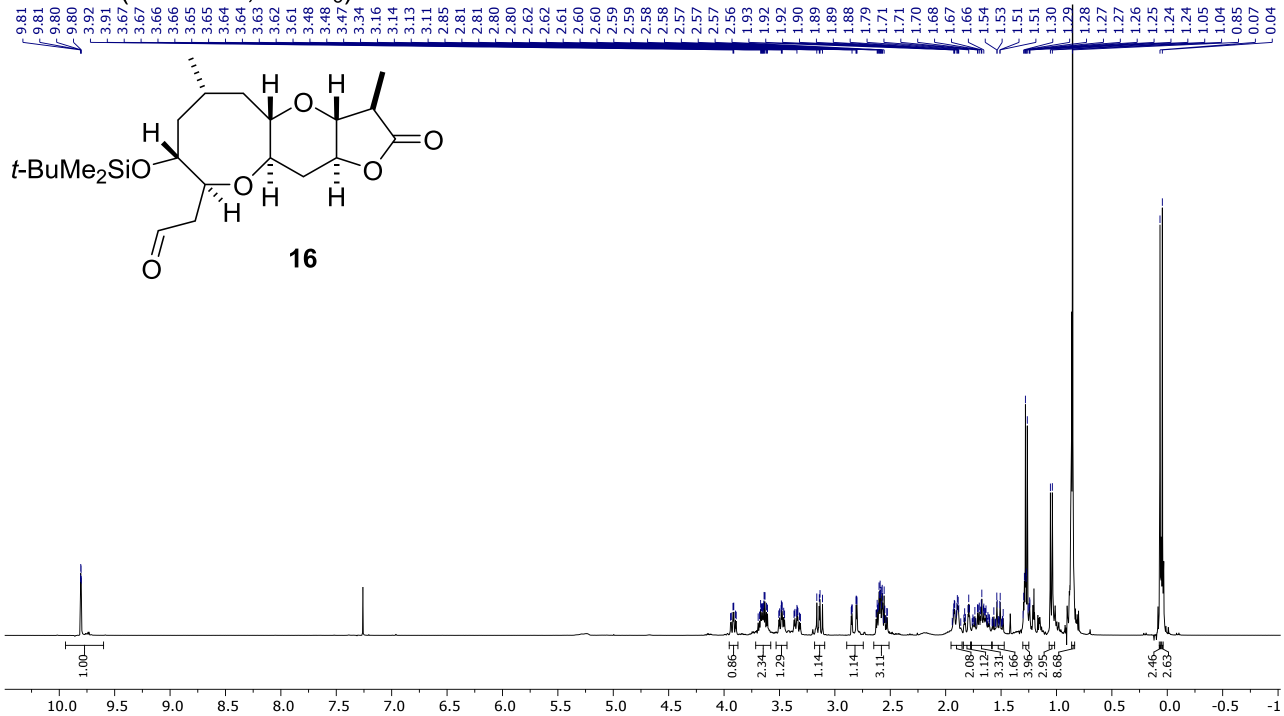

^{13}C NMR {^{1}H} (101 MHz, $CDCl_3$)

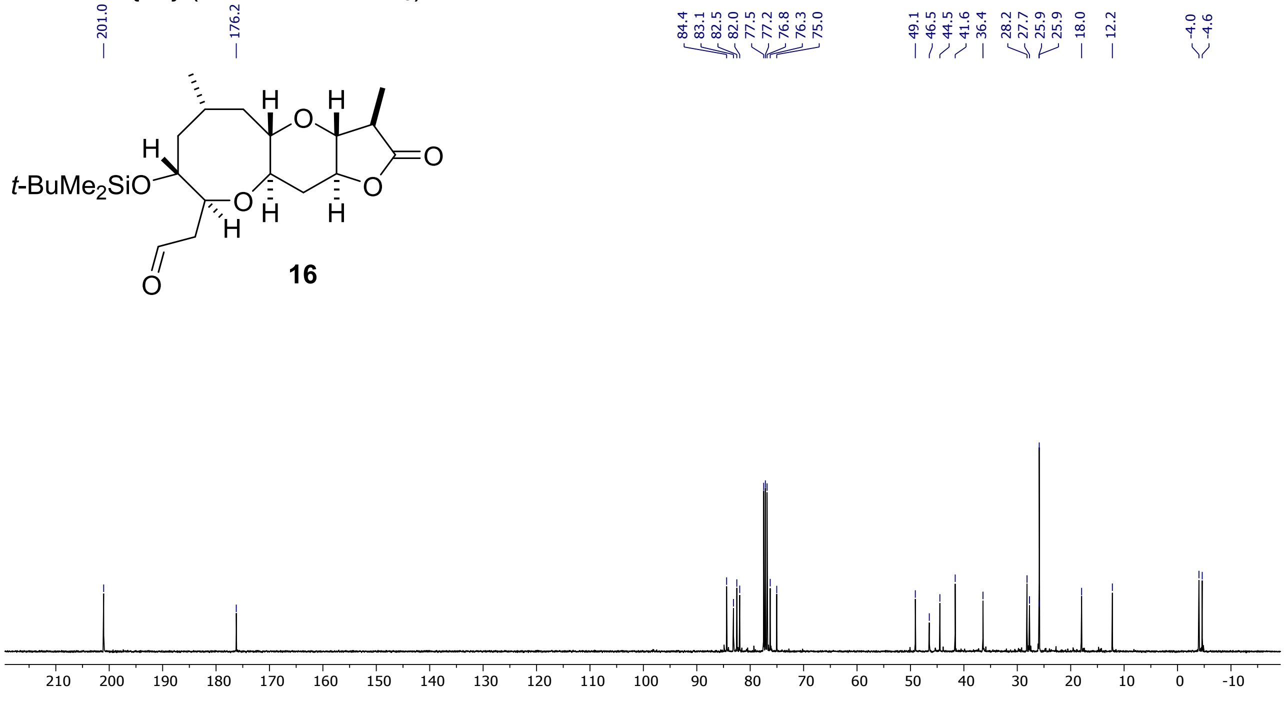

Lactone 17.

^{1}H NMR (400 MHz, $CDCl_3$)

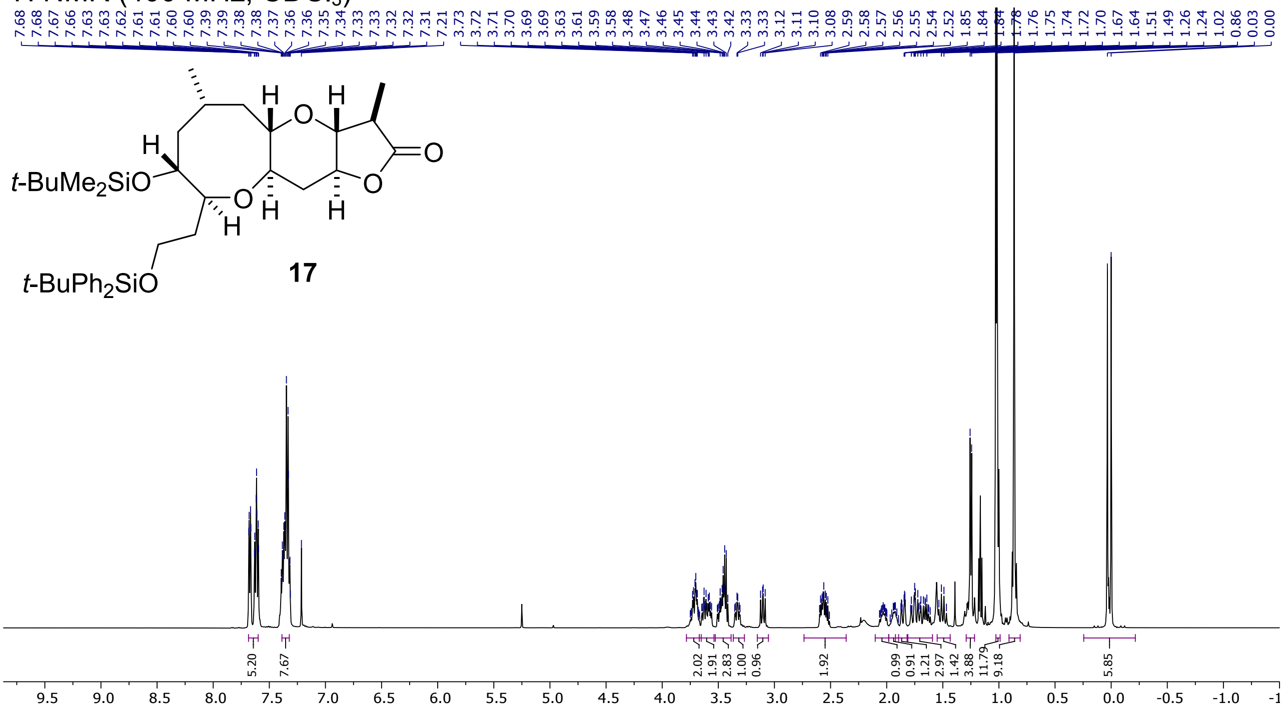

^{13}C NMR {^{1}H} (126 MHz, $CDCl_3$)

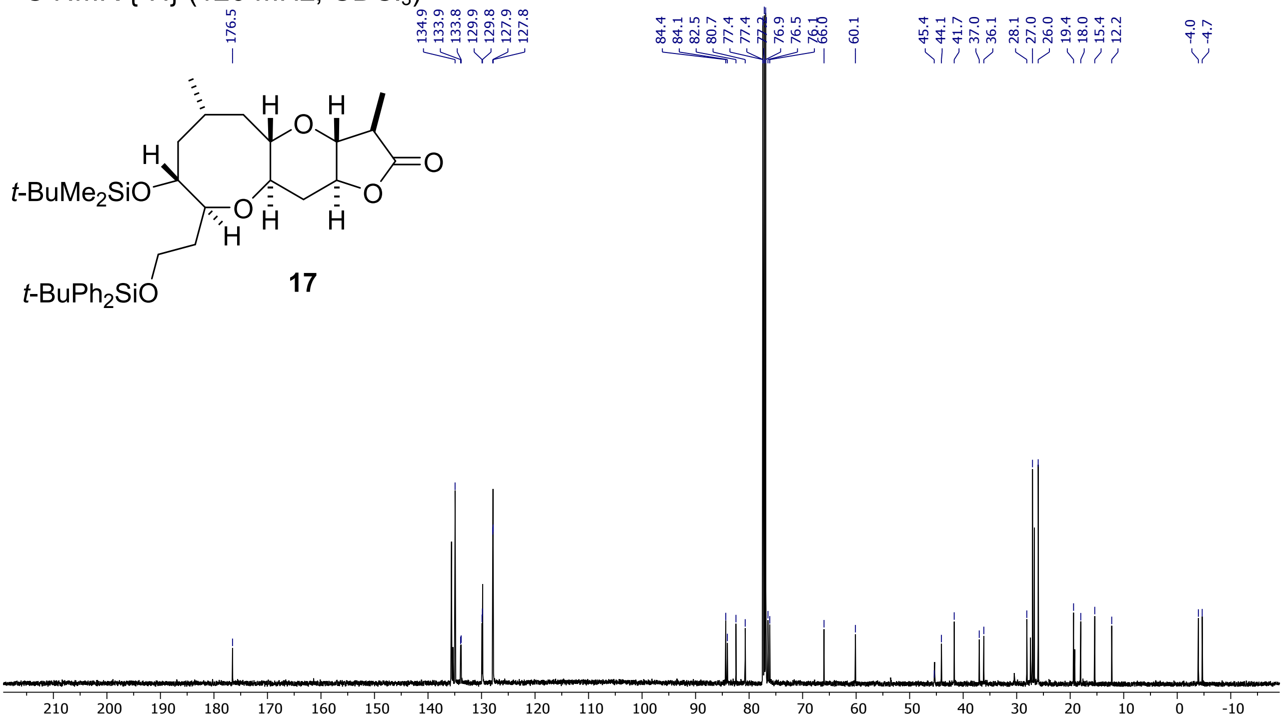

Protected Bicyclic Ether 18.

^{1}H NMR (500 MHz, $CDCl_3$)

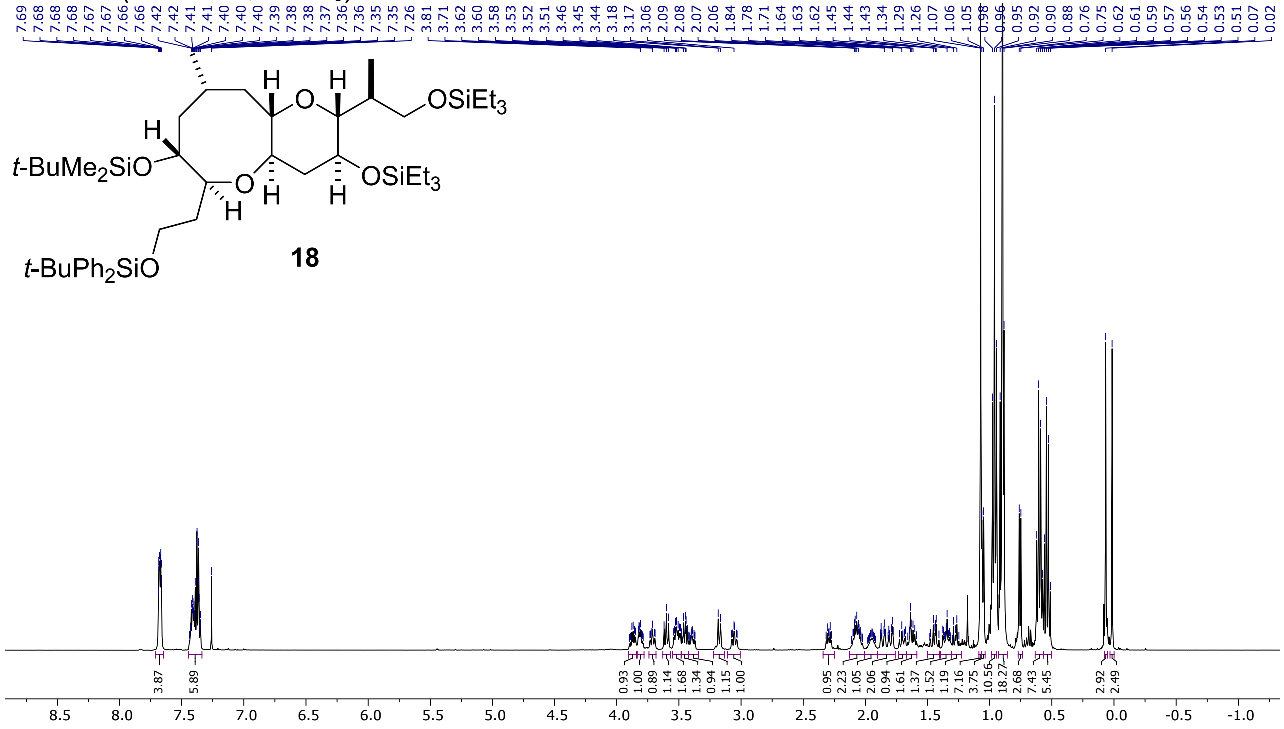

^{13}C NMR {^{1}H} (101 MHz, $CDCl_3$)

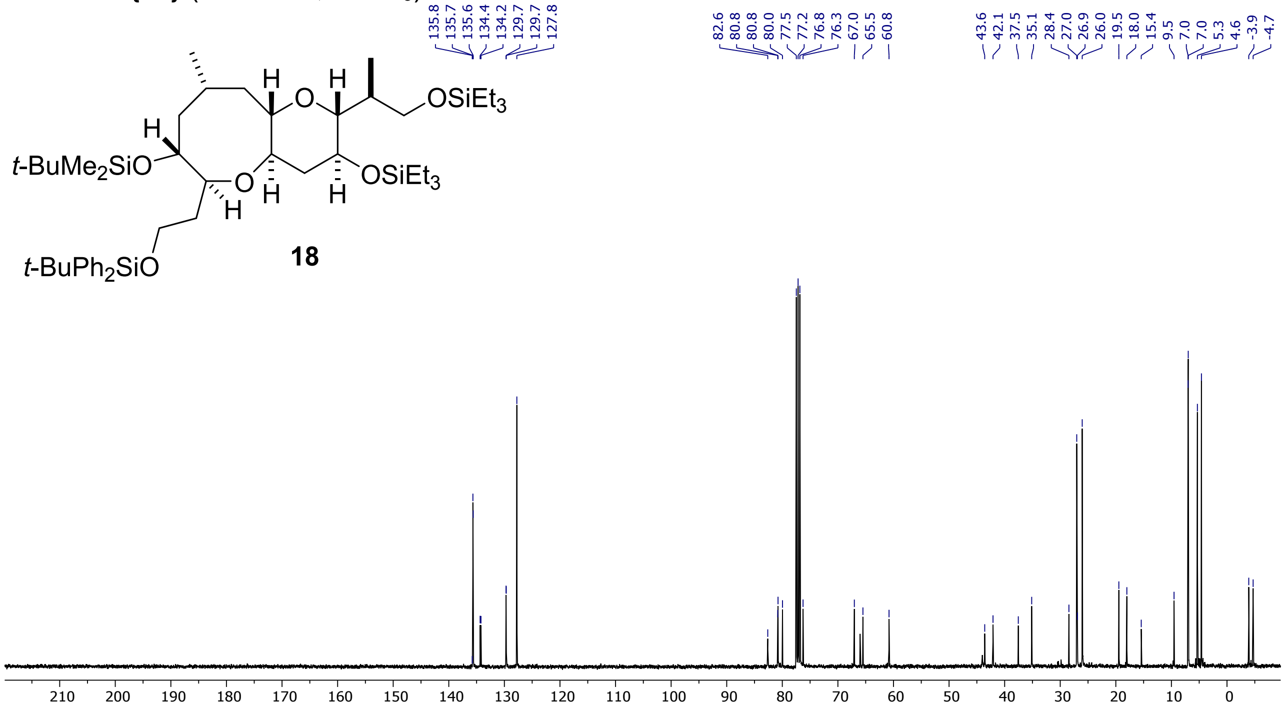

Aldehyde 19.

^{1}H NMR (400 MHz, $CDCl_3$)

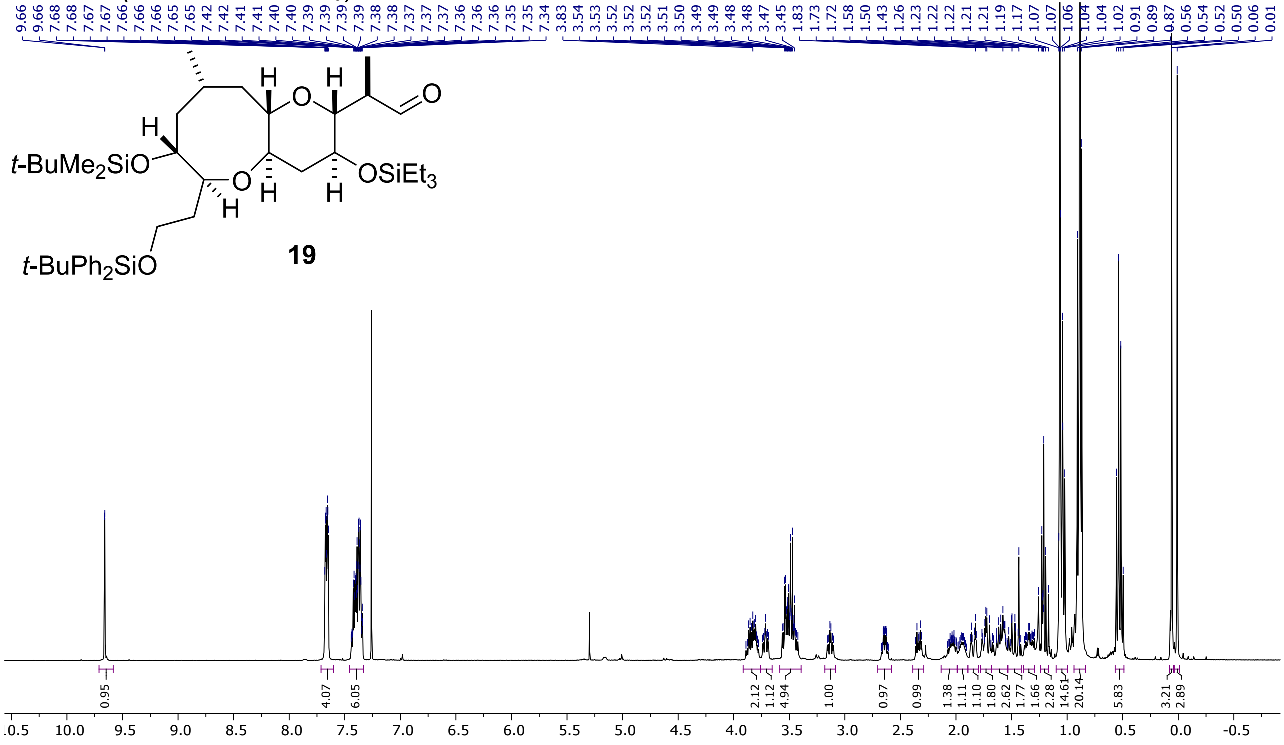

^{13}C NMR {^{1}H} (101 MHz, $CDCl_3$)

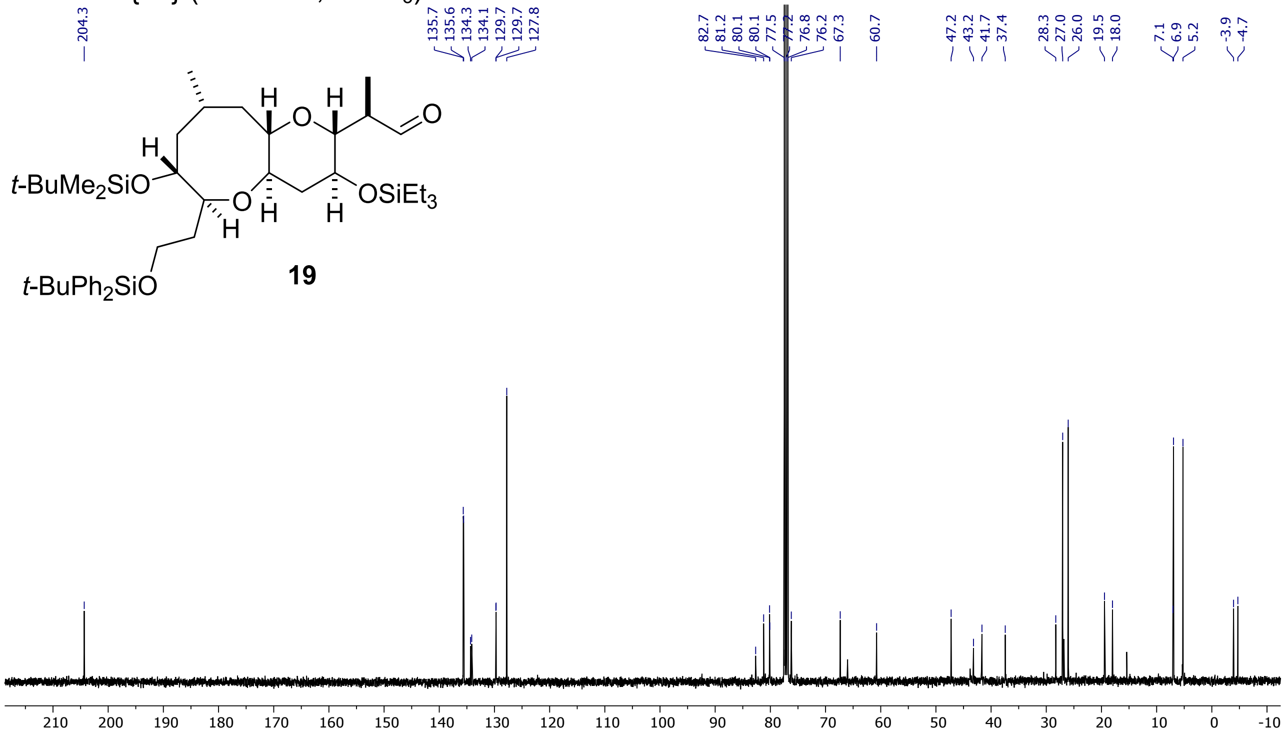

Alcohol 21.

^{1}H NMR (500 MHz, $CDCl_3$)

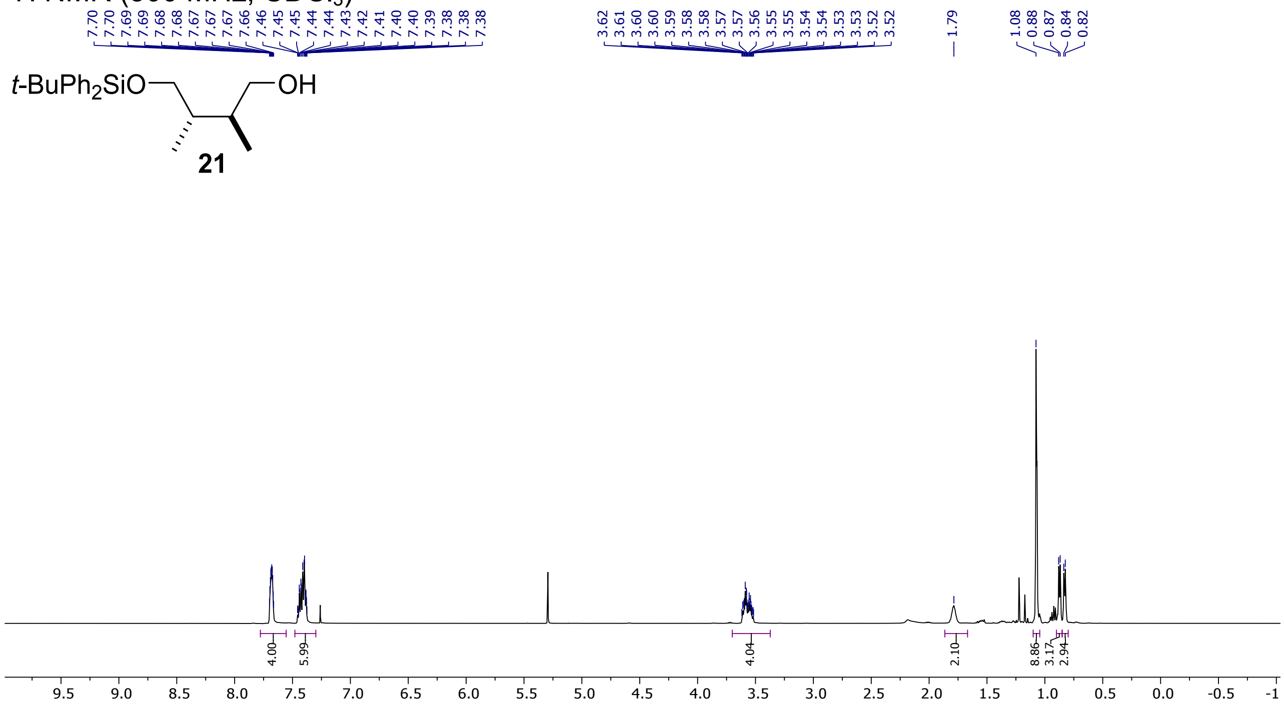

^{13}C NMR {^{1}H} (126 MHz, $CDCl_3$)

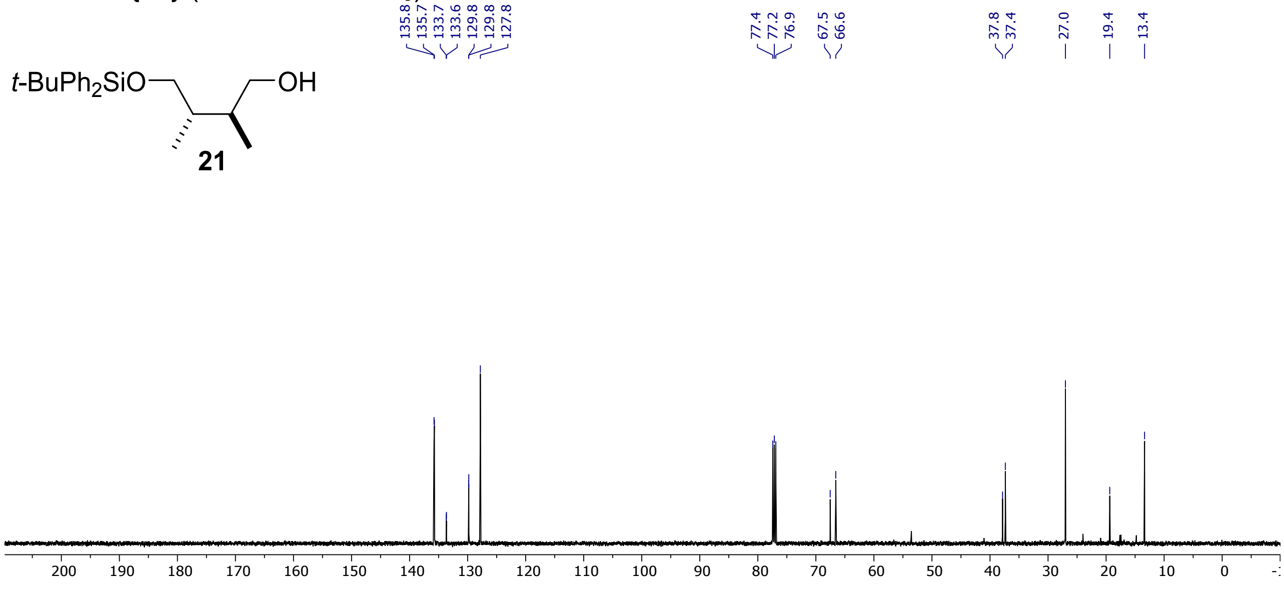

Alkynes 23.

^{1}H NMR (500 MHz, $CDCl_3$)

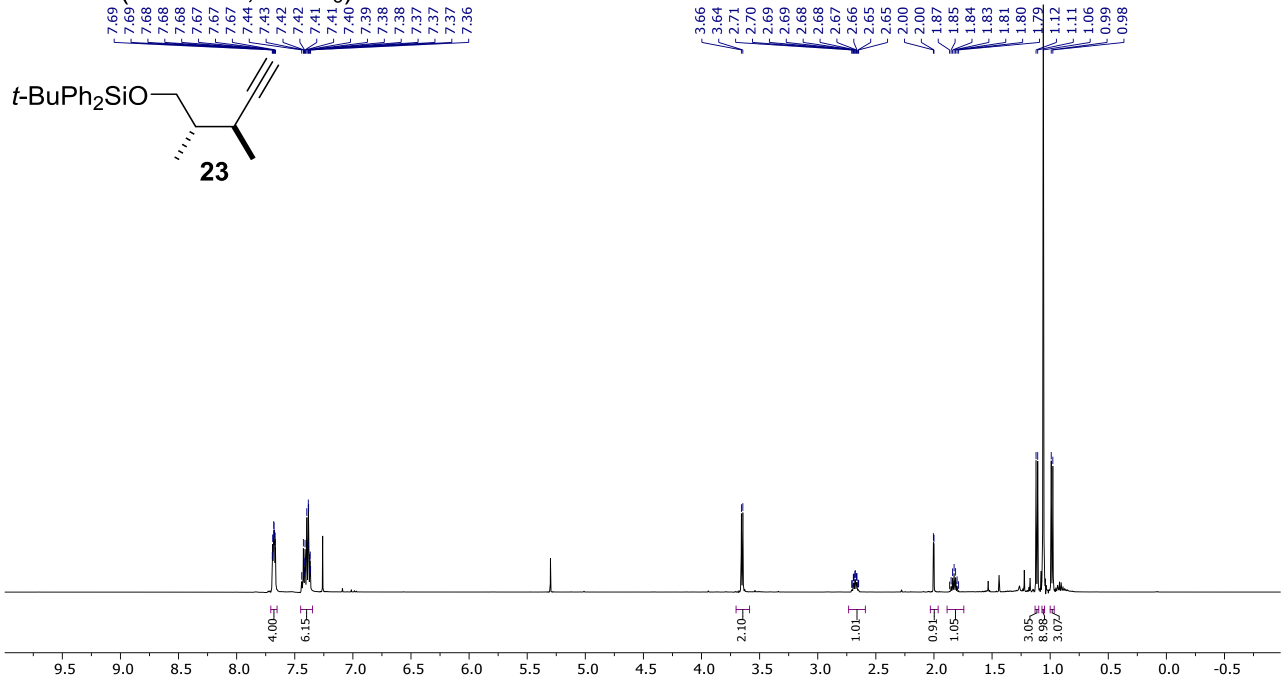

^{13}C NMR {^{1}H} (126 MHz, $CDCl_3$)

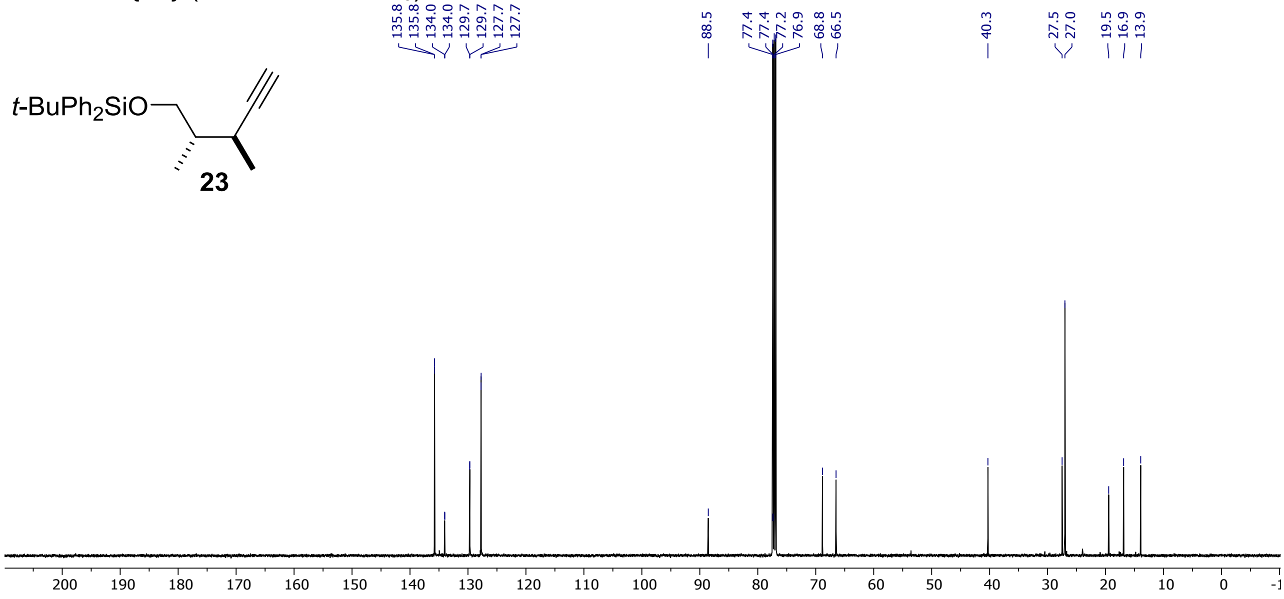

Alkyne 24.

^{1}H NMR (400 MHz, $CDCl_3$)

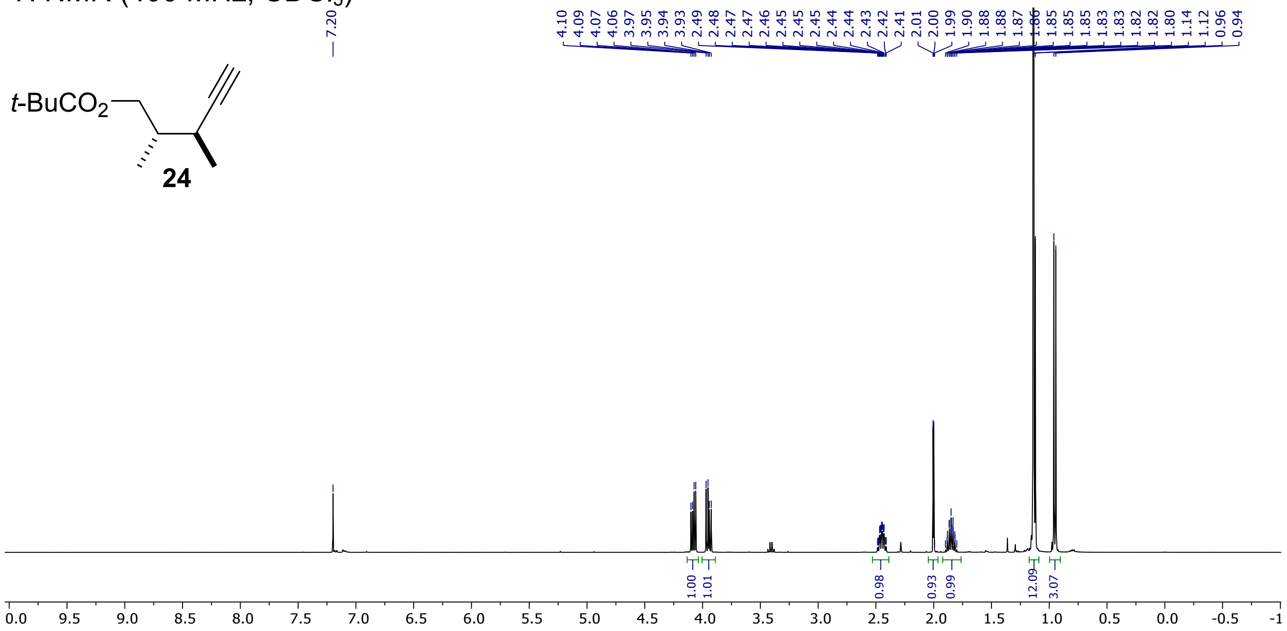

^{13}C NMR {^{1}H} (101 MHz, $CDCl_3$)

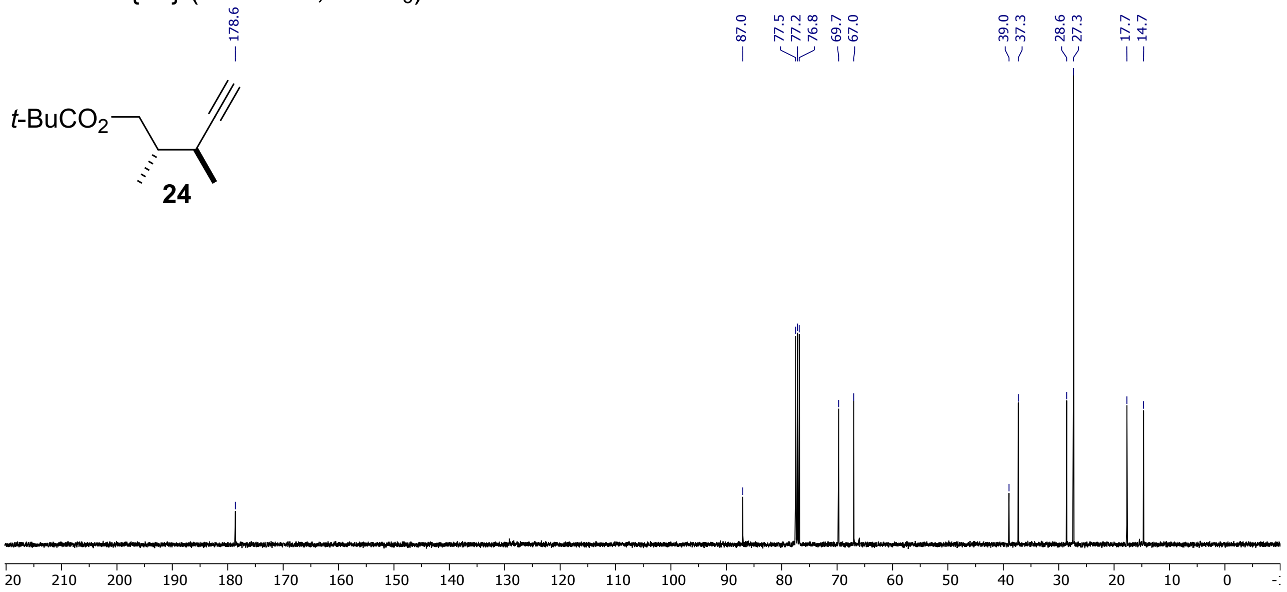

Iodoalkyne 25.

^{1}H NMR (400 MHz, $CDCl_3$)

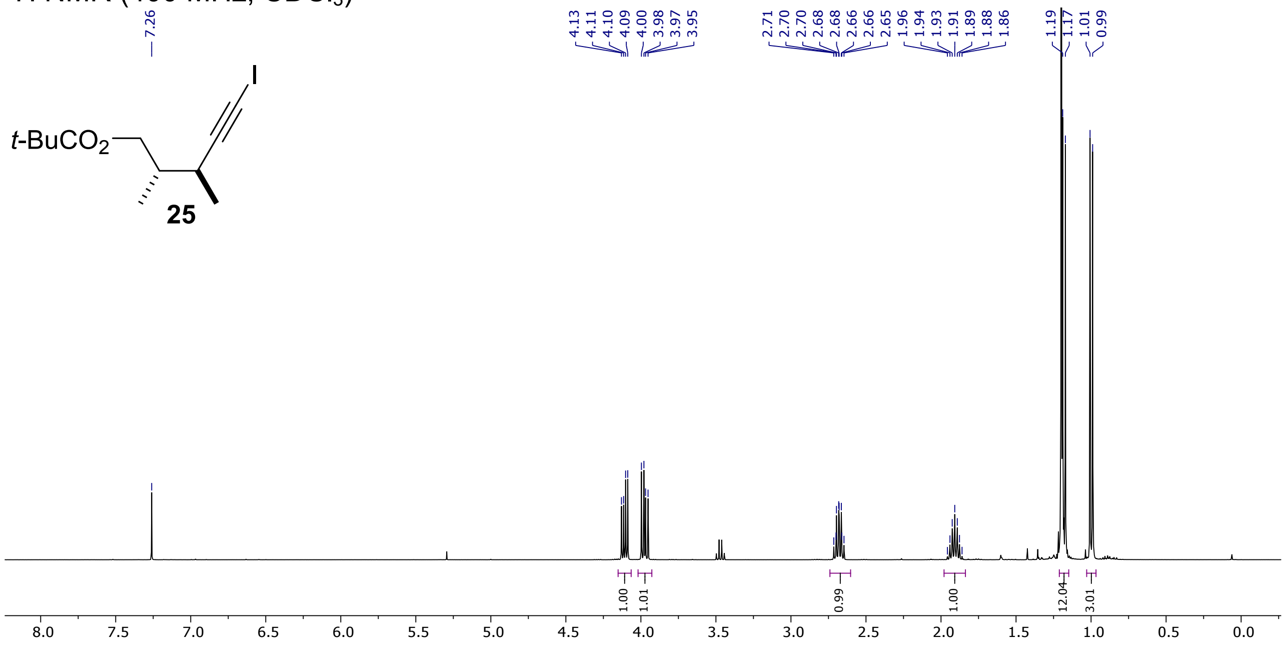

^{13}C NMR {^{1}H} (101 MHz, $CDCl_3$)

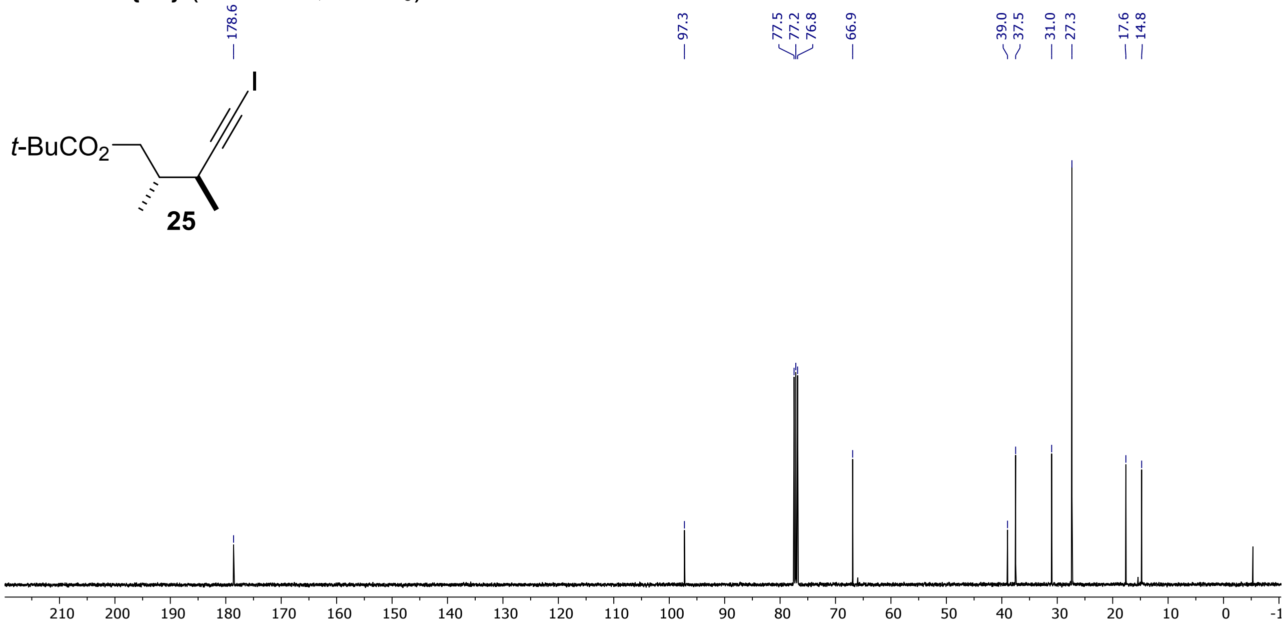

Propargylic Alcohol S4.

^{1}H NMR (500 MHz, $CDCl_3$)

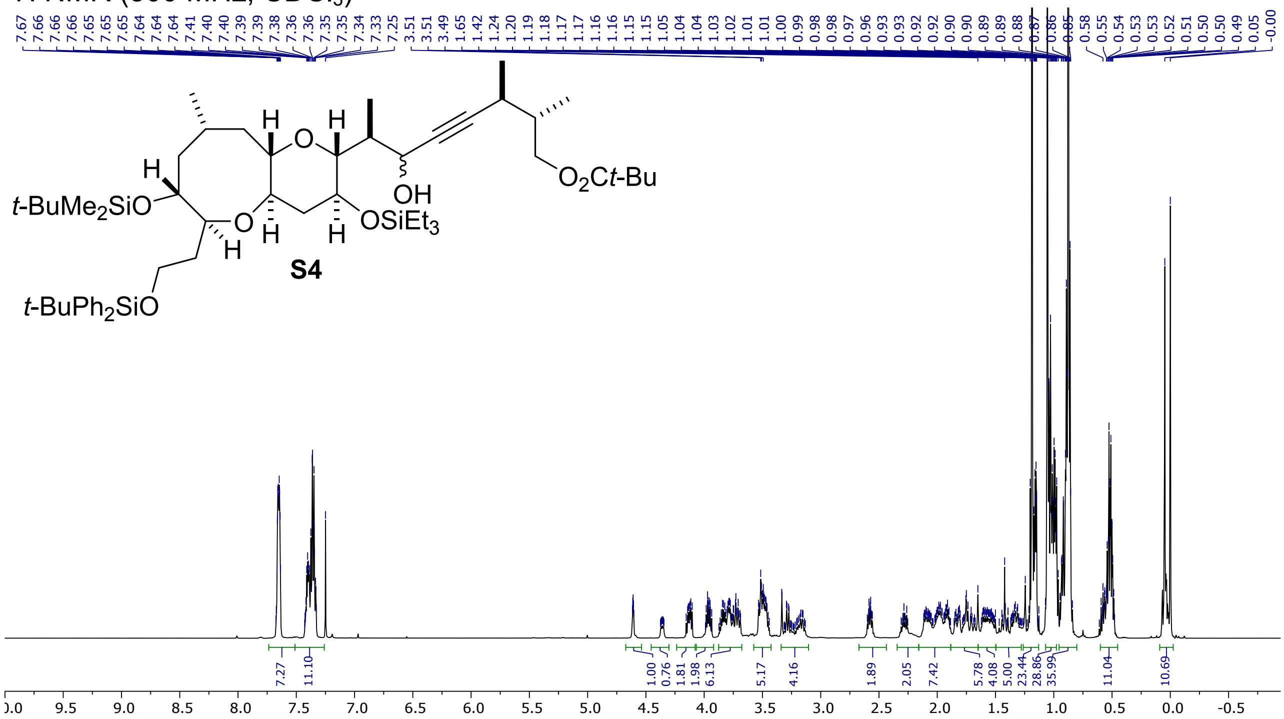

^{13}C NMR {^{1}H} (101 MHz, $CDCl_3$)

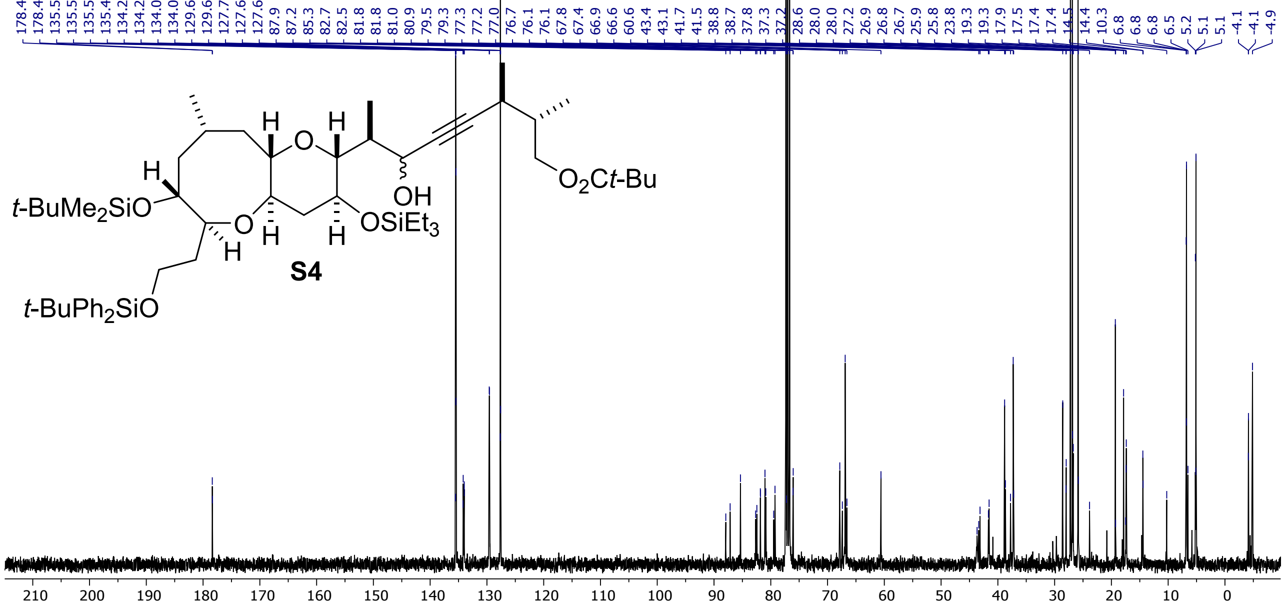

Propargylic Ketone 26.

^{1}H NMR (500 MHz, $CDCl_3$)

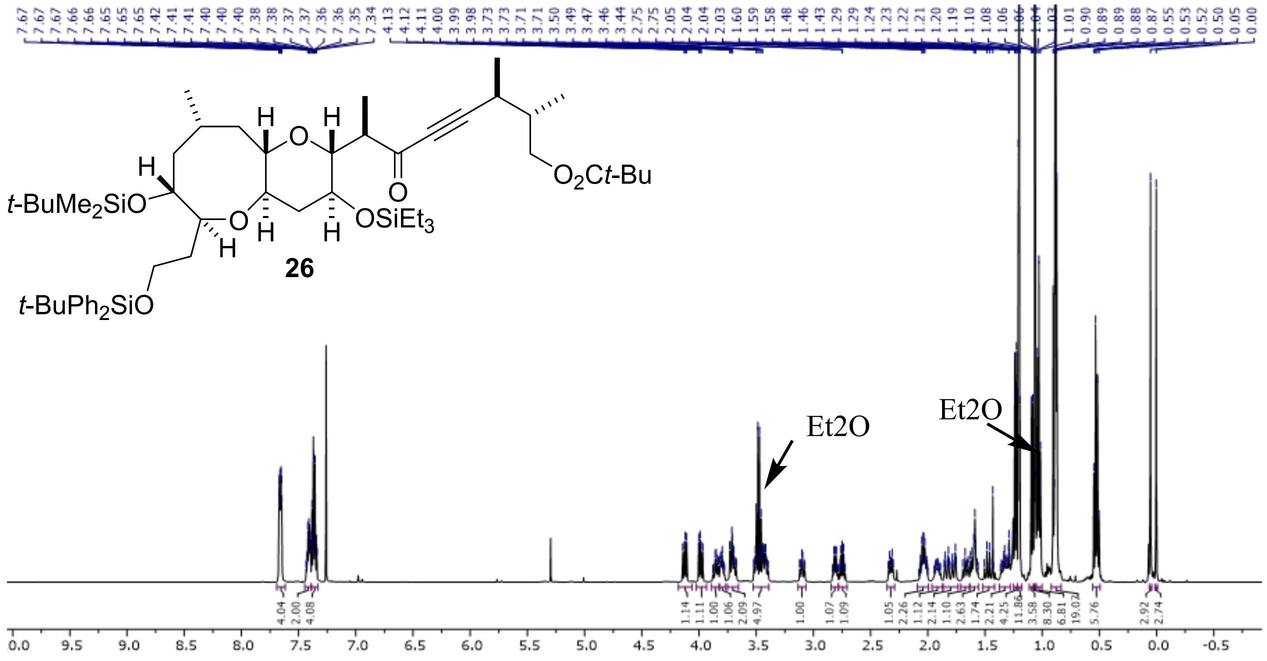

^{13}C NMR {^{1}H} (101 MHz, $CDCl_3$)

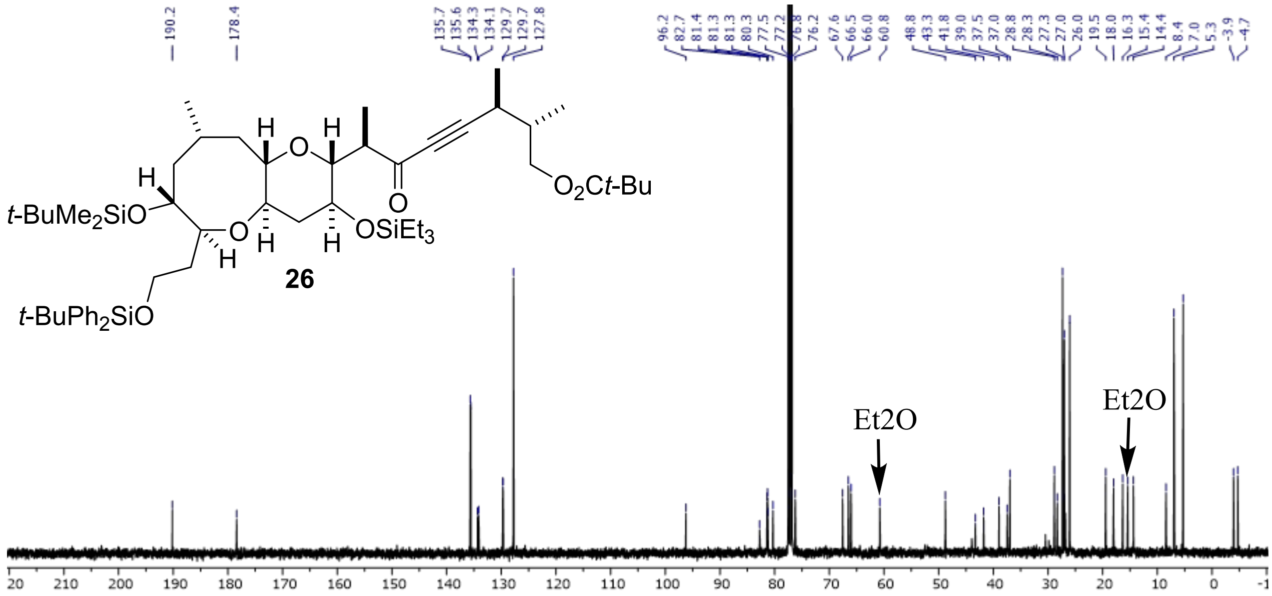

Alcohol S5.

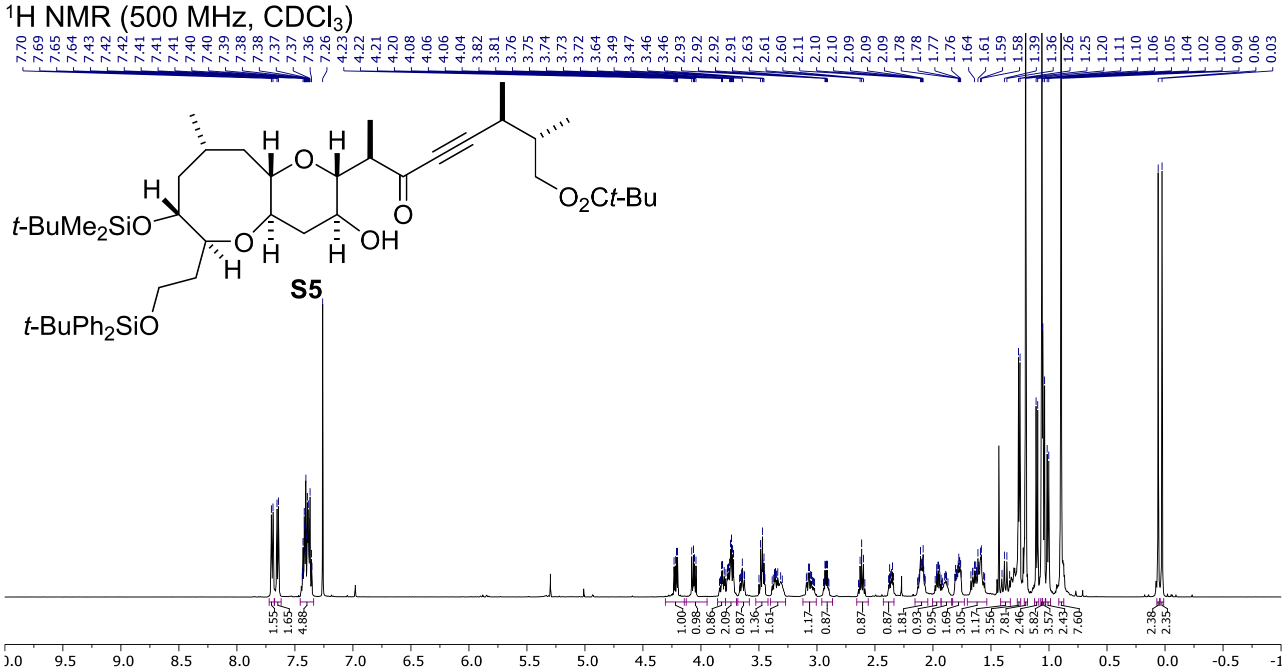

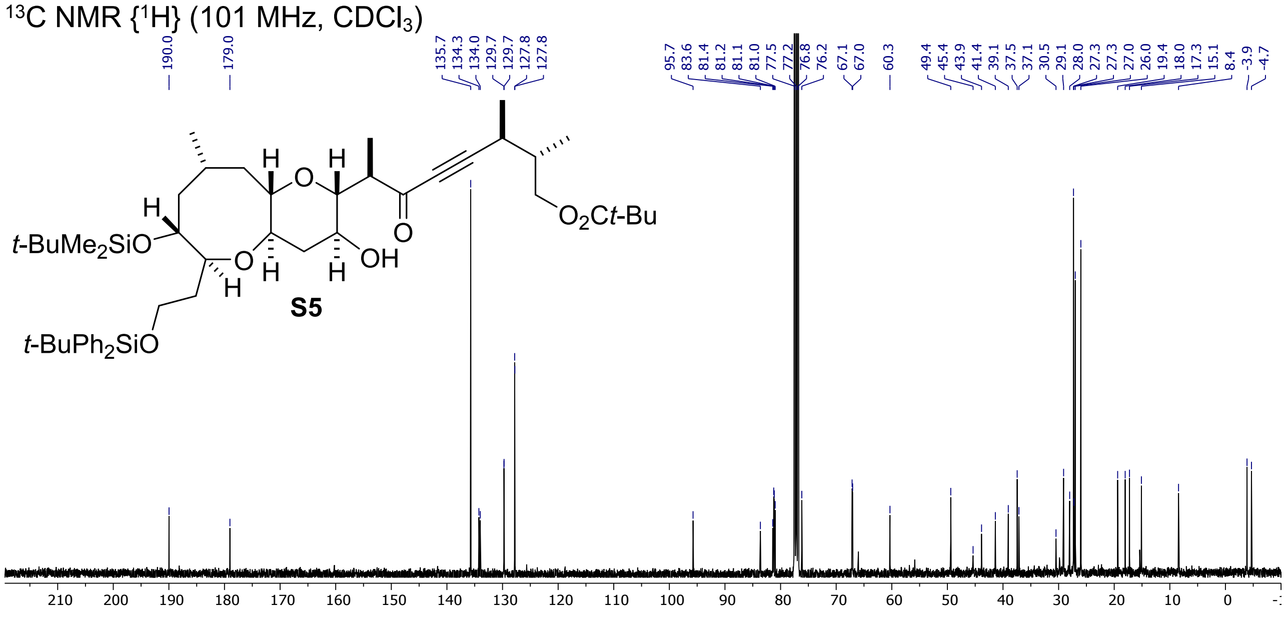

Cyclic Enol Ether 27.

^{1}H NMR (500 MHz, $CDCl_3$)

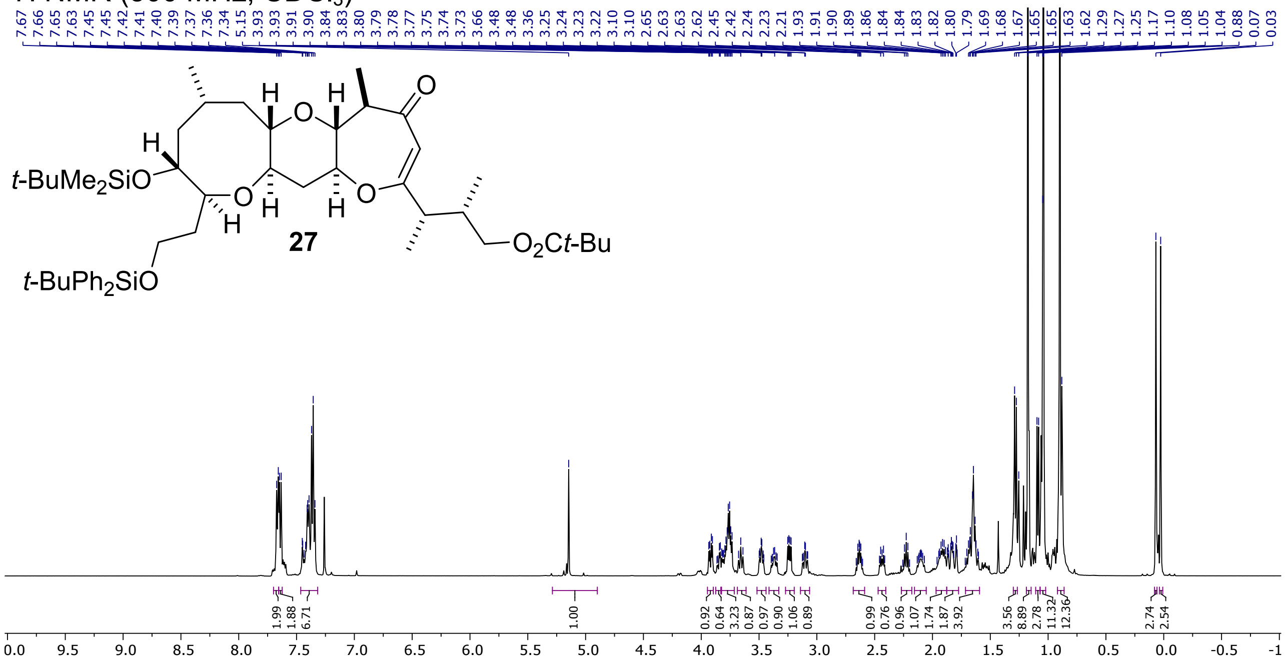

^{13}C NMR {^{1}H} (126 MHz, $CDCl_3$)

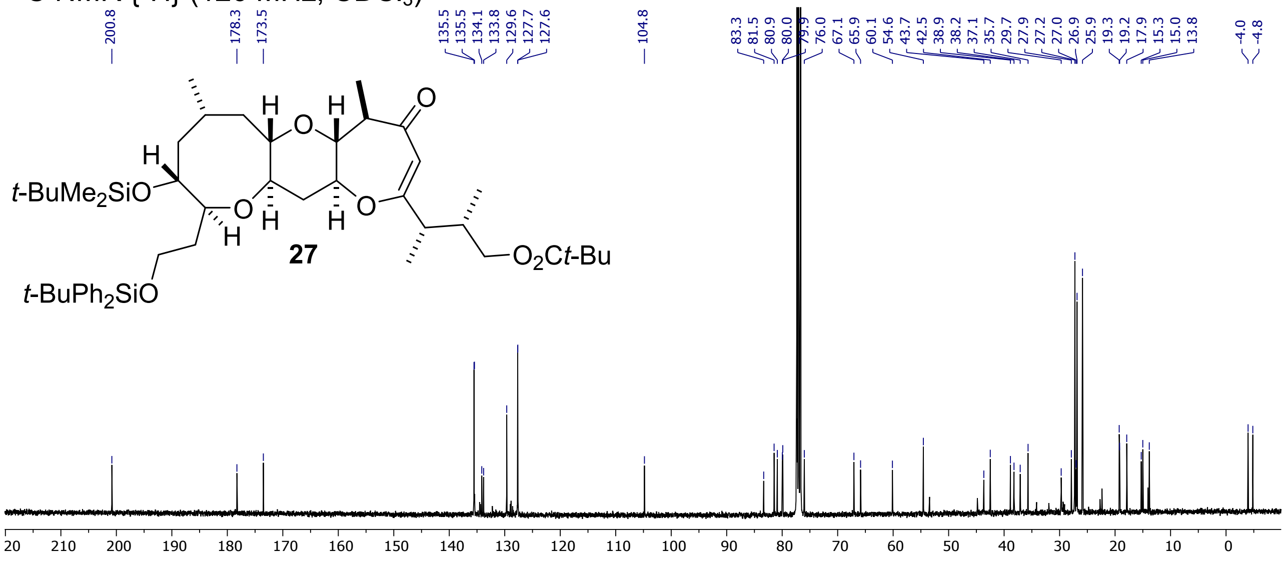

Ketone S6.

^{1}H NMR (500 MHz, $CDCl_3$)

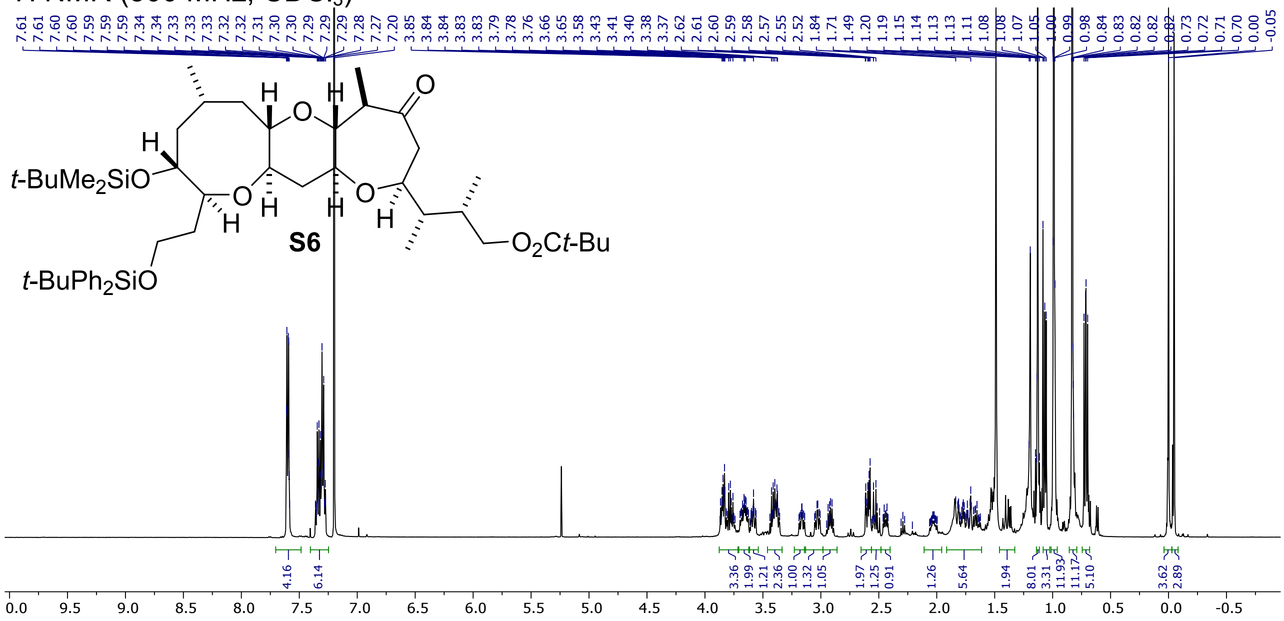

^{13}C NMR {^{1}H} (126 MHz, $CDCl_3$)

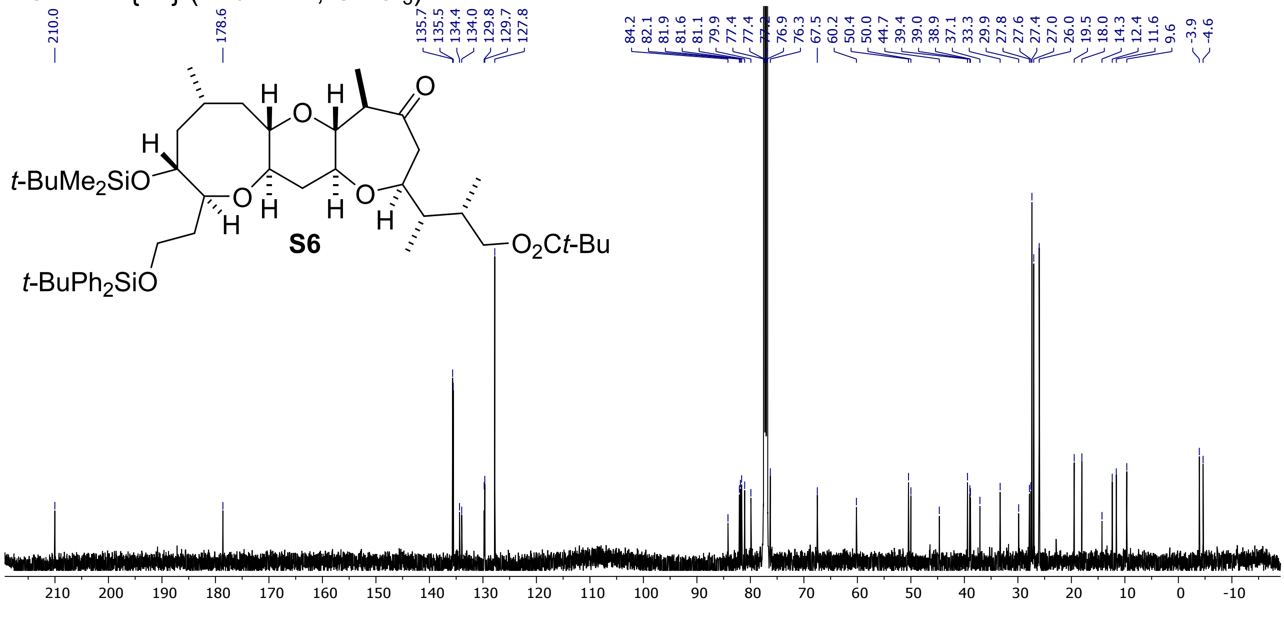

Enol Triflate 28.

^1H NMR (500 MHz, C_6D_6)

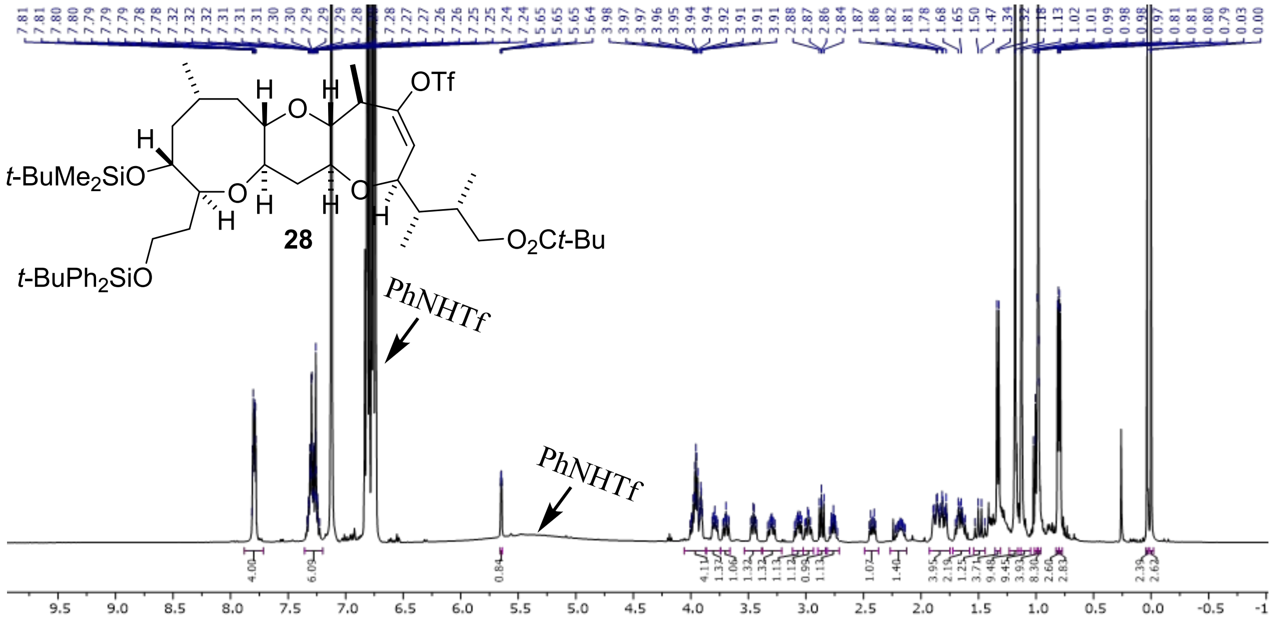

^{13}C NMR {^1H} (126 MHz, C_6D_6)

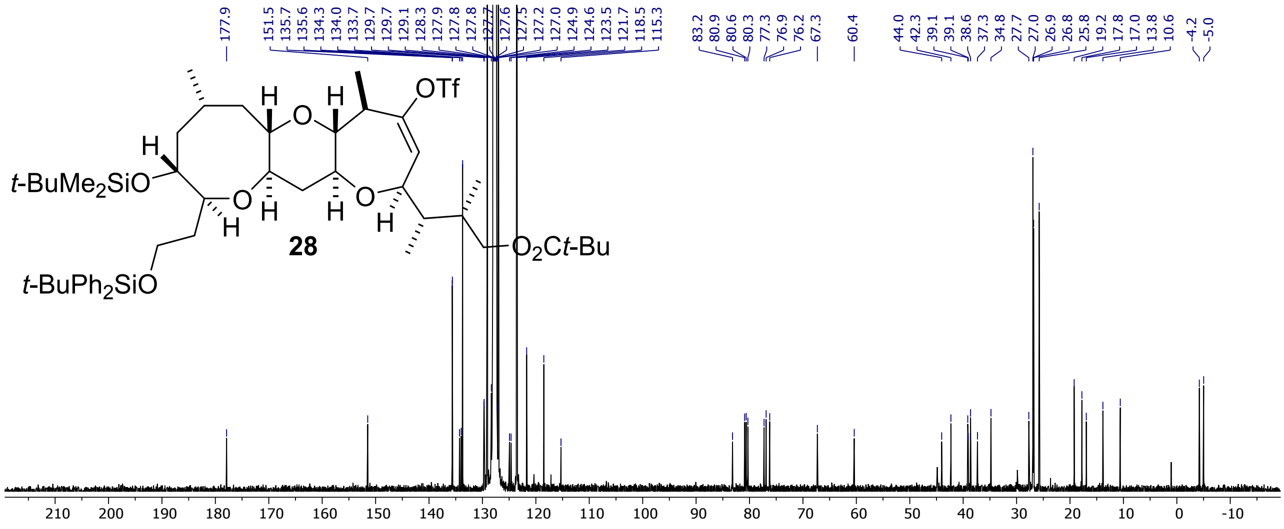

Tricyclic I–K Diol 29.

^{1}H NMR (500 MHz, C_6D_6)

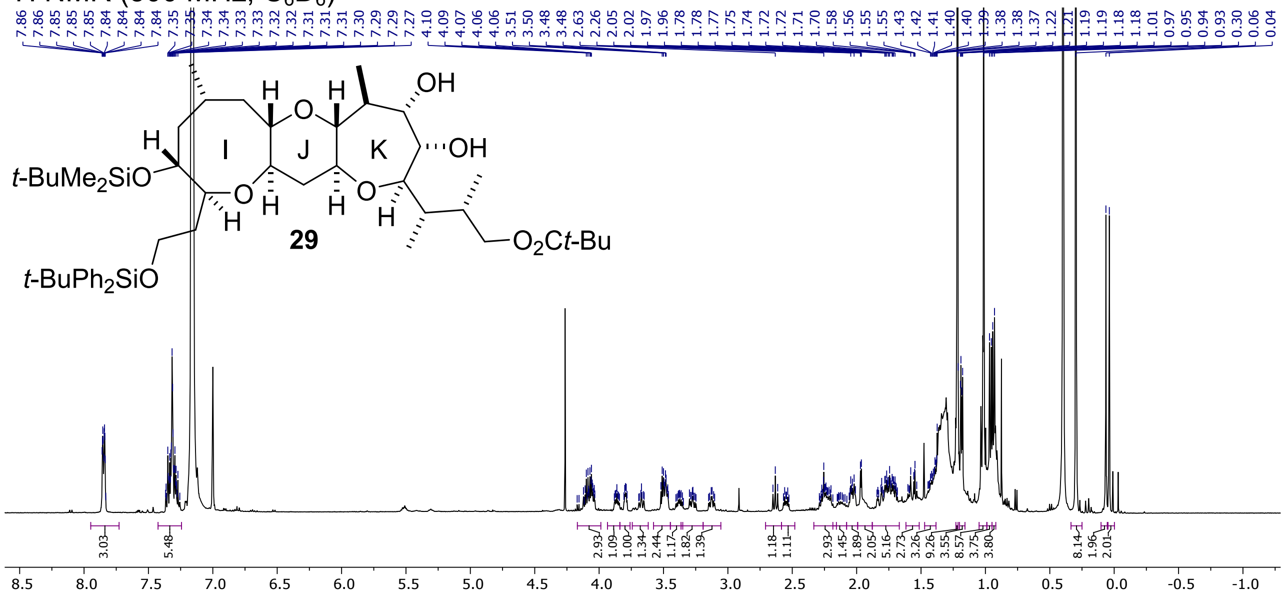

^{13}C NMR {^{1}H} (151 MHz, C_6D_6)

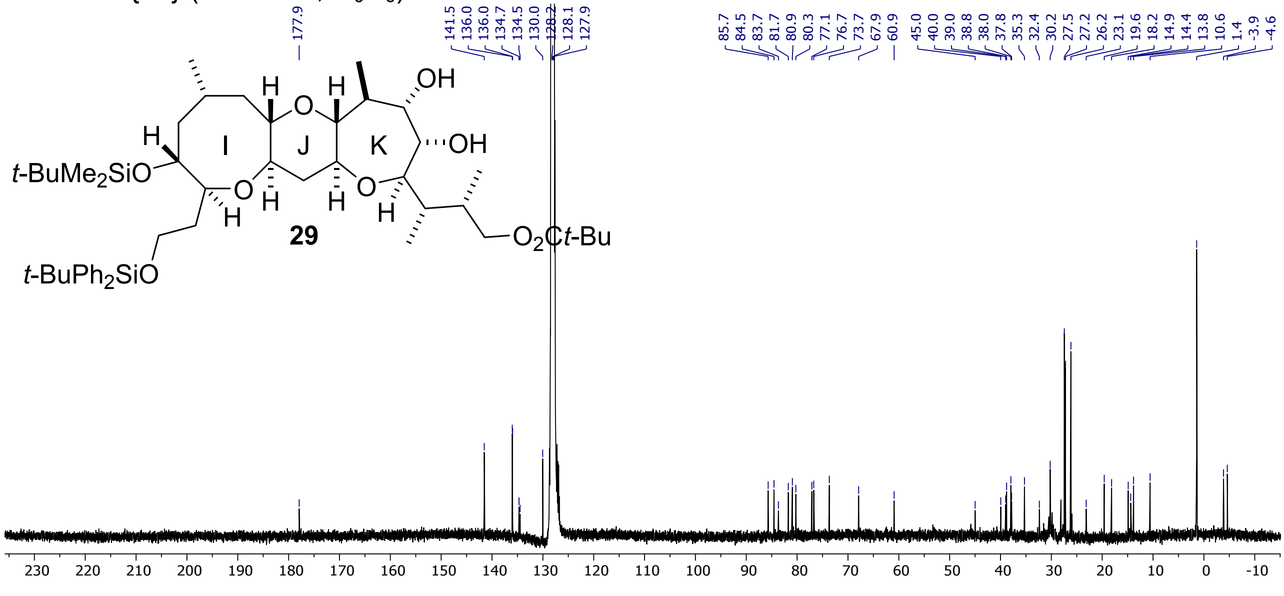

{^{1}H–^{1}H} gNOESY NMR Spectra for Compounds S3, 15, S6, 28 and 29

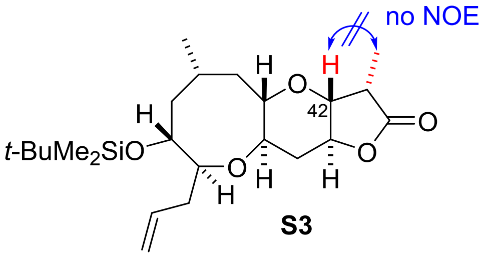

S3

{^{1}H–^{1}H} gNOESY (500 MHz, $CDCl_3$)

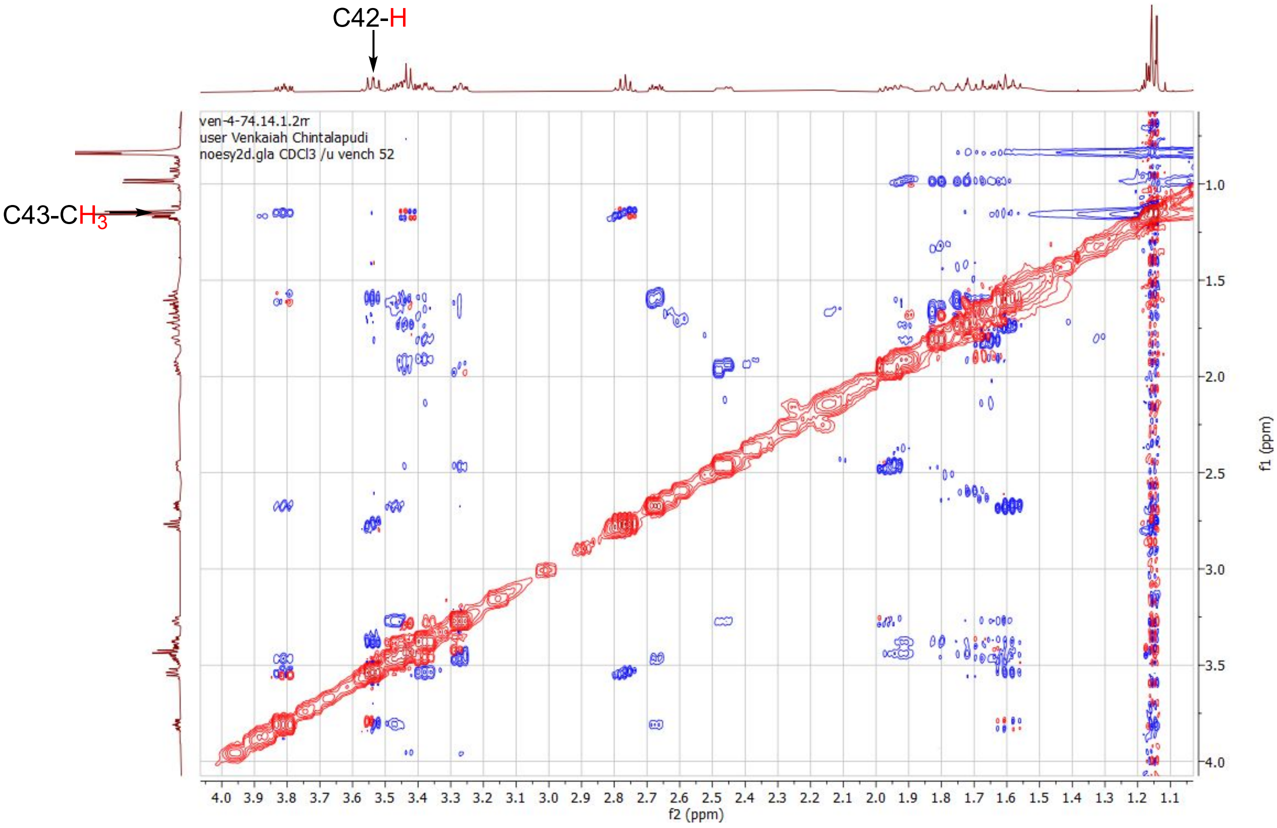

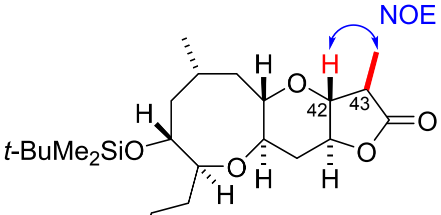

15

{^{1}H–^{1}H} gNOESY (400 MHz, $CDCl_3$)

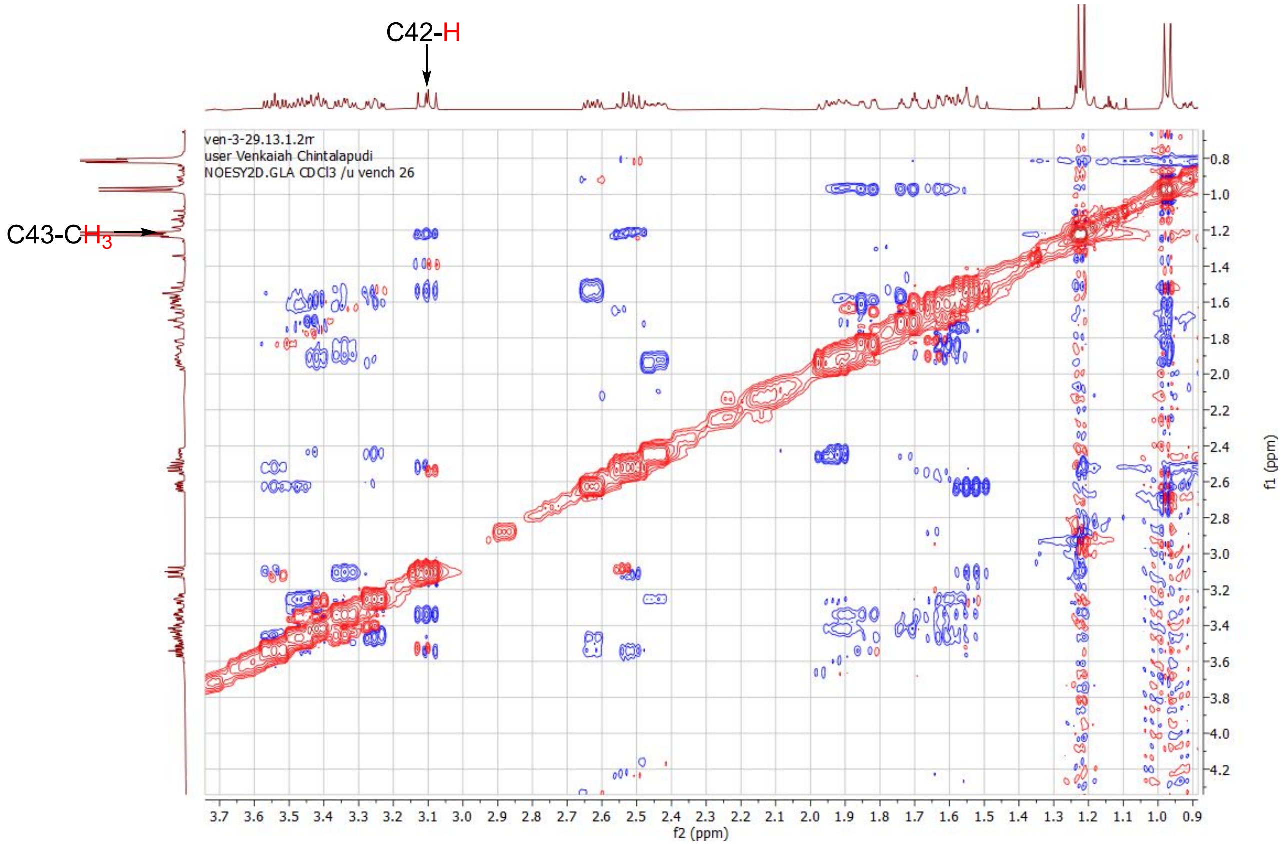

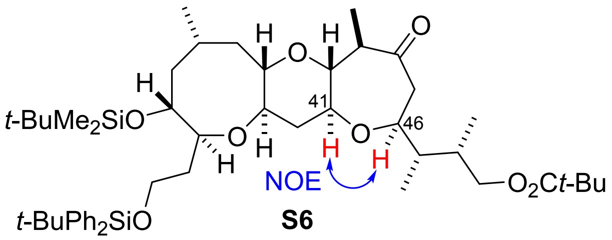

{^{1}H–^{1}H} gNOESY (500 MHz, $CDCl_3$)

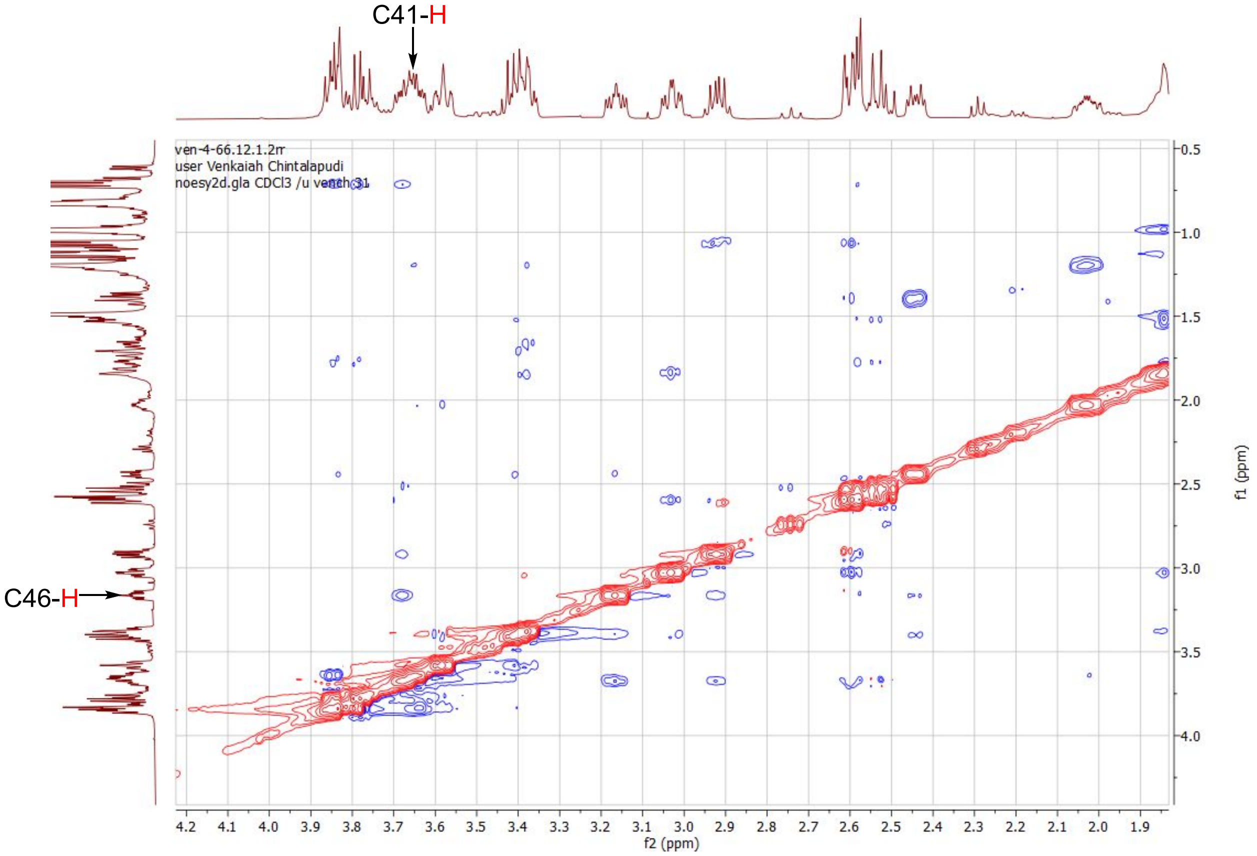

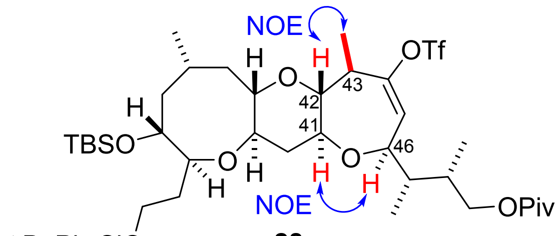

28

{^{1}H–^{1}H} gNOESY (400 MHz, C_6D_6)

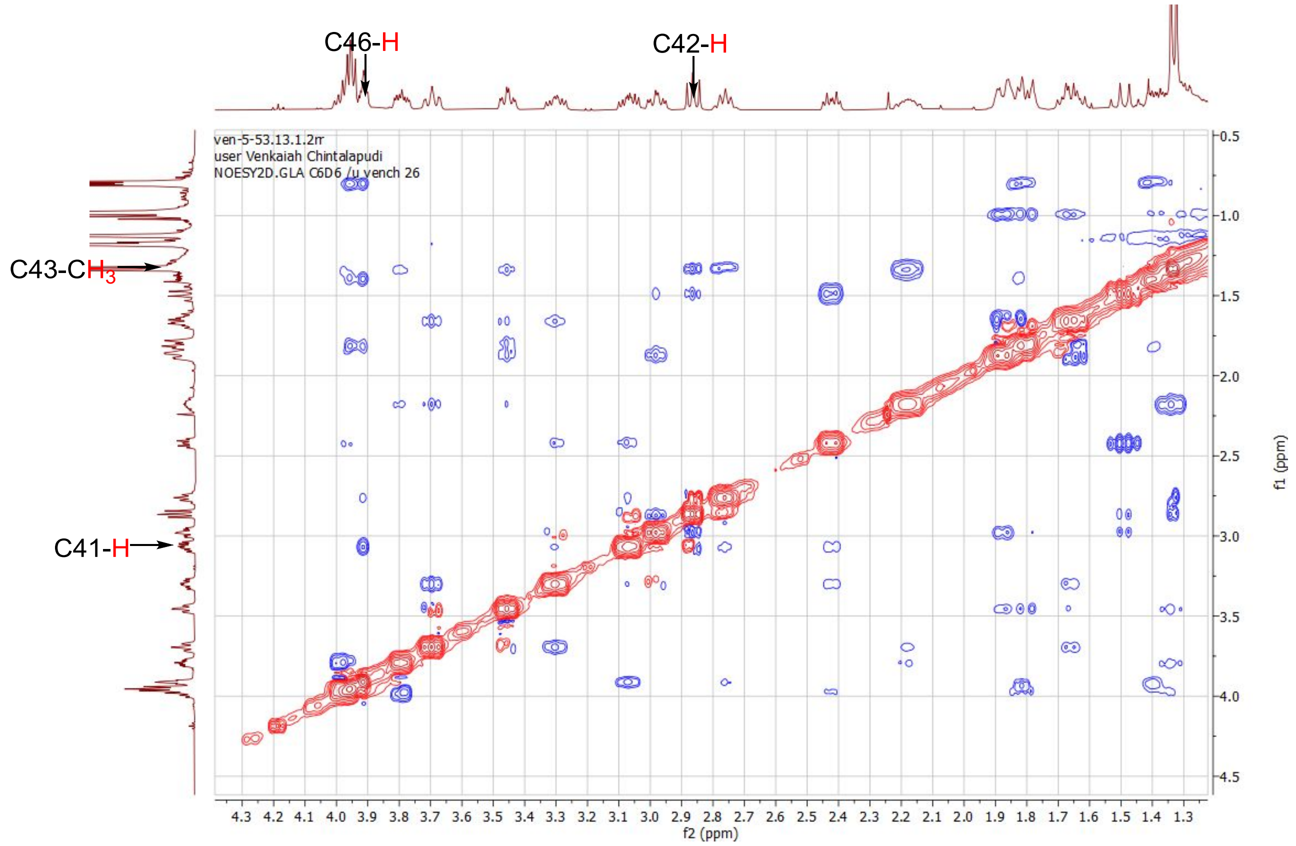

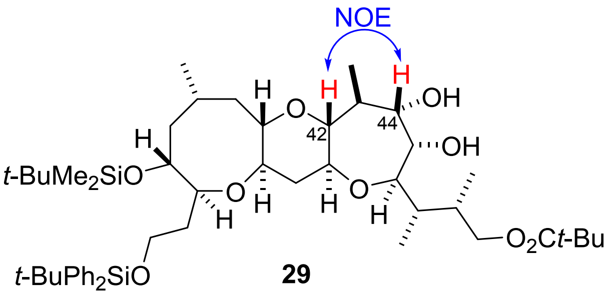

29

{^{1}H–^{1}H} gNOESY (500 MHz, C_6D_6)

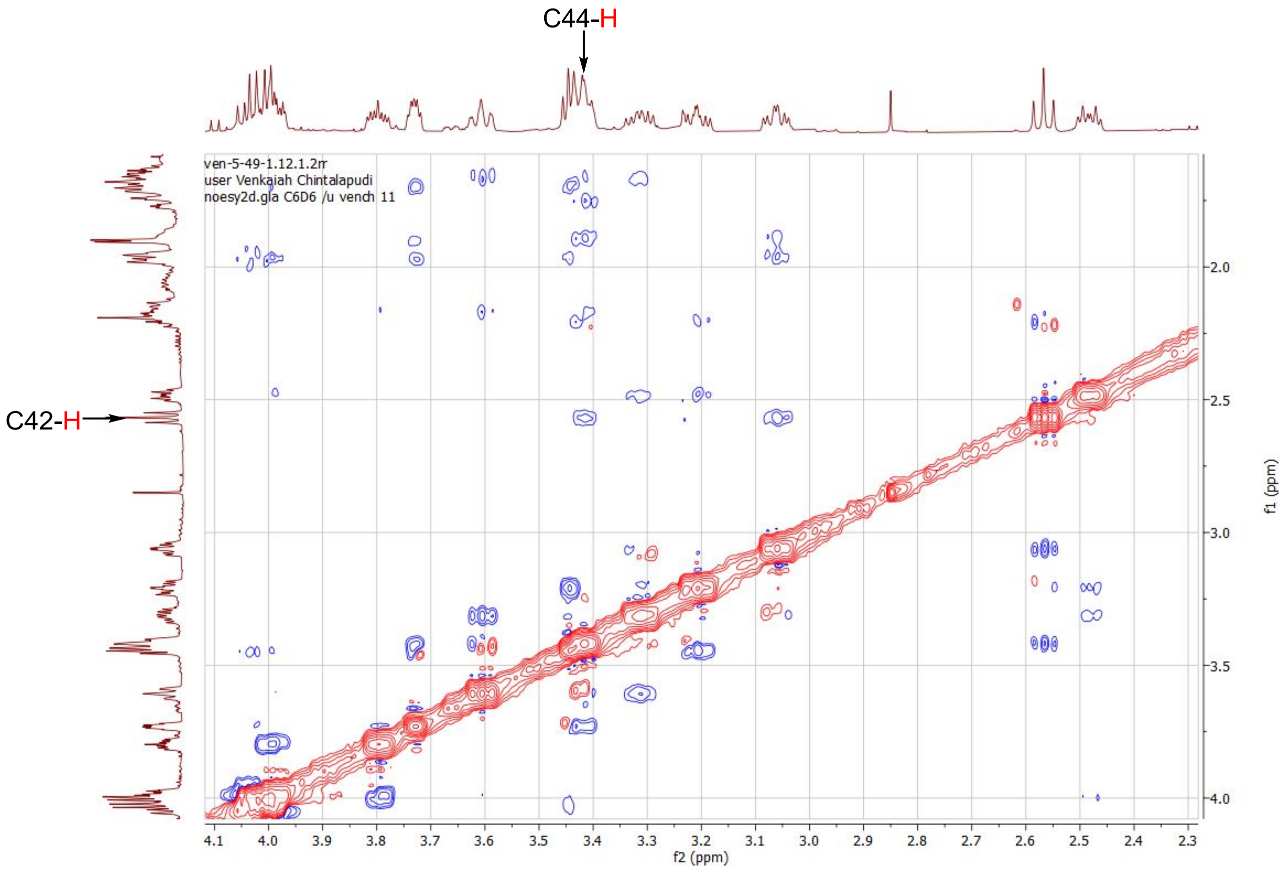

X-Ray Crystal Data, Data Collection and Refinement Information for 10

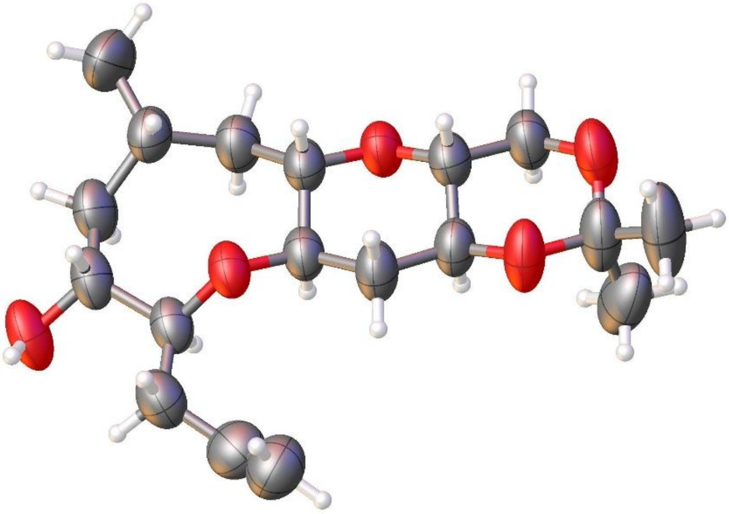

X-ray crystal structure of alcohol **10** (ORTEP plot; ellipsoids at 50% probability)

Crystal data

$C_{18}H_{30}O_5$	F(000) = 1424
M_r = 326.42	D_x = 1.150 Mg m^{-3}
Monoclinic, C2	Cu$K\alpha$ radiation, λ = 1.54178 Å
a = 25.8582 (9) Å	Cell parameters from 9899 reflections
b = 5.2537 (2) Å	θ = 3.2–74.4°
c = 28.3908 (10) Å	μ = 0.67 mm^{-1}
β = 102.253 (2)°	T = 273 K
V = 3769.1 (2) Å^3	Lath, colourless
Z = 8	0.36 × 0.12 × 0.02 mm

Data collection

Bruker D8 VENTURE diffractometer	7506 independent reflections
Radiation source: microfocus sealed tube, INCOATEC Iμs 3.0	6454 reflections with $I > 2\sigma(I)$
Multilayer mirror optics monochromator	R_{int} = 0.048
Detector resolution: 7.4074 pixels mm^{-1}	θ_{max} = 74.7°, θ_{min} = 3.2°
Φ and ω scans	h = −31→32
Absorption correction: Multi-scan *SADABS2016*/2 (Bruker,2016/2) was used for absorption correction. wR2(int) was 0.1393 before and 0.0754 after correction. The ratio of minimum to maximum transmission is 0.7697. The l/2 correction factor is not present.	k = −6→6
T_{min} = 0.580, T_{max} = 0.754	l = −35→35
23966 measured reflections	

Refinement

Refinement on F^2	Hydrogen site location: mixed
Least-squares matrix: full	H atoms treated by a mixture of independent and constrained refinement
$R[F^2 > 2\sigma(F^2)] = 0.051$	$w = 1/[\sigma^2(F_o^2) + (0.0978P)^2 + 0.1657P]$ where $P = (F_o^2 + 2F_c^2)/3$
$wR(F^2) = 0.158$	$(\Delta/\sigma)_{max} < 0.001$
$S = 1.08$	$\Delta\rho_{max} = 0.17$ e Å^{-3}
7506 reflections	$\Delta\rho_{min} = -0.14$ e Å^{-3}
432 parameters	Absolute structure: Flack x determined using 2425 quotients [(I+)−(I−)]/[(I+)+(I−)] (Parsons, Flack and Wagner, *Acta Cryst. B69* (**2013**) 249−259).
11 restraints	Absolute structure parameter: −0.03 (8)
Primary atom site location: dual	
